# Supplementary material for: The dietary effects of two strain probiotics (Leuconostoc mesenteroides, Lactococcus lactis) on growth performance, immune response and gut microbiota in Nile tilapia (Oreochromis niloticus)
Source: PLoS One. 2024 Oct 24;19(10):e0312580. doi: 10.1371/journal.pone.0312580 (PMC11500904; doi:10.1371/journal.pone.0312580)

PLOS ONE

The dietary effect of multistrain probiotics (Leuconostoc mesenteroides, Lactococcus lactis) on growth performance, immune response and gut microbiota in Nile tilapia (Oreochromis niloticus)

--Manuscript Draft--

**Manuscript Number:**	PONE-D-24-34289
**Article Type:**	Research Article
**Full Title:**	The dietary effect of multistrain probiotics (Leuconostoc mesenteroides, Lactococcus lactis) on growth performance, immune response and gut microbiota in Nile tilapia<br><br>(Oreochromis niloticus)
**Short Title:**	The dietary effect of multistrain probiotics
**Corresponding Author:**	Svetlana Bayantassova<br>Zhengir Khan West-Kazakhstan Agrarian Technical University<br>Oral, KAZAKHSTAN
**Keywords:**	Nile tilapia; Leuconostoc mesenteroides; Lactococcus lactis; probiotic; immunity; growth.
**Abstract:**	The aquaculture industry has been growing rapidly over the past two decades to meet future protein demands. However, intensive aquaculture industry faces challenges such as growth abnormalities, high mortality rates, and intestinal health deterioration. Administering probiotics can serve as a nutritional strategy to enhance the immune system and growth performance of fish. This study aimed to evaluate the impact of multiple-strain dietary probiotics Leuconostoc mesenteroides and Lactococcus lactis on the growth performance, immunity, and gut microbiota of Nile tilapia (Oreochromis niloticus). Fish were fed with basal and experimental diet supplemented by both Leuconostoc mesenteroides and Lactococcus lactis bacteria at 106 cell/g for 8 weeks. Feeding a combination of Leuconostoc mesenteroides and Lactococcus lactis resulted in significant improvements in feed utilization parameters, alternative complement pathway activity, intestinal lactic acid bacteria count, mucus secretion and peroxidase activity compared to the control groups ($P < 0.05$). Serum lysozyme activity also exhibited a significant increase in the Leuconostoc mesenteroides and Lactococcus lactis dietary group compared to the control group. Furthermore, Nile tilapia fed the Leuconostoc mesenteroides and Lactococcus lactis diet showed enhanced growth performance metrics (weight gain, final weight and specific growth rate) compared to those fed the control diet ($P < 0.05$). Additionally, superoxide dismutase activity was significantly elevated in the Leuconostoc mesenteroides and Lactococcus lactis groups compared to the control group ($P < 0.05$). These findings strongly indicate that a dietary combination of Leuconostoc mesenteroides and Lactococcus lactis probiotics could function as a beneficial immunostimulant feed supplement in Nile tilapia aquaculture.
**Order of Authors:**	Assel Paritova
	Akylbek Nurgaliyev
	Gulbaram Nurgaliyeva
	Nurzhan Abekeshev
	Altynay Abuova
	Faruza Zakirova
	Grzegorz Zwierzchowski
	Zhaxygali Kuanchaleyev
	Saltanat Issabekova
	Maigul Kizatova

Powered by Editorial Manager® and ProduXion Manager® from Aries Systems Corporation

	Zaure Sayakova
	Dinara Zhanabayeva
	Yelena Kukhar
	Ruslan Stozhkov
	Botagoz Aitkozhina
	Yevgeniy Mayer
	Svetlana Bayantassova
	Angsar Satbek
	Alexandr Andruchshak
	Kaissar Kushaliyev
**Additional Information:**	
**Question**	**Response**
**Financial Disclosure**<br><br>Enter a financial disclosure statement that describes the sources of funding for the work included in this submission. Review the submission guidelines for detailed requirements. View published research articles from *PLOS ONE* for specific examples.<br><br>This statement is required for submission and **will appear in the published article** if the submission is accepted. Please make sure it is accurate.<br><br>**Funded studies**<br>Enter a statement with the following details:<br>• Initials of the authors who received each award<br>• Grant numbers awarded to each author<br>• The full name of each funder<br>• URL of each funder website<br>• Did the sponsors or funders play any role in the study design, data collection and analysis, decision to publish, or preparation of the manuscript?<br><br>Did you receive funding for this work?	Yes
Please add funding details.<br>as follow-up to "**Financial Disclosure**<br><br>Enter a financial disclosure statement that describes the sources of funding for the	This research was funded by the Science Committee of the Ministry of Science and Higher Education of the Republic of Kazakhstan (grant no. AP19576848)

Powered by Editorial Manager® and ProduXion Manager® from Aries Systems Corporation

work included in this submission. Review the submission guidelines for detailed requirements. View published research articles from *PLOS ONE* for specific examples.<br><br>This statement is required for submission and **will appear in the published article** if the submission is accepted. Please make sure it is accurate.<br><br>**Funded studies**<br>Enter a statement with the following details:<br>• Initials of the authors who received each award<br>• Grant numbers awarded to each author<br>• The full name of each funder<br>• URL of each funder website<br>• Did the sponsors or funders play any role in the study design, data collection and analysis, decision to publish, or preparation of the manuscript?<br><br>Did you receive funding for this work?"	
Please select the country of your main research funder (please select carefully as in some cases this is used in fee calculation).<br>as follow-up to "**Financial Disclosure**<br><br>Enter a financial disclosure statement that describes the sources of funding for the work included in this submission. Review the submission guidelines for detailed requirements. View published research articles from *PLOS ONE* for specific examples.<br><br>This statement is required for submission and **will appear in the published article** if the submission is accepted. Please make sure it is accurate.	KAZAKHSTAN - KZ

**Funded studies**<br>Enter a statement with the following details:<br>• Initials of the authors who received each award<br>• Grant numbers awarded to each author<br>• The full name of each funder<br>• URL of each funder website<br>• Did the sponsors or funders play any role in the study design, data collection and analysis, decision to publish, or preparation of the manuscript?<br><br>Did you receive funding for this work?"	
**Competing Interests**<br><br>Use the instructions below to enter a competing interest statement for this submission. On behalf of all authors, disclose any competing interests that could be perceived to bias this work—acknowledging all financial support and any other relevant financial or non-financial competing interests.<br><br>This statement is required for submission and **will appear in the published article** if the submission is accepted. Please make sure it is accurate and that any funding sources listed in your Funding Information later in the submission form are also declared in your Financial Disclosure statement.<br><br>View published research articles from *PLOS ONE* for specific examples.	The authors have declared that no competing interests exist.

Powered by Editorial Manager® and ProduXion Manager® from Aries Systems Corporation

**NO authors have competing interests**<br><br>Enter: *The authors have declared that no competing interests exist.*<br><br>**Authors with competing interests**<br><br>Enter competing interest details beginning with this statement:<br><br>*I have read the journal's policy and the authors of this manuscript have the following competing interests: [insert competing interests here]*<br><br>* typeset	
**Ethics Statement**<br><br>Enter an ethics statement for this submission. This statement is required if the study involved:<br><br>• Human participants<br>• Human specimens or tissue<br>• Vertebrate animals or cephalopods<br>• Vertebrate embryos or tissues<br>• Field research<br><br>Write "N/A" if the submission does not require an ethics statement.<br><br>General guidance is provided below. Consult the submission guidelines for detailed instructions. **Make sure that all information entered here is included in the Methods section of the manuscript.**	The protocol was approved by the Committee on the Ethics of Animal Experiments of the Saken Seifullin Kazakh Agrotechnical University (permit number: 2).

Powered by Editorial Manager® and ProduXion Manager® from Aries Systems Corporation

**Format for specific study types**<br><br>**Human Subject Research (involving human participants and/or tissue)**<br>• Give the name of the institutional review board or ethics committee that approved the study<br>• Include the approval number and/or a statement indicating approval of this research<br>• Indicate the form of consent obtained (written/oral) or the reason that consent was not obtained (e.g. the data were analyzed anonymously)<br><br>**Animal Research (involving vertebrate animals, embryos or tissues)**<br>• Provide the name of the Institutional Animal Care and Use Committee (IACUC) or other relevant ethics board that reviewed the study protocol, and indicate whether they approved this research or granted a formal waiver of ethical approval<br>• Include an approval number if one was obtained<br>• If the study involved *non-human primates*, add *additional details* about animal welfare and steps taken to ameliorate suffering<br>• If anesthesia, euthanasia, or any kind of animal sacrifice is part of the study, include briefly which substances and/or methods were applied<br><br>**Field Research**<br><br>Include the following details if this study involves the collection of plant, animal, or other materials from a natural setting:<br>• Field permit number<br>• Name of the institution or relevant body that granted permission	
**Data Availability**<br><br>Authors are required to make all data underlying the findings described fully available, without restriction, and from the time of publication. PLOS allows rare exceptions to address legal and ethical concerns. See the PLOS Data Policy and FAQ for detailed information.	Yes - all data are fully available without restriction

A Data Availability Statement describing where the data can be found is required at submission. Your answers to this question constitute the Data Availability Statement and **will be published in the article**, if accepted.<br><br>**Important:** Stating 'data available on request from the author' is not sufficient. If your data are only available upon request, select 'No' for the first question and explain your exceptional situation in the text box.<br><br>Do the authors confirm that all data underlying the findings described in their manuscript are fully available without restriction?	
**Describe where the data may be found in full sentences. If you are copying our sample text, replace any instances of XXX with the appropriate details.**<br><br>• If the data are **held or will be held in a public repository**, include URLs, accession numbers or DOIs. If this information will only be available after acceptance, indicate this by ticking the box below. For example: *All XXX files are available from the XXX database (accession number(s) XXX, XXX.*).<br>• If the data are all contained **within the manuscript and/or Supporting Information files**, enter the following: *All relevant data are within the manuscript and its Supporting Information files.*<br>• If neither of these applies but you are able to provide **details of access elsewhere**, with or without limitations, please do so. For example:<br><br>*Data cannot be shared publicly because of [XXX]. Data are available from the XXX Institutional Data Access / Ethics Committee (contact via XXX) for researchers who meet the criteria for access to confidential data.*<br><br>*The data underlying the results presented in the study are available from (include the name of the third party*	All relevant data are within the manuscript and its Supporting Information files.

and contact information or URL).<br>• This text is appropriate if the data are owned by a third party and authors do not have permission to share the data.<br><br>* typeset	
Additional data availability information:	

Powered by Editorial Manager® and ProduXion Manager® from Aries Systems Corporation

Manuscript
Click here to access/download;Manuscript;Manuscript.docx

The dietary effect of multistrain probiotics (*Leuconostoc mesenteroides, Lactococcus lactis*) on growth performance, immune response and gut microbiota in Nile tilapia (*Oreochromis niloticus*)

Assel Paritova[1], Akylbek Nurgaliyev[2], Gulbaram Nurgaliyeva[2], Nurzhan Abekeshev[2], Altynay Abuova[3], Faruza Zakirova[2], Grzegorz Zwierzchowski[4], Zhaxygali Kuanchaleyev[1], Saltanat Issabekova[1], Maigul Kizatova[5], Zaure Sayakova[6], Zhanabayeva Dinara[1], Yelena Kukhar[1], Ruslan Stozhkov[1], Botagoz Aitkozhina[1], Yevgeniy Mayer[1], Svetlana Bayantassova[2*], Angsar Satbek[1], Alexandr Andruchshak[1], Kaissar Kushaliyev[2*].

1. Saken Seifullin Kazakh Agrotechnical University, Astana, 010000, Kazakhstan.
2. Zhangir Khan West Kazakhstan Agrarian–Technical University, Uralsk 090000, Kazakhstan.
3. International Engineering and Technological University, Almaty, 050000, Kazakhstan.
4. University of Warmia and Mazury, Olsztyn, 10-719, Poland.
5. S.D. Asfendiyarov Kazakh National Medical University, 050000, Almaty, Kazakhstan.
6. Kazakh Scientific Veterinary Research Institute, 500016, Almaty, Kazakhstan.

*** Corresponding authors:** 1. Svetlana Bayantassova, bayantasova65@mail.ru

2. Kaissar Kushaliyev, kaissark@mail.ru

Abstract

The aquaculture industry has been growing rapidly over the past two decades to meet future protein demands. However, intensive aquaculture industry faces challenges such as growth abnormalities, high mortality rates, and intestinal health deterioration. Administering probiotics can serve as a nutritional strategy to enhance the immune system and growth performance of fish. This study aimed to evaluate the impact of multiple-strain dietary probiotics *Leuconostoc mesenteroides* and *Lactococcus lactis* on the growth performance, immunity, and gut microbiota of Nile tilapia (*Oreochromis niloticus*). Fish were fed with basal and experimental diet supplemented by both *Leuconostoc mesenteroides* and *Lactococcus lactis* bacteria at 10^6 cell/g

for 8 weeks. Feeding a combination of *Leuconostoc mesenteroides* and *Lactococcus lactis* resulted in significant improvements in feed utilization parameters, alternative complement pathway activity, intestinal lactic acid bacteria count, mucus secretion and peroxidase activity compared to the control groups ($P < 0.05$). Serum lysozyme activity also exhibited a significant increase in the *Leuconostoc mesenteroides* and *Lactococcus lactis* dietary group compared to the control group. Furthermore, Nile tilapia fed the *Leuconostoc mesenteroides* and *Lactococcus lactis* diet showed enhanced growth performance metrics (weight gain, final weight and specific growth rate) compared to those fed the control diet ($P < 0.05$). Additionally, superoxide dismutase activity was significantly elevated in the *Leuconostoc mesenteroides* and *Lactococcus lactis* groups compared to the control group ($P < 0.05$). These findings strongly indicate that a dietary combination of *Leuconostoc mesenteroides* and *Lactococcus lactis* probiotics could function as a beneficial immunostimulant feed supplement in Nile tilapia aquaculture.

Keywords: Nile tilapia, *Leuconostoc mesenteroides, Lactococcus lactis,* probiotic, immunity, growth.

Introduction

Over the past two decades, there has been considerable growth in the aquaculture industry, positioning it to effectively address the future protein demands of the human population [1]. Nonetheless, intensive aquaculture encounters challenges that have raised concerns regarding fish health, encompassing growth abnormalities, mortality rates, and disease outbreaks [2]. Consequently, farmers are forced to use prophylactic antibiotics to mitigate these challenges [3]. Antibiotics are commonly employed in aquaculture to manage diseases and enhance growth performance. However, their usage in this context has the potential to disrupt intestinal microbiota and foster the emergence of antibiotic-resistant bacteria, posing risks to aquatic life [4]. Consequently, previous studies aimed to devise alternative feed additives to substitute antibiotics in aquaculture practices [5-7]. The application of probiotics as dietary additives has

been recently proposed as a viable alternative to antibiotics. Their application aims to reduce the colonization gastrointestinal mucosa by pathogenic bacteria, thereby enhancing the health of mammals and fish [8-10]. Additionally, lactic acid bacteria have the capability to enhance growth performance and survival rates, prevent intestinal disorders such as diarrhea, neutralize antinutritional factors [11]. Moreover, advantageous intestinal microbiota can produce enzymes that have the potential to enhance the digestibility and uptake of dietary components [12]. It was documented that incorporating a combination of probiotics into the diet of *Labeo rohita* fingerlings resulted in improved nutrient digestibility, enhanced growth, a higher protein efficiency ratio, and a reduced feed conversion ratio compared to the use of individual strains [13]. In this study, we have used a combination of two probiotics *Leuconostoc mesenteroides* and *Lactococcus lactis* included in the diet of Nile tilapia. These probiotics were isolated from gastrointestinal tract of different fish species.

This study examined the effect of multiple strain dietary probiotics in freshwater aquaculture on growth performance, cultivable gut bacteria, and innate immune response, as well as body weight gain in Nile tilapia.

Materials and Methods

Animal Ethics

The experiment was carried out according to the European Convention for the Protection of Vertebrate Animals used for Experimental and Other Scientific Purposes guidelines [14]. The protocol was approved by the Committee on the Ethics of Animal Experiments of the Saken Seifullin Kazakh Agrotechnical University (permit number: 2).

Experimental design

Overall, 192 healthy juvenile Nile tilapia with body weight averaging 45±2g were purchased from local commercial fish farm. Fish were then equally divided in two groups in 500L tank: control group 96 heads and experimental group 96 heads respectively. Subsequently, the fish were randomly distributed into six 100-liter tanks, with thirty-two fish per tank and two replicate

tanks per treatment. Each tank was fitted with both an inlet and an outlet and maintained in a flow-through freshwater system. Prior experiment onset, fish allowed to acclimatize in 500L tank with 5mg/L O_2 aerated water for 14 days. Water quality parameters were assessed by employing a thermometer to measure temperature and an oxygen meter (Model FEBO 330, EPF Instruments, Fisher Scientific, USA) to measure dissolved oxygen levels. Throughout the experiment, water temperature was maintained at 27°C. The pH level was monitored with a portable pH meter (Fisherbrand AP 115). During the acclimatization period fish were fed a control diet without probiotic supplements *ad libitum* twice a day at 9:00 a.m. and 17:00 p.m. After completing the feeding trial (8 weeks), all experimental fish subjected a 24-hour fasting. Following this, survival rate, individual body weight and length, immune parameters, and gut microbiota composition of fish from each tank were determined.

Isolation of Probiotics

Leuconostoc mesenteroides and *Lactococcus lactis* LAB starins were isolated from intestines of common carp (*Cyprinus carpio*) fingerlings following protocol described by [2]. In brief, a 50 µl sample of intestinal microflora from fish was dissolved in 500 µl distilled water (1:10 ratio). Subsequently, using a disposable pipette, 100 µl of the suspension was introduced to the MRS agar medium and incubated at 37°C for 48 h. Glass beads were utilized to ensure uniform growth of isolated colonies. Eight to twelve glass beads (pre-sterilized) were placed into the Petri dish containing the inoculum. The probiotics obtained were washed three times in distilled water and plates were sent for genomic confirmation.

PCR

For DNA extraction, GeneJET PCR Purification Kit (Thermo Fisher Scientific, USA) was used according to the manufacturer's instructions. Following reference strains were utilized as a positive controls: National Collection of Type Culture (NCTC) AB023246 for *Leuconostoc mesenteroides* and (NCTC) M58837 for *Lactococcus lactis*. For genotyping of isolates, multiplex PCR (mPCR) was run according to the protocol described by Ali, Kot [15]. The

amplification of 16S mDNA fragments in a Thermal Cycler (Eppendorf Mastercycler) were as follows: 95 ◦C for 2 min 30 s, 95 ◦C for 1 min, 55 ◦C for 1 min, 72 ◦C for 1 min 20 s (35 cycles), and 72 ◦C for 2 min. The PCR products obtained were administered in 1% agarose gel electrophoresis in the presence of *Ethidium bromide*, and the results were visualized using Bio-Imaging Systems (MiniBIS Pro, Israel).

Preparation of Experimental Diets

Commercial feed without probiotic supplements was used as a basal control diets. Basal diet composed of fishmeal, soybean meal, milled wheat, extruded peas, potato starch, vegetable oil, tricalcium phosphate, premix, yeast (Table 1).

The experimental diet was prepared by introducing 1×10^7 CFU/g of each Leuconostoc mesenteroides and Lactococcus lactis in the basal diet. To do so, these LAB were incorporated into the basal diet using the spraying method while stirring. Then, 165g of gelatin was dissolved in 500 ml of distilled water to create a waterproof membrane for the feed. Next, the dietary components underwent grinding into a fine powder, followed by thorough agitation and subsequent cold extrusion into pellets 0.5 mm in diameter. Finally, the diluted gelatin was sprayed onto LAB incorporated basal diet pellets under thorough stirring. The complete diet were then air-dried overnight at room temperature and stored in plastic containers at −4°C until required.

Table 1. Formulation of the basal diet for Nile tilapia, *Oreochromis niloticus*.

Feed ingredients	g kg^{-1}	%
Fishmeal	30	3
Soybean meal	42	4.2
Milled wheat	7	0.7
Extruded peas	8	0.8
Potato starch	6.7	0.67
Vegetable oil	4	0.4
Vitamin/Mineral mixture	1.1	0.11
Premix	0.1	0.01

Yeast	0.05	0.005
Gelatin	1	0.1
Proximate composition %		
Moisture	8.79	
Crude protein	35.8	
Crude lipid	7.39	
Crude ash	6.44	

Growth performance

The calculation of weight gain (WG), feed conversion ratio (FCR), protein efficiency ratio (PER) and specific growth rate (SGR) were performed according to protocol described by Allameh, Yusoff [16]. In brief, WG in grams is calculated by subtracting the initial weight from the final weight. SGR is determined by the formula: SGR = 100 (ln W2 - ln W1) / T, where W1 and W2 represent the initial and final weights, respectively, and T signify the number of days during the feeding period. SGR indicates the percentage of growth per day for each fish.FCR is computed as the ratio of feed intake in grams to weight gain in grams, while PER is determined by the ratio of weight gain in grams to protein intake in grams.

Determination of Intestinal bacteria activities

To determine the total intestinal microbiota and LAB count, eleven fish were sampled from each study group following 24 h feeding cessation. Prior experiment onset, fish were anesthetized using tricaine methanesulphonate (MS 222) and then transferred on to sterile stainless desk. Before opening, fish carcasses were rinsed with 70% ethanol to eliminate surface contamination. The whole intestine was extracted, and 1 cm sections from both the anterior and posterior ends were discarded. Then the whole intestinal tract was dissected, and rinsed three times in PBS. Subsequently, tenfold serial dilution was run in PBS. 100 ml aliquots of the diluted solution were then plated onto duplicate Trypto-Soya agar (TSA) media to determine total bacterial counts. To isolate viable LAB species, 100 ml of each dilution was introduced in MRS (DeMan, Rogosa, and Sharpe) agar and allowed to incubate at 25°C for 3 to 5 days. Colony-forming units of

viable bacterial populations were enumerated according to protocol reported by Ramos, Batista [17].

Immune responses

Alternative complement pathway activity (ACH50) was determined according to protocol of Yano [18]. In brief, 0.1ml aliquots of diluted serum were transferred in to plastic tubes and adjusted to a total volume of 0.25 ml using barbitone buffer. Rabbit red blood cells (RaRBC) were then added to each tube in a volume of 0.1 ml and allowed to incubate for 1h at 20°C. Next, after incubation, 3.15 ml of 0.9% NaCl solution was introduced and samples were centrifugated at 1600 x g for 10 minutes at 4°C to remove RaRBC derbies. Finally, obtained supernatant was measured for optical density at 414 nm, and a lysis curve was generated. The volume resulting in 50% hemolysis was identified and utilized to assess the complement activity of the sample.

The bactericidal activity of serum and mucus was assessed following the method described by Dawood, Koshio [19]. To do so, samples were diluted in 1:1 ratio with Tris buffer adjusted to pH 7.5. Then, diluted mixture was agitated with pre-prepared 0.001 mg/ml Escherichia coli, IAM1239 bacterial suspension (RIBSP, Kazakhstan) and allowed to incubate overnight at room temperature. The standard plate counting method was employed to calculate colony-forming units (CFU).

To measure lysozyme activity in serum and mucus samples a turbidimetric assay was utilized. Briefly, 10µl of samples were mixed with 190 µl of Micrococcus lysodeikticus bacterium (0.2 mg/PBS adjusted to pH 7.4) in 96 flat-bottomed well plates, and the mixture was allowed to incubate for 5 min at room temperature. Absorbance was measured at 450 nm using Biotec 800 TS (Agilent Technologies, USA). Lysosome activity was determined based on the amount of sample resulting in a decrease in optical density of 0.001 min^{-1}.

The total peroxidase content in serum was assessed according to method described by Salinas, Abelli [20]. To do this, 15 µl of serum and 35 µl of Hank's Buffered Salt Solution were added to 96 flat-bottomed well plates. Subsequently, 50 µl of 3,3′,5,5′-Tetramethylbenzidine (TMB,

Thermo Scientific, USA) was added to each well. The diluted serum was then incubated for 20 minutes, and the color-developing reaction was halted by introducing 50 µl of 2 M sulfuric acid. The optical density was measured at a wavelength of 450 nm using a plate reader.

Serum superoxide dismutase (SOD) activity was quantified by the percentage inhibition rate of the enzyme's reaction using Ransod kit (Randox, Crumlin, UK) following the protocol described by Sun, Yang [21]. The specific SOD activity units were adjusted by milligrams of protein (mg protein)$^{-1}$. One unit of SOD is determined as the quantity of the enzyme present in 20 ml of the sample solution, which inhibits the reduction reaction of nitroblue tetrazolium (NBT) by 50% at wavelength of 550 nm.

Statistical analysis

Graphs were compiled and statistical analyses were conducted to protocol described by Bayantassova, Kushaliyev [22] using Prism 9 software (GraphPad Software, Inc., San Diego, CA). The data underwent evaluation through a variety of statistical tests, including Student's t-test, one-way ANOVA with Tukey’s post hoc analysis. Statistical significance between groups was denoted when $P < 0.05$ using asterisks, as detailed in the respective figures.

Results

Demonstration of growth performance, nutrient utilization and survival.

The growth parameters, feed uptake and survival of control and fish fed *Leuconostoc mesenteroides* and *Lactococcus lactis* diet are presented in Table 2. Fish fed the *Leuconostoc mesenteroides* and *Lactococcus lactis* diet demonstrated a substantial increase in weight gain (WG), final body weight (FBW), and specific growth rate (SGR) ($P \leq 0.05$) compared to the control group. Additionally, the protein efficiency ratio (PER) and feed efficiency ratio (FER) profiles were significantly higher in fish fed on mixture of *Leuconostoc mesenteroides* and *Lactococcus lactis* diet ($P \leq 0.05$). Moreover, a significant increase in the protein gain (PG) profile was observed in fish fed *Leuconostoc mesenteroides* and *Lactococcus lactis*-containing diet compared to the control group ($P \leq 0.05$). Fish fed on experimental diet showed higher feed intake

(FI) profile, however no significant difference was observed between study groups ($P \geq 0.05$). Survival rates were high across all treatment groups, and no mortality was observed.

Table 2. Growth performance of Nile tilapia (Oreochromis niloticus) fed with *Leuconostoc mesenteroides* and *Lactococcus lactis* supplemented diets for 8 weeks.

	Diets		*P* - value
	Control	Mixture of *Leuconostoc mesenteroides* and *Lactococcus lactis* probiotics	
[1]IBW (g)	46.3±1.32	45.8±2.11	0.902
[2]FBW (g)	68.4±3.26	92.4±4.54*	0.034
[3]WG (%)	22.4±3.01	47.3±2.02*	0.042
[4]SGR (%)	1.96±0.11	2.82±0.2*	0.03
[5]FI (g)	12.42±0.47	13.24±0.8	0.998
[6]FER	2.33±0.2	2.97±0.5*	0.024
[7]PER	3.05±0.4	3.65±0.03*	0.037
[8]PG	141.23±7.2	179.31±4.05*	0.012

[1]IBW - initial body weight; [2]FBW - final body weight; [3]WG - weight gain; [4]SGR - specific growth rate; [5]FI – feed intake; [6]FER - feed efficiency ratio; [7]PER - protein efficiency ratio; [8]PG - protein gain. * p - value ≤ 0.05. Values represent means ± SEM of triplicate groups.

Microbiological analyses of gut microbiota

Both the control and experimental groups showed no detectable LAB bacteria in the intestines before the experiment. However, fish fed LAB-supplemented diets demonstrated significantly greater levels of *Leuconostoc mesenteroides and Lactococcus lactis* in the total intestinal microbiota compared to the control group ($P < 0.05$) (Figure 1). Moreover, no LAB bacteria were detectable in the intestine of fish fed control diet.

Figure 1. Comparison of total bacteria and LAB content of Nile tilapia intestines fed with control and experimental diets for 8 weeks. Data presented as the mean ±SEM from two combined independent experiments (n=10 fish/group). Bars with different alphabet are statistically different ($P \leq 0.05$). Bars with the same alphabet are statistically insignificant different ($P \geq 0.05$). * LM; LL - *Leuconostoc mesenteroides*; *Lactococcus lactis*.

Demonstration of Immune responses

The rate of alternative complement pathway (ACP) were significantly elevated in fish fed *Leuconostoc mesenteroides* and *Lactococcus lactis* supplemented diets than that of control group (Figure 2). Likewise, serum and mucus bactericidal activity (Figure 3), mucus lysozyme activity (Figure 4), peroxidase activity (Figure 5), superoxide dismutase (Figure 6) demonstrated a considerable elevation in fish fed experimental diets compared to control group. Additionally, when compared with the control group, lysozyme activities of serum was significantly higher in fish fed mixture of *Leuconostoc mesenteroides* and *Lactococcus lactis* diets ($P \leq 0.05$; Figure 4). Furthermore, the amounts of secreted mucus was significantly higher in fish supplemented with *Leuconostoc mesenteroides* and *Lactococcus lactis* diets ($P \leq 0.05$; Figure 7).

Figure 2. Alternative complement pathway of Nile tilapia fed with control and experimental diets. Data presented as the mean ±SEM from two combined independent experiments (n=10 fish/group). Bars with different alphabet are statistically different ($P \leq 0.05$). Bars with the same alphabet are statistically insignificant different ($P \geq 0.05$). * LM; LL - *Leuconostoc mesenteroides*; *Lactococcus lactis*.

Figure 3. Serum and mucus bactericidal activity of Nile tilapia fed with control and experimental diets. Data presented as the mean ±SEM from two combined independent experiments (n=10 fish/group). Bars with different alphabet are statistically different ($P \leq 0.05$). Bars with the same alphabet are statistically insignificant different ($P \geq 0.05$).

* LM; LL - *Leuconostoc mesenteroides*; *Lactococcus lactis*.

Figure 4. Serum and mucus lysozyme activity of Nile tilapia fed with control and experimental diets. Data presented as the mean ±SEM from two combined independent experiments (n=10 fish/group). Bars with different alphabet are statistically different ($P \leq 0.05$). Bars with the same alphabet are statistically insignificant different ($P \geq 0.05$). * LM; LL - *Leuconostoc mesenteroides*; *Lactococcus lactis*.

Figure 5. Serum peroxidase activity of Nile tilapia fed with control and experimental diets. Data presented as the mean ±SEM from two combined independent experiments (n=10 fish/group). Bars with different alphabet are statistically different ($P \leq 0.05$). Bars with the same alphabet are statistically insignificant different ($P \geq 0.05$). * LM; LL - *Leuconostoc mesenteroides*; *Lactococcus lactis*.

Figure 6. Superoxide dismutase activity of Nile tilapia fed with control and experimental diets. Data presented as the mean ±SEM from two combined independent experiments (n=10 fish/group). Bars with different alphabet are statistically different ($P \leq 0.05$). Bars with the same alphabet are statistically insignificant different ($P \geq 0.05$). * LM; LL - - *Leuconostoc mesenteroides*; *Lactococcus lactis*.

Figure 7. The effect of experimental diet on mucus secretion. Data presented as the mean ±SEM from two combined independent experiments (n=10 fish/group). Bars with different alphabet are statistically different ($P \leq 0.05$). Bars with the same alphabet are statistically insignificant different ($P \geq 0.05$). * LM; LL - - *Leuconostoc mesenteroides*; *Lactococcus lactis*.

Discussion

An increasing body of research has illustrated the application of single lactic acid bacteria species as a probiotic treatment to enhance growth performance, immune response, feed utilization and gut microbiota modulation in fish [23-25]. However, there has been limited focus on exploring the potential synergistic effects that may arise from the concurrent oral administration of multiple LAB species to fish [2, 26-28]. Hence, this study was carried out to assess the dietary effect of *Leuconostoc mesenteroides* and *Lactococcus lactis* on the growth performance, immunity and gut microbiota of Nile tilapia (*Oreochromis niloticus*).

The findings of this study suggest that administering both *Leuconostoc mesenteroides* and *Lactococcus lactis* into supplementation diet significantly enhances the growth performance of Nile tilapia compared to the control group over a 8 weeks period. This outcome is consistent with previous research demonstrating that probiotic supplementation, particularly with *Lactobacillus* species, improves the growth performance of various fish species, including olive flounder (*P. olivaceus*) [27], Nile tilapia (*Oreochromis sp*) [29], gilthead sea bream (*Sparus aurata*) [30]. Furthermore, *L. lactis* has been found to enhance the growth performance of olive flounder (*Paralichthys olivaceus*) [31] and grouper (*Epinephelus coioides*) [32]. Additionally, the results obtained in this study showed that fish fed a mixture of *Leuconostoc mesenteroides* and *Lactococcus lactis* exhibited higher final weight, weight gain, and specific growth rate compared to the groups fed the control diet. Similarly, Allameh, Yusoff [16] reported that administering multistrain probiotics, particularly *Leuconostoc mesenteroides*, enhanced the growth performance of Javanese carp (*Puntius gonionotus*). In the current study, fish fed the diet supplemented with a mixture of *Leuconostoc mesenteroides* and *Lactococcus lactis* showed significant improvements in feed intake and feed utilization parameters. This observation suggests that the probiotic supplement may have influenced the palatability of the feed, consequently resulting in slight increases in the growth rate and feed intake of fish. To the best of our knowledge, this study represents the first dietary incorporation of *Leuconostoc*

mesenteroides and *Lactococcus lactis* leading to enhanced growth and feed utilization in Nile tilapia. Xia, Cao [33] proposed that probiotics primarily influence Nile tilapia by augmenting host nutrition, thereby stimulating digestive enzymes and ultimately leading to enhanced growth and feed efficiency ratios. Moreover, the colonization of probiotic microorganisms in the intestine enhances microbial equilibrium, subsequently improving feed utilization and nutrient absorption [34-36].

Improvements in growth performance and feed utilization may also be attributed to enhanced intestinal digestive functions in fish owing to probiotic supplementation (Leuconostoc mesenteroides and Lactococcus lactis), which likely includes increased activities of digestive enzymes. Previous studies have shown that supplementation with Lactobacillus species leads to improved nutrient digestibility [7]. It is well-established that the activity of digestive enzymes is positively correlated with the digestive capacity of fish [37], thereby enhancing their ability to extract nutrients from food [38]. It has been reported that digestive organs are highly sensitive to food composition, leading to immediate changes in the activities of digestive enzymes ultimately affecting fish growth and health [39].. Furthermore, bacteria secrete proteases to cleave peptide bonds in proteins, breaking them down into free amino acids and constituent monomers, thereby improving the animal's nutritional status. Bacterial enzymatic hydrolysis has been demonstrated to improve the bioavailability of lipids, protein and dry matter [40], potentially leading to the increased growth and nutrient utilization observed in this study.

The current study revealed a significant enhancement in both total intestinal bacteria and lactic acid bacteria counts, further supporting the improvements observed in growth and feed utilization. Similar to the findings of Mohapatra, Chakraborty [13], a substantial increase in total bacterial counts was noted when fish were fed diets supplemented with probiotics. This increase could potentially lead to improved fish health and immunity, thereby promoting growth. It has been documented that the colonization rate of bacteria in the digestive tract is directly influenced by the level of dietary bacteria intake [41]. In our investigation, the elevated adherence of

microorganisms introduced via dietary supplementation could explain the improved nutrient utilization and growth observed in the fish. Numerous studies have suggested that probiotics function by colonizing the host and secreting various growth-promoting nutrients [42] [43]. Previous studies have documented the crucial interplay between intestinal epithelial cells and probiotics in regulating the mucosal immunity of the fish gut. This interaction modulates both the physical and immunological barrier functions of the intestine and stimulates an immune response [44-48].

The results of the current investigation also indicate that the dietary combination of both *Leuconostoc mesenteroides* and *Lactococcus lactis* led to an enhanced immune response. These improvements included increased lysozyme and bactericidal activities in both serum and mucus, as well as enhancements in the alternative complement pathway, superoxide dismutase, and peroxidase activities. Likewise, the beneficial effect of combined probiotics in the diet have been documented to trigger innate immunity in rainbow trout (*Oncorhynchus mykiss*) [49], *Labeo rohita* [50], olive flounder (*P. olivaceus*) [27]. Others reported that combined utilization of bacterial strains with complementary properties within the gut environment can enhance or extend the favorable health-promoting effects and immune response of the host [51]. Several authors have reported that external factors such as probiotics, vitamins, and nutrients influence the activity of the alternative complement pathway in fish [52, 53]. In the present study, the activity of the alternative complement pathway emerged as one of the immune system parameters most affected, showing a significant increase in the treatment group. These findings are similar to those described by Giri, Sukumaran [26] and Sun, Yang [32].

It was reported that bactericidal activity is a critical component of the host's defence mechanisms against pathogens [7, 54]. The outcomes obtained in our study indicate that the greatest levels of serum and mucus bactericidal activity were observed in fish receiving diets supplemented with *Leuconostoc mesenteroides* and *Lactococcus lactis*. The lysozyme activities in fish have been shown to be influenced by various probiotic species. For instance, in brown trout (*Salmo trutta*),

supplementation with *Leuconostoc mesenteroides* has been reported to affect lysozyme activity [47]. Similarly, in grouper (*Epinephelus coioides*), supplementation with *Lactococcus lactis* has been found to modulate lysozyme activity [32]. Consistent with previous findings, in our study, the probiotics *Leuconostoc mesenteroides* and *Lactococcus* lactis have altered the serum lysozyme activity of Nile tilapia (*Oreochromis niloticus*). The *Leuconostoc mesenteroides* and *Lactococcus lactis* resulted in increased serum peroxidase values, while the peroxidase content remained unaffected by the mixture diet of *Leuconostoc mesenteroides* and *Lactococcus lactis*, which supports earlier observation in sea bream [20]. Superoxide dismutase facilitates the conversion of highly reactive superoxide radicals into less reactive hydrogen peroxide and plays a crucial role in the primary antioxidant defense mechanisms against oxidative stress. In our study, there was a significant enhancement in superoxide dismutase activities in the experimental group. Comparable findings were reported in *Labeo rohita*, where dietary supplementation with *Bacillus subtilis* in combination with *Pseudomonas aeruginosa* and *Lactobacillus plantarum*, resulted in similar improvements [26]. Fish possess a distinctive physical barrier comprising epidermal mucus and skin [55], which serves as a component of their nonspecific defense mechanisms, protecting the fish body from direct exposure to environmental stressors and pollutants in the surrounding water. The findings of this study demonstrated an increased secretion of mucus in all fish fed diets supplemented with *Leuconostoc mesenteroides* and *Lactococcus lactis* compared to those fed the control diet.

Together current findings showed that the diet supplemented with *Leuconostoc mesenteroides* and *Lactococcus lactis* species exerted more pronounced effects on the innate immune system of Nile tilapia. Consequently, formulations containing multiple probiotic strains were observed to elicit distinct responses in the host immune system compared to formulations containing single probiotic strains. The evidence from this study indicates that in higher vertebrates, well-designed formulations of multistrain or multispecies probiotics have been shown to exhibit health-promoting effects that are lacking in diets containing single-species probiotics [56, 57].

The effects of probiotics vary widely and are contingent upon factors such as their sources, dosage, types, and supplementation period. Therefore, when formulating a probiotic mixture, it is essential to thoroughly select candidate strains to optimize their synergistic effects.

The combination of *Leuconostoc mesenteroides* and *Lactococcus lactis* appears to be a suitable mixture in this context. The synergistic effect enabled by the mixture of *Leuconostoc mesenteroides* and *Lactococcus lactis* potentially contributed to the enhanced performance of Nile tilapia. However, further research is needed to confirm this potential.

Author Contributions: Supervision, conceptualization, writing—original draft preparation, writing—review and editing S.B., K.K.; methodology, validation, formal analysis, A.N., G.N., A.P., A.N., G.N., N.A., A.A., F.Z., G.Z., Zh.K., S.I., M.K., Z.S., Zh.D., Y.K., R.S., A.S., A.A. All authors have read and agreed to the published version of the manuscript.

Funding: This research was funded by the Science Committee of the Ministry of Science and Higher Education of the Republic of Kazakhstan (grant no. AP19576848)

Data Availability Statement: The datasets containing all data analyzed, supporting the results of this study will be available upon request from corresponding author.

References

format check

1. Tan, H.Y., S.W. Chen, and S.Y. Hu, *Improvements in the growth performance, immunity, disease resistance, and gut microbiota by the probiotic Rummeliibacillus stabekisii in Nile tilapia (Oreochromis niloticus).* Fish Shellfish Immunol, 2019. **92**: p. 265-275.
2. Won, S., et al., *Effects of Bacillus subtilis WB60 and Lactococcus lactis on growth, immune responses, histology and gene expression in Nile tilapia, Oreochromis niloticus.* Microorganisms, 2020. **8**(1).
3. Defoirdt, T., et al., *Alternatives to antibiotics to control bacterial infections: luminescent vibriosis in aquaculture as an example.* Trends in biotechnology, 2007. **25**(10): p. 472-479.
4. Dawood, M.A., S. Koshio, and M.Á. Esteban, *Beneficial roles of feed additives as immunostimulants in aquaculture: a review.* Reviews in Aquaculture, 2018. **10**(4): p. 950-974.
5. Nayak, S.K. and S.C. Mukherjee, *Screening of gastrointestinal bacteria of Indian major carps for a candidate probiotic species for aquaculture practices.* Aquaculture Research, 2011. **42**(7): p. 1034-1041.
6. Liu, H., et al., *Isolation of a putative probiotic strain S12 and its effect on growth performance, non-specific immunity and disease-resistance of white shrimp, Litopenaeus vannamei.* Fish & shellfish immunology, 2014. **41**(2): p. 300-307.
7. Dawood, M.A., et al., *Interaction effects of dietary supplementation of heat-killed Lactobacillus plantarum and β-glucan on growth performance, digestibility and immune response of juvenile red sea bream, Pagrus major.* Fish & shellfish immunology, 2015. **45**(1): p. 33-42.
8. Merrifield, D.L., et al., *Indigenous lactic acid bacteria in fish and crustaceans.* Aquaculture nutrition: gut health, probiotics and prebiotics, 2014: p. 128-168.
9. Šušković, J., et al., *Antimicrobial activity–the most important property of probiotic and starter lactic acid bacteria.* Food Technology and Biotechnology, 2010. **48**(3): p. 296-307.
10. Seal, B.S., et al., *Alternatives to antibiotics: a symposium on the challenges and solutions for animal production.* Animal Health Research Reviews, 2013. **14**(1): p. 78-87.
11. Dalié, D., A. Deschamps, and F. Richard-Forget, *Lactic acid bacteria–Potential for control of mould growth and mycotoxins: A review.* Food control, 2010. **21**(4): p. 370-380.
12. Ray, A.K., K. Ghosh, and E. Ringø, *Enzyme-producing bacteria isolated from fish gut: a review.* Aquaculture nutrition, 2012. **18**(5): p. 465-492.
13. Mohapatra, S., et al., *Use of different microbial probiotics in the diet of rohu, Labeo rohita fingerlings: effects on growth, nutrient digestibility and retention, digestive enzyme activities and intestinal microflora.* Aquaculture Nutrition, 2012. **18**(1): p. 1-11.
14. Ausems, E.J., *THE EUROPEAN CONVENTION FOR THE PROTECTION OF VERTEBRATE ANIMALS USED FOR EXPERIMENTAL AND OTHER SCIENTIFIC PURPOSES.* Zeitschrift Fur Versuchstierkunde, 1986. **28**(5): p. 219-219.
15. Ali, Y., et al., *Classification of lytic bacteriophages attacking dairy Leuconostoc starter strains.* Applied and environmental microbiology, 2013. **79**(12): p. 3628-3636.
16. Allameh, S.K., et al., *Effects of dietary mono- and multiprobiotic strains on growth performance, gut bacteria and body composition of Javanese carp (Puntius gonionotus, Bleeker 1850).* Aquaculture Nutrition, 2016. **22**(2): p. 367-373.
17. Ramos, M.A., et al., *Dietary probiotic supplementation improves growth and the intestinal morphology of Nile tilapia.* Animal, 2017. **11**(8): p. 1259-1269.
18. Yano, T., *Assay of hemolytic complement activity.* Techniques in fish immunology, 1992: p. 131-141.
19. Dawood, M.A., et al., *Effects of dietary supplementation of Lactobacillus rhamnosus or/and Lactococcus lactis on the growth, gut microbiota and immune responses of red sea bream, Pagrus major.* Fish Shellfish Immunol, 2016. **49**: p. 275-85.
20. Salinas, I., et al., *Monospecies and multispecies probiotic formulations produce different systemic and local immunostimulatory effects in the gilthead seabream (Sparus aurata L.).* Fish & shellfish immunology, 2008. **25**(1-2): p. 114-123.

21. Sun, Y.-Z., et al., *Probiotic applications of two dominant gut Bacillus strains with antagonistic activity improved the growth performance and immune responses of grouper Epinephelus coioides.* Fish & shellfish immunology, 2010. **29**(5): p. 803-809.
22. Bayantassova, S., et al., *Knowledge, attitude and practice (KAP) of smallholder farmers on foot-and-mouth disease in Cattle in West Kazakhstan.* Vet Med Sci, 2023. **9**(3): p. 1417-1425.
23. Sun, Y., et al., *Effects of dietary administration of Lactococcus lactis HNL12 on growth, innate immune response, and disease resistance of humpback grouper (Cromileptes altivelis).* Fish & shellfish immunology, 2018. **82**: p. 296-303.
24. Hooshyar, Y., et al., *Effects of Lactobacillus rhamnosus ATCC 7469 on Different Parameters Related to Health Status of Rainbow Trout (Oncorhynchus mykiss) and the Protection Against Yersinia ruckeri.* Probiotics Antimicrob Proteins, 2020. **12**(4): p. 1370-1384.
25. Adeshina, I., M.I. Abubakar, and B.E. Ajala, *Dietary supplementation with Lactobacillus acidophilus enhanced the growth, gut morphometry, antioxidant capacity, and the immune response in juveniles of the common carp, Cyprinus carpio.* Fish Physiol Biochem, 2020. **46**(4): p. 1375-1385.
26. Giri, S.S., et al., *Effects of dietary supplementation of potential probiotic Bacillus subtilis VSG1 singularly or in combination with Lactobacillus plantarum VSG3 or/and Pseudomonas aeruginosa VSG2 on the growth, immunity and disease resistance of Labeo rohita.* Aquaculture Nutrition, 2014. **20**(2): p. 163-171.
27. Beck, B.R., et al., *The effects of combined dietary probiotics Lactococcus lactis BFE920 and Lactobacillus plantarum FGL0001 on innate immunity and disease resistance in olive flounder (Paralichthys olivaceus).* Fish Shellfish Immunol, 2015. **42**(1): p. 177-83.
28. Zhang, H., et al., *Effect of Dietary Supplementation of Lactobacillus Casei YYL3 and L. Plantarum YYL5 on Growth, Immune Response and Intestinal Microbiota in Channel Catfish.* Animals (Basel), 2019. **9**(12).
29. Xia, Y., et al., *Effects of dietary Lactobacillus rhamnosus JCM1136 and Lactococcus lactis subsp. lactis JCM5805 on the growth, intestinal microbiota, morphology, immune response and disease resistance of juvenile Nile tilapia, Oreochromis niloticus.* Fish Shellfish Immunol, 2018. **76**: p. 368-379.
30. Suzer, C., et al., *Lactobacillus spp. bacteria as probiotics in gilthead sea bream (Sparus aurata, L.) larvae: Effects on growth performance and digestive enzyme activities.* Aquaculture, 2008. **280**(1): p. 140-145.
31. Heo, W.-S., et al., *Effects of dietary probiotic, Lactococcus lactis subsp. lactis I2, supplementation on the growth and immune response of olive flounder (Paralichthys olivaceus).* Aquaculture, 2013. **376-379**: p. 20-24.
32. Sun, Y.-Z., et al., *Effect of Lactococcus lactis and Enterococcus faecium on growth performance, digestive enzymes and immune response of grouper Epinephelus coioides.* Aquaculture Nutrition, 2012. **18**(3): p. 281-289.
33. Xia, Y., et al., *Effects of Lactococcus lactis subsp. lactis JCM5805 on colonization dynamics of gut microbiota and regulation of immunity in early ontogenetic stages of tilapia.* Fish & Shellfish Immunology, 2019. **86**: p. 53-63.
34. Adeoye, A.A., et al., *Combined effects of exogenous enzymes and probiotic on Nile tilapia (Oreochromis niloticus) growth, intestinal morphology and microbiome.* Aquaculture, 2016. **463**: p. 61-70.
35. Lara-Flores, M. and M.A. Olvera-Novoa, *The use of lactic acid bacteria isolated from intestinal tract of Nile tilapia (Oreochromis niloticus), as growth promoters in fish fed low protein diets.* Latin American Journal of Aquatic Research, 2017. **41**(3): p. 490-497.
36. Lara-Flores, M., et al., *Use of the bacteria Streptococcus faecium and Lactobacillus acidophilus, and the yeast Saccharomyces cerevisiae as growth promoters in Nile tilapia (Oreochromis niloticus).* Aquaculture, 2003. **216**(1): p. 193-201.
37. Hakim, Y., et al., *Relationship between intestinal brush border enzymatic activity and growth rate in tilapias fed diets containing 30% or 48% protein.* Aquaculture, 2006. **257**(1-4): p. 420-428.
38. Furne, M., et al., *Digestive enzyme activities in Adriatic sturgeon Acipenser naccarii and rainbow trout Oncorhynchus mykiss. A comparative study.* Aquaculture, 2005. **250**(1-2): p. 391-398.

39. Shan, X., et al., *Effects of photoperiod on growth, mortality and digestive enzymes in miiuy croaker larvae and juveniles.* Aquaculture, 2008. **281**(1-4): p. 70-76.
40. Ling, W.H. and O. Hänninen, *Shifting from a conventional diet to an uncooked vegan diet reversibly alters fecal hydrolytic activities in humans.* The Journal of nutrition, 1992. **122**(4): p. 924-930.
41. Bagheri, T., et al., *Growth, survival and gut microbial load of rainbow trout (Onchorhynchus mykiss) fry given diet supplemented with probiotic during the two months of first feeding.* Turkish Journal of Fisheries and Aquatic Sciences, 2008(1): p. 43-48.
42. Carnevali, O., F. Maradonna, and G. Gioacchini, *Integrated control of fish metabolism, wellbeing and reproduction: The role of probiotic.* Aquaculture, 2017. **472**: p. 144-155.
43. Iorizzo, M., et al., *Probiotic Potentiality from Versatile Lactiplantibacillus plantarum Strains as Resource to Enhance Freshwater Fish Health.* Microorganisms, 2022. **10**(2).
44. Thomas, C.M. and J. Versalovic, *Probiotics-host communication: modulation of signaling pathways in the intestine.* Gut microbes, 2010. **1**(3): p. 148-163.
45. Wells, J.M., *Immunomodulatory mechanisms of lactobacilli.* Microbial cell factories, 2011. **10**(Suppl 1): p. S17.
46. Liu, W., et al., *Comparison of adhesive gut bacteria composition, immunity, and disease resistance in juvenile hybrid tilapia fed two different Lactobacillus strains.* Fish & shellfish immunology, 2013. **35**(1): p. 54-62.
47. Balcazar, J.L., et al., *Changes in intestinal microbiota and humoral immune response following probiotic administration in brown trout (Salmo trutta).* Br J Nutr, 2007. **97**(3): p. 522-7.
48. Seisenov, B., et al., *In Vitro Fertilization in Kazakh Whiteheaded Cattle: A Comparative Study.* Life (Basel), 2023. **13**(8): p. 1632.
49. Jasim, S.A., et al., *The synergistic effects of the probiotic (Lactobacillus fermentum) and cinnamon, Cinnamomum sp. powder on growth performance, intestinal microbiota, immunity, antioxidant defence and resistance to Yersinia ruckeri infection in the rainbow trout (Oncorhynchus mykiss) under high rearing density.* Aquaculture Research, 2022. **53**(17): p. 5957-5970.
50. Devi, G., et al., *Effect of symbiotic supplemented diet on innate-adaptive immune response, cytokine gene regulation and antioxidant property in Labeo rohita against Aeromonas hydrophila.* Fish & shellfish immunology, 2019. **89**: p. 687-700.
51. Salinas, I., et al., *Dietary administration of Lactobacillus delbrüeckii and Bacillus subtilis, single or combined, on gilthead seabream cellular innate immune responses.* Fish & Shellfish Immunology, 2005. **19**(1): p. 67-77.
52. Cerezuela, R., et al., *Effects of dietary Bacillus subtilis, Tetraselmis chuii, and Phaeodactylum tricornutum, singularly or in combination, on the immune response and disease resistance of sea bream (Sparus aurata L.).* Fish & Shellfish Immunology, 2012. **33**(2): p. 342-349.
53. Mohammadian, T., et al., *Synbiotic effects of β-glucan, mannan oligosaccharide and Lactobacillus casei on growth performance, intestine enzymes activities, immune-hematological parameters and immune-related gene expression in common carp, Cyprinus carpio: An experimental infection with Aeromonas hydrophila.* Aquaculture, 2019. **511**: p. 634197.
54. Issimov, A., et al., *Prevalence of Clostridium perfringens and Detection of Its Toxins in Meat Products in Selected Areas of West Kazakhstan.* Agriculture-Basel, 2022. **12**(9).
55. Hernandez, L.H.H., et al., *Effects of the commercial probiotic Lactobacillus casei on the growth, protein content of skin mucus and stress resistance of juveniles of the Porthole livebearer Poecilopsis gracilis (Poecilidae).* Aquaculture Nutrition, 2010. **16**(4): p. 407-411.
56. Timmerman, H.M., et al., *Monostrain, multistrain and multispecies probiotics—A comparison of functionality and efficacy.* International Journal of Food Microbiology, 2004. **96**(3): p. 219-233.
57. Wang, Y., et al., *Probiotic Potential of Leuconostoc pseudomesenteroides and Lactobacillus Strains Isolated From Yaks.* Frontiers in Microbiology, 2018. **9**.

Figure

Click here to access/download;Figure;Fig1.tif

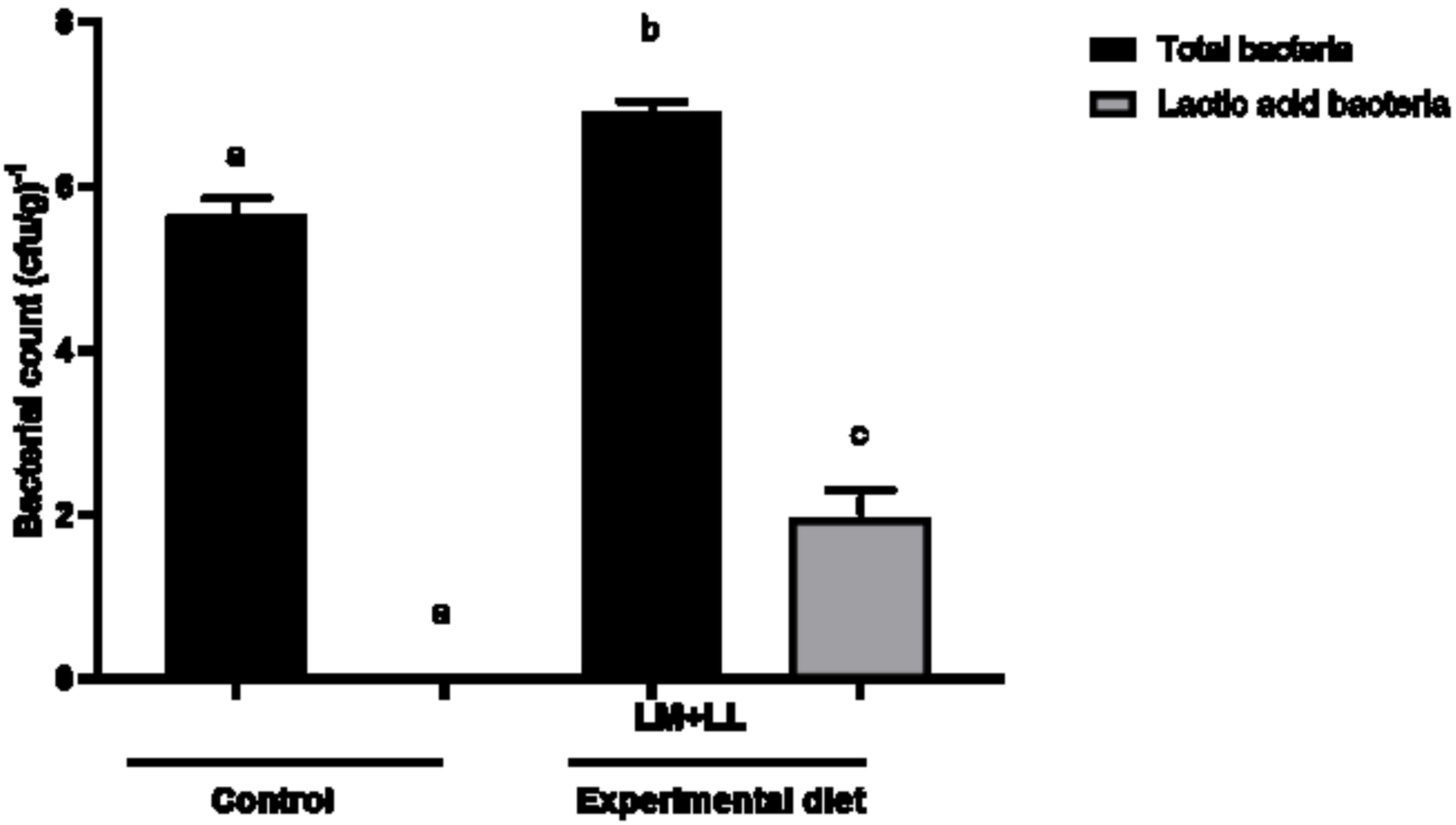

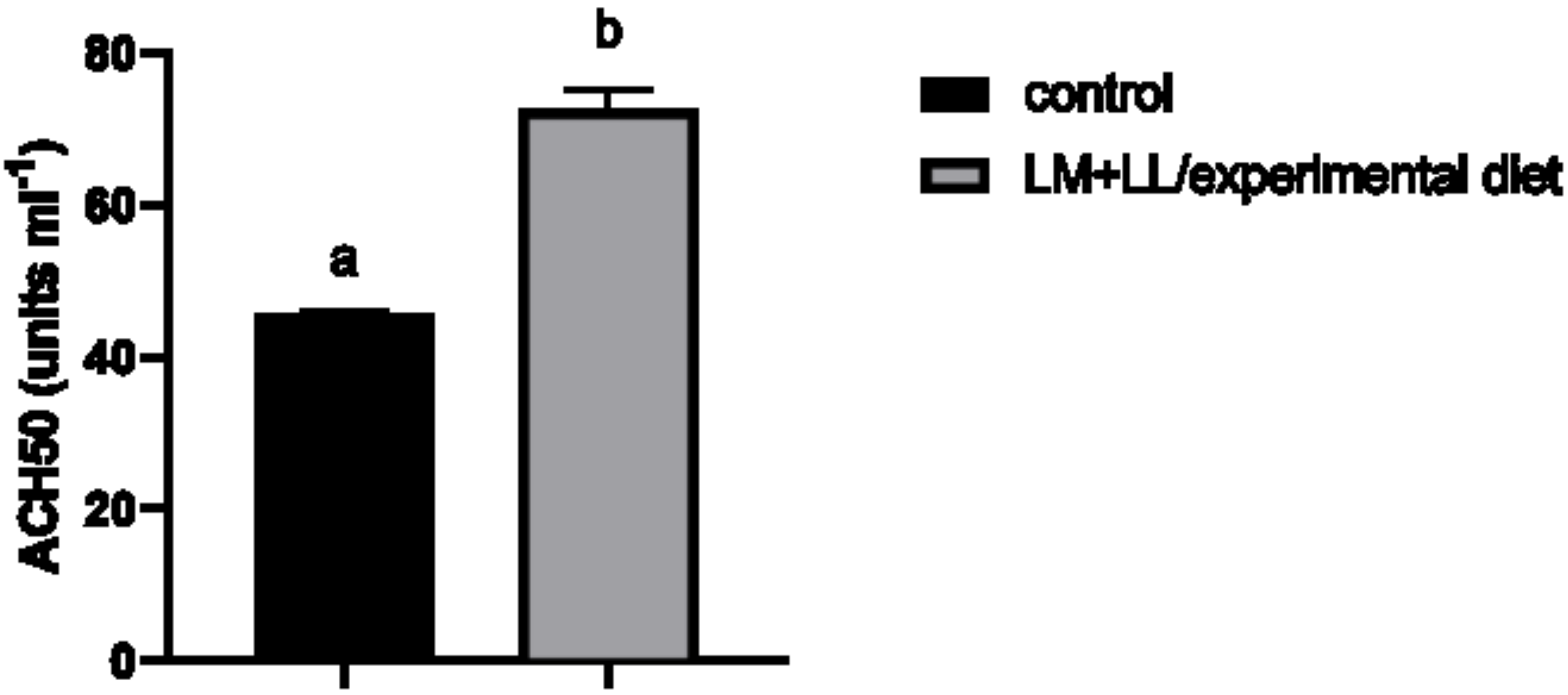
b
a
80
60
40
20
0
ACH50 (units ml^{-1})
control
LM+LL/experimental diet

Click here to access/download;Figure;Fig3.tif

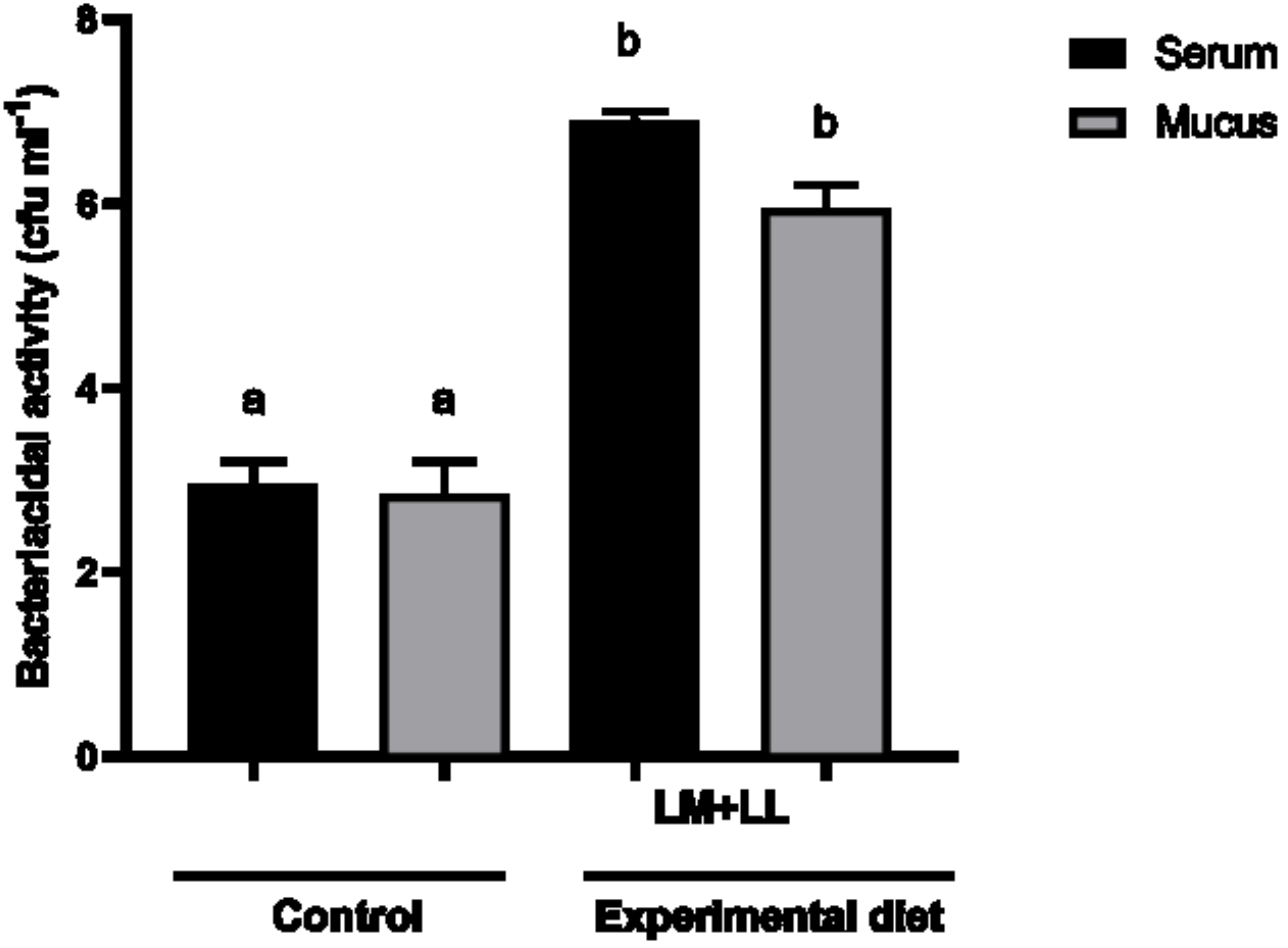

Figure
Click here to access/download;Figure;Fig4.tif

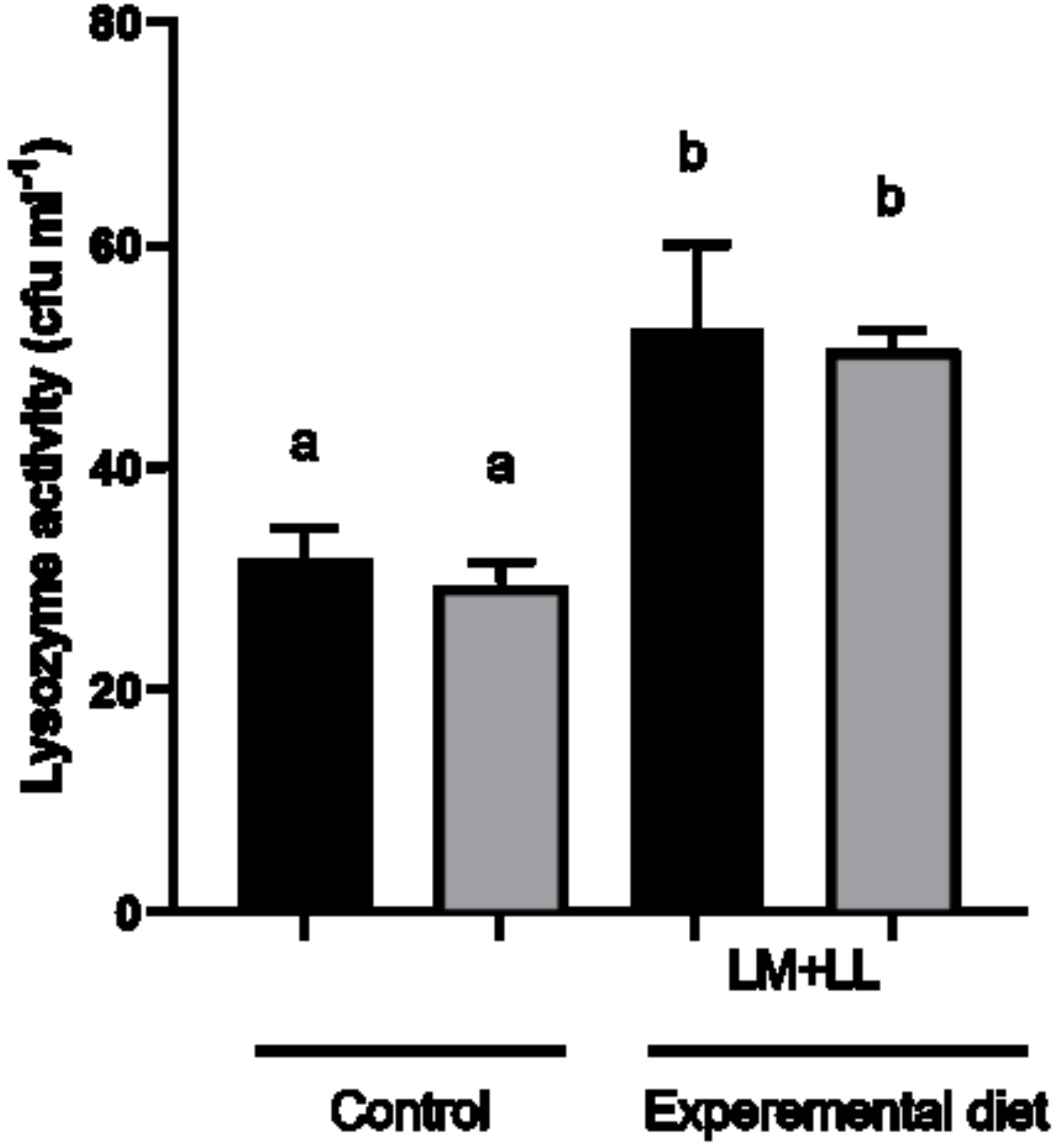

Click here to access/download;Figure;Fig5.tif

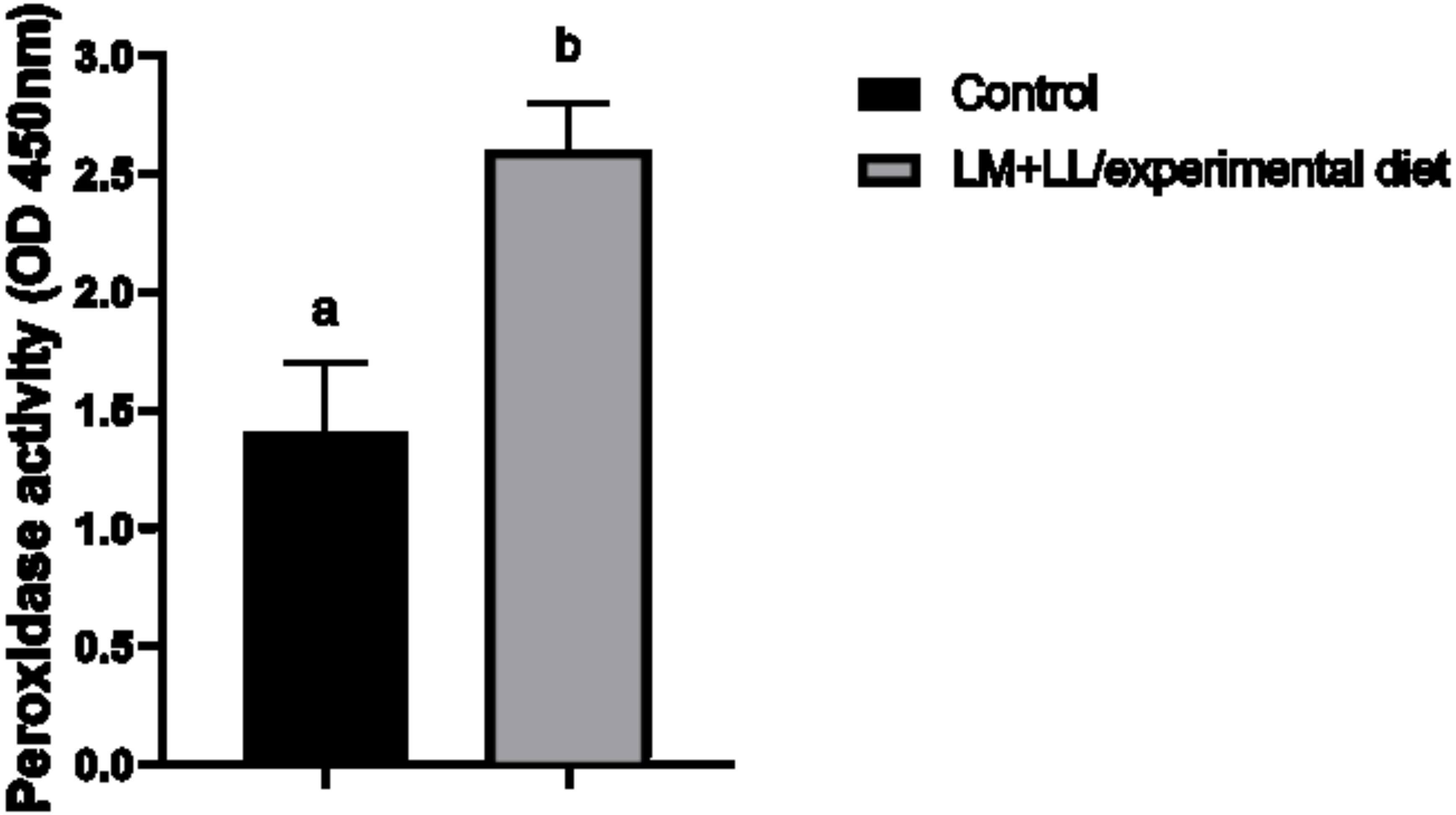

Click here to access/download;Figure;Fig6.tif

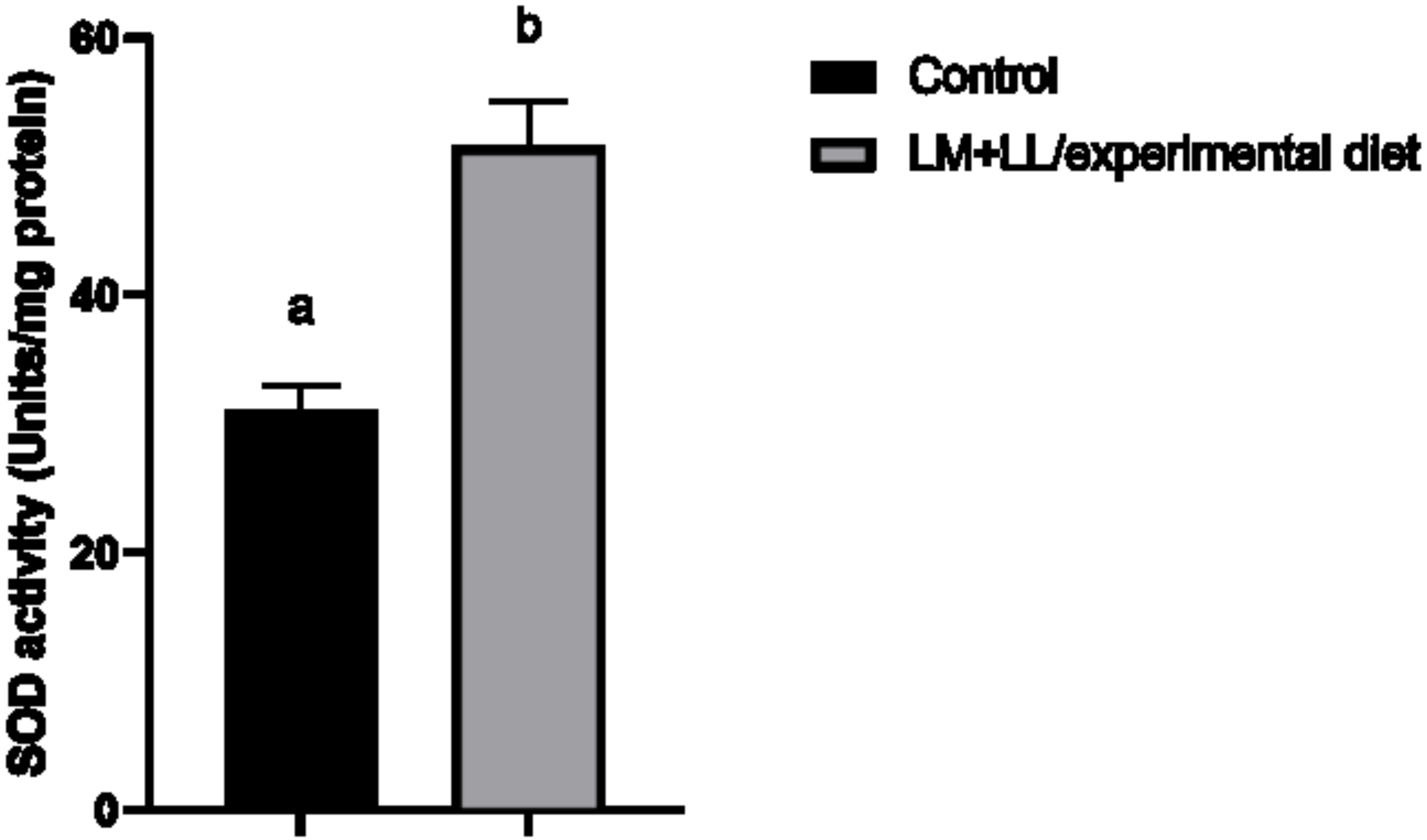

Click here to access/download;Figure;Fig7.tif

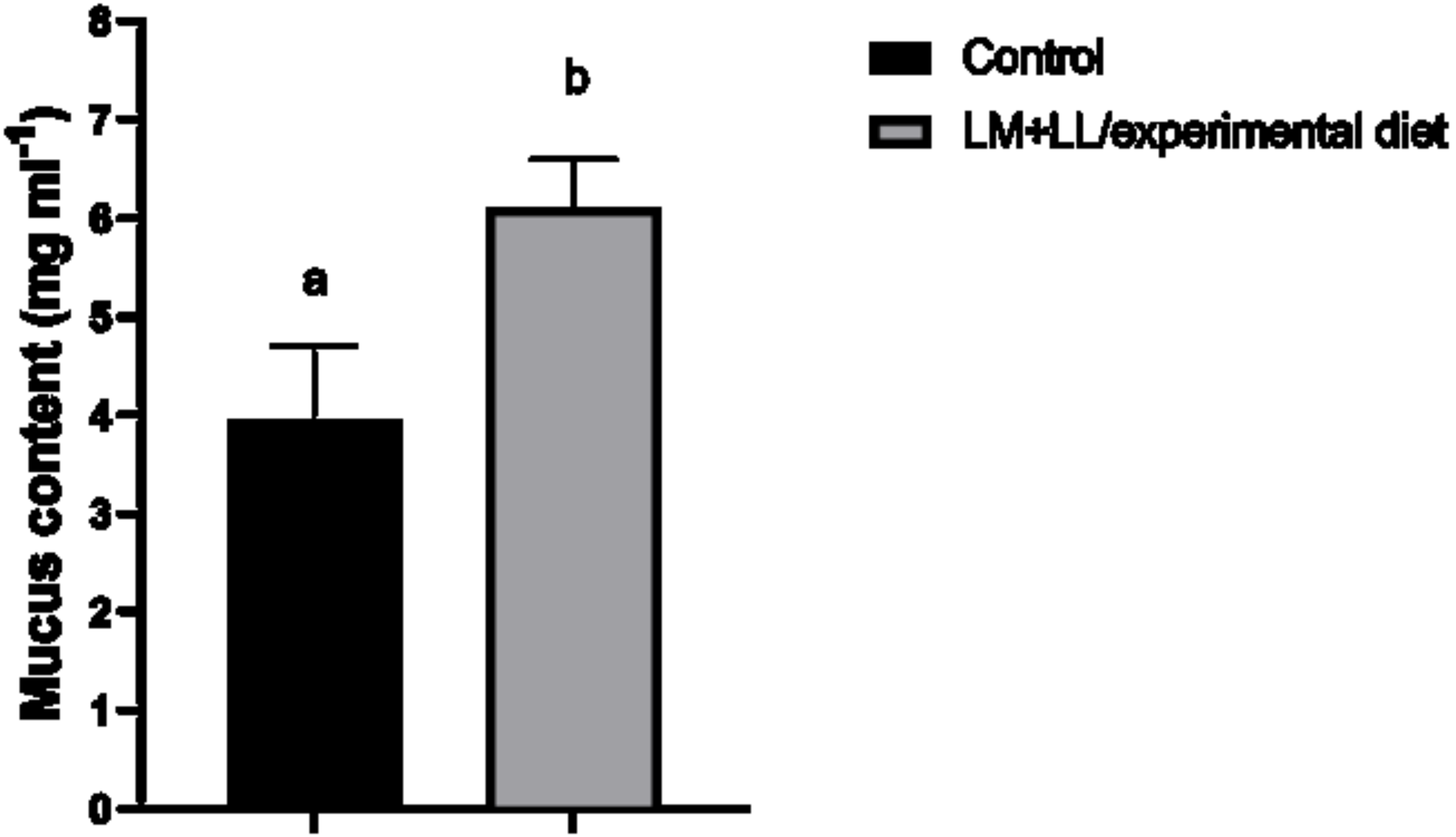

Supplement: Attachment — Submitted filename: PONE-D-24-34289-2.pdf [file pone.0312580.s002.pdf]
