# Supplementary material for: The dietary effects of two strain probiotics (Leuconostoc mesenteroides, Lactococcus lactis) on growth performance, immune response and gut microbiota in Nile tilapia (Oreochromis niloticus)
Source: PLoS One. 2024 Oct 24;19(10):e0312580. doi: 10.1371/journal.pone.0312580 (PMC11500904; doi:10.1371/journal.pone.0312580)
Supplement: Attachment — Submitted filename: PONE-D-24-34289_R1_reviewer.pdf [file pone.0312580.s004.pdf]

PLOS ONE

The dietary effects of two strain probiotics (Leuconostoc mesenteroides, Lactococcus lactis) on growth performance, immune response and gut microbiota in Nile tilapia (Oreochromis niloticus)

--Manuscript Draft--

**Manuscript Number:**	PONE-D-24-34289R1
**Article Type:**	Research Article
**Full Title:**	The dietary effects of two strain probiotics (Leuconostoc mesenteroides, Lactococcus lactis) on growth performance, immune response and gut microbiota in Nile tilapia (Oreochromis niloticus)
**Short Title:**	The dietary effect of multistrain probiotics
**Corresponding Author:**	Svetlana Bayantassova<br>Zhengir Khan West-Kazakhstan Agrarian Technical University<br>Oral, KAZAKHSTAN
**Keywords:**	Nile tilapia; probiotic; immunity; growth.
**Abstract:**	The aquaculture industry has been growing rapidly over the past few decades to meet future animal protein demands. However, intensive aquaculture industry faces challenges such as growth abnormalities, high mortality rates, water quality and intestinal health deterioration. Administering probiotics can serve as a nutritional strategy to enhance the immune system and growth performance of fish influxes of gut microbiota. This study aimed to evaluate the impact of two dietary probiotic strains L. mesenteroides and L. lactis on the growth performance, immunity, and gut microbiota of Nile tilapia (Oreochromis niloticus). Fish were fed with basal and experimental diet supplemented by both L. mesenteroides and L. lactis bacteria at 106 cell/g for 8 weeks. Feeding a combination of L. mesenteroides and L. lactis resulted in significant improvements in feed utilization parameters (PER and FER) (P < 0.001), alternative complement pathway activity , intestinal lactic acid bacteria count (P < 0.012), mucus secretion (P < 0.002) and peroxidase activity (P < 0.001) compared to the control groups. Serum lysozyme activity also exhibited a significant increase in the L. mesenteroides and L. lactis dietary group (P < 0.011) compared to the control and single probiotic supplemented diet groups. Furthermore, Nile tilapia fed the L. mesenteroides and L. lactis supplemented diet showed enhanced growth performance metrics (weight gain, final weight and specific growth rate) compared to those fed control and single probiotic supplemented diets (P < 0.022). Additionally, superoxide dismutase activity was significantly elevated in the L.mesenteroides and L. lactis supplemented diet groups compared to the control and single L.mesenteroides supplemented diet groups (P < 0.017). These findings strongly indicate that a dietary combination of L. mesenteroides and L. lactis probiotics could function as a beneficial immunostimulant feed supplement in Nile tilapia aquaculture.
**Order of Authors:**	Assel Paritova
	Akylbek Nurgaliyev
	Gulbaram Nurgaliyeva
	Nurzhan Abekeshev
	Altynay Abuova
	Faruza Zakirova
	Grzegorz Zwierzchowski
	Zhaxygali Kuanchaleyev
	Saltanat Issabekova
	Maigul Kizatova
	Zaure Sayakova

Powered by Editorial Manager® and ProduXion Manager® from Aries Systems Corporation

	Dinara Zhanabayeva
	Yelena Kukhar
	Ruslan Stozhkov
	Botagoz Aitkozhina
	Yevgeniy Mayer
	Svetlana Bayantassova
	Angsar Satbek
	Alexandr Andruchshak
	Kaissar Kushaliyev
**Response to Reviewers:**	Please, refer to attached files
**Additional Information:**	
**Question**	**Response**
**Financial Disclosure**<br><br>Enter a financial disclosure statement that describes the sources of funding for the work included in this submission. Review the submission guidelines for detailed requirements. View published research articles from *PLOS ONE* for specific examples.<br><br>This statement is required for submission and **will appear in the published article** if the submission is accepted. Please make sure it is accurate.<br><br>**Funded studies**<br>Enter a statement with the following details:<br>• Initials of the authors who received each award<br>• Grant numbers awarded to each author<br>• The full name of each funder<br>• URL of each funder website<br>• Did the sponsors or funders play any role in the study design, data collection and analysis, decision to publish, or preparation of the manuscript?<br><br>Did you receive funding for this work?	Yes
Please add funding details.<br>as follow-up to "**Financial Disclosure**<br><br>Enter a financial disclosure statement that describes the sources of funding for the	This research was funded by the Science Committee of the Ministry of Science and Higher Education of the Republic of Kazakhstan (grant no. AP19576848)

work included in this submission. Review the submission guidelines for detailed requirements. View published research articles from *PLOS ONE* for specific examples.<br><br>This statement is required for submission and **will appear in the published article** if the submission is accepted. Please make sure it is accurate.<br><br>**Funded studies**<br>Enter a statement with the following details:<br>• Initials of the authors who received each award<br>• Grant numbers awarded to each author<br>• The full name of each funder<br>• URL of each funder website<br>• Did the sponsors or funders play any role in the study design, data collection and analysis, decision to publish, or preparation of the manuscript?<br><br>Did you receive funding for this work?"	
Please select the country of your main research funder (please select carefully as in some cases this is used in fee calculation).<br>as follow-up to "**Financial Disclosure**<br><br>Enter a financial disclosure statement that describes the sources of funding for the work included in this submission. Review the submission guidelines for detailed requirements. View published research articles from *PLOS ONE* for specific examples.<br><br>This statement is required for submission and **will appear in the published article** if the submission is accepted. Please make sure it is accurate.	KAZAKHSTAN - KZ

Powered by Editorial Manager® and ProduXion Manager® from Aries Systems Corporation

**Funded studies**<br>Enter a statement with the following details:<br>• Initials of the authors who received each award<br>• Grant numbers awarded to each author<br>• The full name of each funder<br>• URL of each funder website<br>• Did the sponsors or funders play any role in the study design, data collection and analysis, decision to publish, or preparation of the manuscript?<br><br>Did you receive funding for this work?"	
**Competing Interests**<br><br>Use the instructions below to enter a competing interest statement for this submission. On behalf of all authors, disclose any competing interests that could be perceived to bias this work—acknowledging all financial support and any other relevant financial or non-financial competing interests.<br><br>This statement is required for submission and **will appear in the published article** if the submission is accepted. Please make sure it is accurate and that any funding sources listed in your Funding Information later in the submission form are also declared in your Financial Disclosure statement.<br><br>View published research articles from *PLOS ONE* for specific examples.	The authors have declared that no competing interests exist.

Powered by Editorial Manager® and ProduXion Manager® from Aries Systems Corporation

**NO authors have competing interests**<br><br>Enter: *The authors have declared that no competing interests exist.*<br><br>**Authors with competing interests**<br><br>Enter competing interest details beginning with this statement:<br><br>*I have read the journal's policy and the authors of this manuscript have the following competing interests: [insert competing interests here]*<br><br>* typeset	
**Ethics Statement**<br><br>Enter an ethics statement for this submission. This statement is required if the study involved:<br><br>• Human participants<br>• Human specimens or tissue<br>• Vertebrate animals or cephalopods<br>• Vertebrate embryos or tissues<br>• Field research<br><br>Write "N/A" if the submission does not require an ethics statement.<br><br>General guidance is provided below. Consult the submission guidelines for detailed instructions. **Make sure that all information entered here is included in the Methods section of the manuscript.**	The protocol was approved by the Committee on the Ethics of Animal Experiments of the Saken Seifullin Kazakh Agrotechnical University (permit number: 2).

Powered by Editorial Manager® and ProduXion Manager® from Aries Systems Corporation

**Format for specific study types**<br><br>**Human Subject Research (involving human participants and/or tissue)**<br>• Give the name of the institutional review board or ethics committee that approved the study<br>• Include the approval number and/or a statement indicating approval of this research<br>• Indicate the form of consent obtained (written/oral) or the reason that consent was not obtained (e.g. the data were analyzed anonymously)<br><br>**Animal Research (involving vertebrate animals, embryos or tissues)**<br>• Provide the name of the Institutional Animal Care and Use Committee (IACUC) or other relevant ethics board that reviewed the study protocol, and indicate whether they approved this research or granted a formal waiver of ethical approval<br>• Include an approval number if one was obtained<br>• If the study involved *non-human primates*, add *additional details* about animal welfare and steps taken to ameliorate suffering<br>• If anesthesia, euthanasia, or any kind of animal sacrifice is part of the study, include briefly which substances and/or methods were applied<br><br>**Field Research**<br><br>Include the following details if this study involves the collection of plant, animal, or other materials from a natural setting:<br>• Field permit number<br>• Name of the institution or relevant body that granted permission	
**Data Availability**<br><br>Authors are required to make all data underlying the findings described fully available, without restriction, and from the time of publication. PLOS allows rare exceptions to address legal and ethical concerns. See the PLOS Data Policy and FAQ for detailed information.	Yes - all data are fully available without restriction

Powered by Editorial Manager® and ProduXion Manager® from Aries Systems Corporation

A Data Availability Statement describing where the data can be found is required at submission. Your answers to this question constitute the Data Availability Statement and **will be published in the article**, if accepted.<br><br>**Important:** Stating 'data available on request from the author' is not sufficient. If your data are only available upon request, select 'No' for the first question and explain your exceptional situation in the text box.<br><br>Do the authors confirm that all data underlying the findings described in their manuscript are fully available without restriction?	
**Describe where the data may be found in full sentences. If you are copying our sample text, replace any instances of XXX with the appropriate details.**<br><br>• If the data are **held or will be held in a public repository**, include URLs, accession numbers or DOIs. If this information will only be available after acceptance, indicate this by ticking the box below. For example: *All XXX files are available from the XXX database (accession number(s) XXX, XXX.).*<br>• If the data are all contained **within the manuscript and/or Supporting Information files**, enter the following: *All relevant data are within the manuscript and its Supporting Information files.*<br>• If neither of these applies but you are able to provide **details of access elsewhere**, with or without limitations, please do so. For example:<br><br>*Data cannot be shared publicly because of [XXX]. Data are available from the XXX Institutional Data Access / Ethics Committee (contact via XXX) for researchers who meet the criteria for access to confidential data.*<br><br>*The data underlying the results presented in the study are available from (include the name of the third party*	The original data presented in the study are openly available in FigShare at 10.6084/m9.figshare.27050416 .

Powered by Editorial Manager® and ProduXion Manager® from Aries Systems Corporation

and contact information or URL).<br>• This text is appropriate if the data are owned by a third party and authors do not have permission to share the data.<br><br>* typeset	
Additional data availability information:	

Powered by Editorial Manager® and ProduXion Manager® from Aries Systems Corporation

Manuscript Click here to access/download;Manuscript;manuscript.docx

The dietary effects of two strain probiotics (*Leuconostoc mesenteroides, Lactococcus lactis*) on growth performance, immune response and gut microbiota in Nile tilapia (*Oreochromis niloticus*)

Assel Paritova[1], Akylbek Nurgaliyev[2], Gulbaram Nurgaliyeva[2], Nurzhan Abekeshev[2], Altynay Abuova[3], Faruza Zakirova[2], Grzegorz Zwierzchowski[4], Zhaxygali Kuanchaleyev[1], Saltanat Issabekova[1], Maigul Kizatova[5], Zaure Sayakova[6], Dinara Zhanabayeva[1], Yelena Kukhar[1], Ruslan Stozhkov[1], Botagoz Aitkozhina[1], Yevgeniy Mayer[1], Svetlana Bayantassova[2*], Angsar Satbek[1], Alexandr Andruchshak[1], Kaissar Kushaliyev[2*].

1. Saken Seifullin Kazakh Agrotechnical University, Astana, 010000, Kazakhstan.
2. Zhangir Khan West Kazakhstan Agrarian–Technical University, Uralsk 090000, Kazakhstan.
3. International Engineering and Technological University, Almaty, 050000, Kazakhstan.
4. University of Warmia and Mazury, Olsztyn, 10-719, Poland.
5. S.D. Asfendiyarov Kazakh National Medical University, 050000, Almaty, Kazakhstan.
6. Kazakh Scientific Veterinary Research Institute, 500016, Almaty, Kazakhstan.

*** Corresponding authors:** 1. Svetlana Bayantassova, bayantasova65@mail.ru

2. Kaissar Kushaliyev, kaissark@mail.ru

Abstract

The aquaculture industry has been growing rapidly over the past few decades to meet future animal protein demands. However, intensive aquaculture industry faces challenges such as growth abnormalities, high mortality rates, water quality and intestinal health deterioration. Administering probiotics can serve as a nutritional strategy to enhance the immune system and growth performance of fish influxes of gut microbiota. This study aimed to evaluate the impact of two dietary probiotic strains *L. mesenteroides* and *L. lactis* on the growth performance, immunity, and gut microbiota of Nile tilapia (*Oreochromis niloticus*). Fish were fed with basal and experimental diet supplemented by both *L. mesenteroides* and *L. lactis* bacteria at 10^6 cell/g

for 8 weeks. Feeding a combination of *L. mesenteroides* and *L. lactis* resulted in significant improvements in feed utilization parameters (PER and FER) ($P < 0.001$), alternative complement pathway activity , intestinal lactic acid bacteria count ($P < 0.012$), mucus secretion ($P < 0.002$) and peroxidase activity ($P < 0.001$) compared to the control groups. Serum lysozyme activity also exhibited a significant increase in the *L. mesenteroides* and *L. lactis* dietary group ($P < 0.011$) compared to the control and single probiotic supplemented diet groups. Furthermore, Nile tilapia fed the *L. mesenteroides* and *L. lactis* supplemented diet showed enhanced growth performance metrics (weight gain, final weight and specific growth rate) compared to those fed control and single probiotic supplemented diets ($P < 0.022$). Additionally, superoxide dismutase activity was significantly elevated in the *L.mesenteroides* and *L. lactis* supplemented diet groups compared to the control and single *L.mesenteroides* supplemented diet groups ($P < 0.017$). These findings strongly indicate that a dietary combination of *L. mesenteroides* and *L. lactis* probiotics could function as a beneficial immunostimulant feed supplement in Nile tilapia aquaculture.

Keywords: Nile tilapia, probiotic, immunity, growth.

Introduction

Over the past few decades, there has been considerable growth in the aquaculture industry, positioning it to effectively address the future animal protein demands of the human population [1]. Tilapia ranks among the most extensively farmed fish globally, representing one of the most widely practiced forms of aquaculture. They are highly suitable for aquaculture due to their ease of cultivation, rapid growth rates, and relatively high tolerance to environmental stressors compared to other fish species [2]. Nonetheless, intensive aquaculture encounters challenges that have raised concerns regarding fish health, encompassing growth abnormalities, mortality rates, and disease outbreaks [3]. Consequently, farmers are forced to use prophylactic antibiotics to mitigate these challenges [4, 5].

Antibiotics are commonly employed in aquaculture to manage diseases and enhance growth performance. However, their usage in this context has the potential to disrupt intestinal

microbiota and foster the emergence of antibiotic-resistant bacteria, posing risks to aquatic life [5, 6]. Consequently, previous studies aimed to devise alternative feed additives to substitute antibiotics in aquaculture practices [7-10]. The application of probiotics as dietary additives has been recently proposed as a viable alternative to antibiotics [11, 12]. Their application aims to reduce the colonization gastrointestinal mucosa by pathogenic population, thereby enhancing the health of mammals and fish [13-15]. Additionally, lactic acid bacteria (LAB) were ability to increase growth performance and survival rates, prevent intestinal disorders such as diarrhea, neutralize antinutritional factors [16, 17]. Moreover, advantageous intestinal microbiota can produce enzymes that have the potential to enhance the digestibility and uptake of nutritional components [18]. It was documented that incorporating a combination of probiotics into the diet of *Labeo rohita* fingerlings resulted in improved nutrient digestibility, enhanced growth, a higher protein efficiency ratio, and a reduced feed conversion ratio compared to the use of individual strains [19]. In this study, we have used two probiotics, L. mesenteroides and L. lactis, included in the diet of Nile tilapia (*Oreochromis niloticus*). These LAB were isolated from the gastrointestinal tracts of different fish species (e.g. northern pike, zebrafish, carp,). They are important in aquaculture as they help balance the intestinal microbiota, enhance growth performance, improve digestion and nutrient absorption, and boost the immune response

This study examined the effect of two probiotic strains in fresh water fish Nile tilapia on growth performance, cultivable gut bacteria, and innate immune response, as well as body weight gain.

Materials and Methods

Animal Ethics

The experiment was carried out according to the European Convention for the Protection of Vertebrate Animals used for Experimental and Other Scientific Purposes guidelines [20]. The protocol was approved by the Committee on the Ethics of Animal Experiments of the Saken Seifullin Kazakh Agrotechnical University (permit number: 2).

Experimental design

Overall, 395 healthy juvenile Nile tilapia with body weight averaging 45±2g were purchased from local commercial fish farm. Fish were then equally divided in four groups in 500L tank: control group without probiotic supplementation (C) 96 heads and three experimental groups *L. mesenteroides (LM), L. lactis (LL) and a combination of both (mix)* – each consisting of 96 fish per group. Subsequently, the fish were randomly distributed into twelve 100-liter tanks, with thirty-two fish per tank and two replicate tanks per treatment. Each tank was fitted with both an inlet and an outlet and maintained in a flow-through freshwater system. Prior experiment onset, fish allowed to acclimatize in 500L tank with 5mg/L O_2 aerated water for 14 days. Water quality parameters were assessed by employing a thermometer to measure temperature and an oxygen meter (Model FEBO 330, EPF Instruments, Fisher Scientific, USA) to measure dissolved oxygen levels. Throughout the experiment, water temperature was maintained at 27°C. The pH level was monitored with a portable pH meter (Fisherbrand AP 115). During the acclimatization period fish were fed a control diet without probiotic supplements *ad libitum* twice a day at 9:00 a.m. and 17:00 p.m. After completing the feeding trial (8 weeks), all experimental fish subjected a 24-h fasting. Following this, survival rate, individual body weight, immune parameters, and gut microbiota composition of fish from each tank were determined.

Isolation of Probiotics

L. mesenteroides and *L. lactis* LAB starins were isolated from intestines of common carp (*Cyprinus carpio*) fingerlings following protocol described by [3]. In brief, a 50 µl sample of intestinal microflora from fish was dissolved in 500 µl distilled water (1:10 ratio). Subsequently, using a disposable pipette, 100 µl of the suspension was introduced to the MRS agar medium and incubated at 37°C for 48-h. Glass beads were utilized to ensure uniform growth of isolated colonies. Eight to twelve glass beads (pre-sterilized) were placed into the Petri dish containing the inoculum. The probiotics obtained were washed three times in distilled water and plates were sent for genomic confirmation.

PCR

For DNA extraction, GeneJET PCR Purification Kit (Thermo Fisher Scientific, USA) was used according to the manufacturer's instructions. Following reference strains were utilized as a positive controls: National Collection of Type Culture (NCTC) AB023246 for *L. mesenteroides* and (NCTC) M58837 for *L. lactis*. For genotyping of isolates, multiplex PCR (mPCR) was run according to the protocol described by Ali, Kot [21]. The amplification of 16S mDNA fragments in a Thermal Cycler (Eppendorf Mastercycler) were as follows: 95◦C for 2 min 30 s, 95◦C for 1 min, 55◦C for 1 min, 72◦C for 1 min 20 s (35 cycles), and 72◦C for 2 min. The PCR products obtained were administered in 1% agarose gel electrophoresis in the presence of Ethidium bromide, and the results were visualized using Bio-Imaging Systems (MiniBIS Pro, Israel).

Preparation of Experimental Diets

Commercial feed (F.lli Fragola, S.P.A, Italy) without probiotic supplements was used as a basal control diets. Basal diet composed of fishmeal, soybean meal, milled wheat, extruded peas, potato starch, vegetable oil, tricalcium phosphate, premix, yeast (Table 1).

The culture medium was prepared using a water-based solution with the addition of up to 1.5% glucose. Activated strains of *L. lactis* and *L. mesenteroides* were used as inoculants, added at a concentration of 0.05%. Briefly, in distilled water, the following components were added: 1.5% glucose, 0.1% buffer salts, 0.1% ascorbic acid, 5% peptone, 1.5% microbiological agar, and 0.5% yeast. The pH of the medium was adjusted to (7.0±0.1). The prepared medium was sterilized at 121°C for 30 minutes, then cooled to (37±1)°C. Then, an inoculum containing the activated strains *L. lactis* and *L. mesenteroides* at a concentration of 0.05% was introduced. Biomass cultivation was carried out under batch fermentation for (24±2) hours, with pH neutralization using Na_2 CO_3 at the 12-hour mark. After cultivation, the upper layer of the culture broth was separated, and the bacterial suspension was cooled to (4±2)°C, dispensed into aseptic vials (10-12 ml), sealed, and labeled. The experimental diet was prepared by introducing 1×10^7 CFU/g of a single *L. mesenteroides* or/and *L. lactis* and mixed probiotic in the basal diet.

To do so, these LAB were incorporated into the basal diet using the spraying method while stirring. Then, 165g of gelatin was dissolved in 500 ml of distilled water to create a waterproof membrane for the feed. Next, the dietary components underwent grinding into a fine powder, followed by thorough agitation and subsequent cold extrusion into pellets 0.5 mm in diameter. Finally, the diluted gelatin was sprayed onto LAB incorporated basal diet pellets under thorough stirring. The complete diet were then air-dried overnight at room temperature and stored in plastic containers at −4°C until required.

Table 1. Composition and nutrient levels of the basal diet (g kg-1)

**Ingredients**	**Diet**
Fishmeal	30
Soybean meal	42
Milled wheat	7
Extruded peas	8
Potato starch	6.7
Vegetable oil	4
Vitamin/Mineral mixture	1.1
Premix	0.1
Yeast	0.05
Gelatin	1
**Proximate composition**	
Moisture	8.79
Crude protein	35.8
Crude lipid	7.39
Crude ash	6.44

Growth performance

The survival rate was calculated according to following equation: Survival (%) = 100 × (final number of fish/initial number of fish). The calculation of weight gain (WG), feed conversion ratio (FCR), protein efficiency ratio (PER) and specific growth rate (SGR) were performed according to protocol described by Allameh, Yusoff [22]. In brief, WG in grams is calculated by

subtracting the initial weight from the final weight. SGR is determined by the formula: SGR = 100 (ln W2 - ln W1) / T, where W1 and W2 represent the initial and final weights, respectively, and T signify the number of days during the feeding period. SGR indicates the percentage of growth per day for each fish.FCR is computed as the ratio of feed intake in grams to weight gain in grams, while PER is determined by the ratio of weight gain in grams to protein intake in grams.

Determination of Intestinal bacteria activities

To determine the total intestinal microbiota and LAB count, eleven fish were sampled from each study group following 24-h feeding cessation. Prior experiment onset, fish were anesthetized using tricaine methanesulphonate (MS 222) and then transferred on to sterile stainless desk. Before opening, fish carcasses were rinsed with 70% ethanol to eliminate surface contamination. The whole intestine was extracted, and 1 cm sections from both the anterior and posterior ends were discarded. Then the whole intestinal tract was dissected, and rinsed three times in PBS. Subsequently, tenfold serial dilution was run in PBS. 100 ml aliquots of the diluted solution were then plated onto duplicate Trypto-Soya agar (TSA) media to determine total bacterial counts. To isolate viable LAB species, 100 ml of each dilution was introduced in MRS (DeMan, Rogosa, and Sharpe) agar and allowed to incubate at 25°C for 3 to 5 days. Colony-forming units of viable bacterial populations were enumerated according to protocol reported by Ramos, Batista [23].

Immune responses

Alternative complement pathway activity (ACH50) was determined according to protocol of Yano [24]. In brief, 0.1ml aliquots of diluted serum were transferred in to plastic tubes and adjusted to a total volume of 0.25 ml using barbitone buffer. Rabbit red blood cells (RaRBC) were then added to each tube in a volume of 0.1 ml and allowed to incubate for 1-h at 20°C. Next, after incubation, 3.15 ml of 0.9% NaCl solution was introduced and samples were centrifugated at 1600 x g for 10 min at 4°C to remove RaRBC derbies. Finally, obtained

supernatant was measured for optical density at 414 nm, and a lysis curve was generated. The volume resulting in 50% hemolysis was identified and utilized to assess the complement activity of the sample.

The bactericidal activity of serum and mucus was assessed following the method described by Dawood, Koshio [25]. To do so, samples were diluted in 1:1 ratio with Tris buffer adjusted to pH 7.5. Then, diluted mixture was agitated with pre-prepared 0.001 mg/ml *Escherichia coli*, IAM1239 bacterial suspension (RIBSP, Kazakhstan) and allowed to incubate overnight at room temperature. The standard plate counting method was employed to calculate colony-forming units (CFU).

To measure lysozyme activity in serum and mucus samples a turbidimetric assay was utilized. Briefly, 10 µl of samples were mixed with 190 µl of *Micrococcus lysodeikticus* bacterium (0.2 mg/PBS adjusted to pH 7.4) in 96 flat-bottomed well plates, and the mixture was allowed to incubate for 5 min at room temperature. Absorbance was measured at 450 nm using Biotec 800 TS (Agilent Technologies, USA). Lysosome activity was determined based on the amount of sample resulting in a decrease in optical density of 0.001 min^{-1}.

The total peroxidase content in serum was assessed according to method described by Salinas, Abelli [26]. To do this, 15 µl of serum and 35 µl of Hank's Buffered Salt Solution were added to 96 flat-bottomed well plates. Subsequently, 50 µl of 3,3′,5,5′-Tetramethylbenzidine (TMB, Thermo Scientific, USA) was added to each well. The diluted serum was then incubated for 20 min, and the color-developing reaction was halted by introducing 50 µl of 2 M sulfuric acid. The optical density was measured at a wavelength of 450 nm using a plate reader.

Serum superoxide dismutase (SOD) activity was quantified by the percentage inhibition rate of the enzyme's reaction using Ransod kit (Randox, Crumlin, UK) following the protocol described by Sun, Yang [27]. The specific SOD activity units were adjusted by milligrams of protein (mg $protein)^{-1}$. One unit of SOD is determined as the quantity of the enzyme present in 20 ml of the

sample solution, which inhibits the reduction reaction of nitroblue tetrazolium (NBT) by 50% at wavelength of 550 nm.

Statistical analysis

Graphs were compiled and statistical analyses were conducted to protocol described by Bayantassova, Kushaliyev [28] using Prism 9 software (GraphPad Software, Inc., San Diego, CA). The data were subjected to Kolmogorov–Smirnov and Levene's tests to assess the normality of the distribution and verify the homogeneity of variances. Data were analysed by one-way ANOVA with Tukey's post hoc test. Statistical significance between groups was denoted when $P < 0.05$ using asterisks, as detailed in the respective figures.

Results

Demonstration of growth performance, nutrient utilization and survival.

The growth parameters, feed uptake and survival of control and fish fed *L. mesenteroides* or/and *L. lactis* diet are presented in Table 2. Fish fed the mixture of *L. mesenteroides* and *L. lactis* diet demonstrated a substantial increase in WG, FBW, and SGR ($P < 0.033$) compared to the groups fed single *L. mesenteroides* or the control diets. Additionally, the PER and FER profiles were significantly higher in fish fed on mixture of *L. mesenteroides* and *L. lactis* diet ($P < 0.001$), whereas these profiles did not differ between single probiotic supplemented groups ($P > 0.991$) Moreover, FER profile did not differ significantly between fish fed single probiotic supplemented diets and those fed control diet. Furthermore, a significant increase in the protein gain (PG) profile was observed in fish fed *L. mesenteroides* or/and *L. lactis*-containing diet compared to the control group ($P < 0.021$). Feed intake profile was enhanced by both *L. mesenteroides* and *L. lactis* dietary treatments, with fish receiving only *L. lactis* exhibiting significantly greater feed intake compared to the control group ($P < 0.041$). However, no significant difference was observed between fish fed single probiotic supplemented diets ($P > 0.087$). Survival rates were high across all treatment groups, and no mortality was observed.

Table 2. Growth performance of Nile tilapia fed with *L. mesenteroides* or/and *L. lactis* supplemented diets for 8 weeks.

	Diets			
	Control	*L. mesenteroides*	*L. lactis* probiotics	Mixture of *L. mesenteroides* and *L. lactis* probiotics
[1]IBW (g)	46.3±1.32	45.4±3.44	45.9±1.02	45.8±2.11
[2]FBW (g)	68.4±3.26^{a}	79.2±1.16^{b}	78.5±2.15^{b}	92.4±4.54^{c}
[3]WG (%)	22.4±3.01^{a}	31.7±1.52^{b}	31.2±1.74^{b}	47.3±2.02^{c}
[4]SGR (%)	1.96±0.11^{a}	2.32±0.5^{b}	2.30±0.3^{b}	2.82±0.2^{c}
[5]FI (g)	12.42±0.47^{a}	12.88±1.05^{ab}	13.01±0.6^{b}	13.24±0.8^{b}
[6]FER	2.33±0.2^{a}	2.64±0.8^{ab}	2.60±1.12^{ab}	2.97±0.5^{c}
[7]PER	3.05±0.4^{a}	3.44±0.18^{b}	3.52±0.7^{b}	3.65±0.03^{c}
[8]PG	141.23±7.2^{a}	158.42±2.13^{b}	162.32±3.04^{b}	179.31±4.05^{c}

[1]IBW - initial body weight; [2]FBW - final body weight; [3]WG - weight gain; [4]SGR - specific growth rate; [5]FI – feed intake; [6]FER - feed efficiency ratio; [7]PER - protein efficiency ratio; [8]PG - protein gain. Values represent means ± SEM of triplicate groups. Different superscripts in the line represent significant difference $p \leq 0.05$.

Microbiological analyses of gut microbiota

Both the control and experimental groups showed no detectable LAB bacteria in the intestines before the experiment. Fish fed with single and the mixture of LAB-supplemented diets demonstrated significantly greater levels of the total intestinal microbiota compared to the control group. ($P < 0.012$) (Figure 1). Moreover, fish fed with the mixture of LAB-supplemented diets exhibited significantly higher cultivable LAB compared to the fish groups fed with single diets ($P < 0.027$) (Figure 1).

Figure 1. Comparison of total bacteria and LAB content of Nile tilapia intestines fed with control and probiotics supplemented diets for 8 weeks. Data presented as the mean ±SEM from two combined independent experiments (n=10 fish/group). Bars with different alphabet are statistically different ($P \leq 0.05$). Bars with the same alphabet are statistically insignificant different ($P \geq 0.05$). * LM; LL – *L. mesenteroides*; *L. lactis*.

Demonstration of Immune responses

The rate of ACP were significantly elevated in fish fed *L. mesenteroides* or/and *L. lactis* supplemented diets than that of control group (Figure 2). Likewise, serum and mucus bactericidal activity (Figure 3), mucus lysozyme activity (Figure 4), peroxidase activity (Figure 5) demonstrated a considerable elevation in fish fed with single or mixture of probiotics supplemented diets compared to control group. Similarly, superoxide dismutase inhibition rate was exhibited significantly greater in fish groups fed with single *L. lactis* and mixture diets compared to other groups (Figure 6). Moreover, peroxidase activity (Figure 5) and ACP levels (Figure 2) in fish fed the combined L. mesenteroides and L. lactis diet were significantly elevated compared to those in fish fed single probiotic supplemented diets.

Additionally, when compared with the control and single probiotic supplemented diet groups, lysozyme activities of serum was significantly higher in fish fed mixture of *L. mesenteroides* and *L. lactis* diets ($P < 0.011$; Figure 4). Furthermore, the amounts of secreted mucus was significantly higher in fish groups supplemented with *L. mesenteroides* or/and *L. lactis* diets compared to control group ($P < 0.002$; Figure 7).

Figure 2. Alternative complement pathway of Nile tilapia fed with control and probiotics supplemented diets. Data presented as the mean ±SEM from two combined independent experiments (n=10 fish/group). Bars with different alphabet are statistically different ($P \leq 0.05$). Bars with the same alphabet are statistically insignificant different ($P \geq 0.05$). * LM; LL – *L. mesenteroides*; *L. lactis*.

Figure 3. Serum and mucus bactericidal activity of Nile tilapia fed with control and probiotics supplemented diets. Data presented as the mean ±SEM from two combined independent experiments (n=10 fish/group). Bars with different alphabet are statistically different ($P \leq 0.05$). Bars with the same alphabet are statistically insignificant different ($P \geq 0.05$).

* LM; LL - *L. mesenteroides*; *L. lactis*.

Figure 4. Serum and mucus lysozyme activity of Nile tilapia fed with control and probiotics supplemented diets. Data presented as the mean ±SEM from two combined independent experiments (n=10 fish/group). Bars with different alphabet are statistically different ($P \leq 0.05$). Bars with the same alphabet are statistically insignificant different ($P \geq 0.05$). * LM; LL - *L. mesenteroides*; *L. lactis*.

Figure 5. Serum peroxidase activity of Nile tilapia fed with control and probiotics supplemented diets. Data presented as the mean ±SEM from two combined independent experiments (n=10 fish/group). Bars with different alphabet are statistically different ($P \leq 0.05$). Bars with the same alphabet are statistically insignificant different ($P \geq 0.05$). * LM; LL - *L. mesenteroides*; *L. lactis*.

Figure 6. Superoxide dismutase activity of Nile tilapia fed with control and probiotics supplemented diets. Data presented as the mean ±SEM from two combined independent experiments (n=10 fish/group). Bars with different alphabet are statistically different ($P \leq 0.05$). Bars with the same alphabet are statistically insignificant different ($P \geq 0.05$). * LM; LL - - *L. mesenteroides*; *L. lactis*.

Figure 7. The effect of probiotics supplemented diets on mucus secretion. Data presented as the mean ±SEM from two combined independent experiments (n=10 fish/group). Bars with different alphabet are statistically different ($P \leq 0.05$). Bars with the same alphabet are statistically insignificant different ($P \geq 0.05$). * LM; LL - - *L. mesenteroides*; *L. lactis*.

Discussion

An increasing body of research has illustrated the application of single lactic acid bacteria species as a probiotic treatment to enhance growth performance, immune response, feed utilization and gut microbiota modulation in fish [29-31]. However, there has been limited focus on exploring the potential effects that may arise from the concurrent oral administration of multiple LAB species to fish [3, 32-34]. Hence, this study was carried out to assess the dietary effect of *L. mesenteroides* and *L. lactis* on the growth performance, immunity and gut microbiota of Nile tilapia.

The findings of this study suggest that administering both *L. mesenteroides* and *L. lactis* into supplementation diet significantly enhances the growth performance of Nile tilapia compared to the control group over a 8 weeks period. This outcome is consistent with previous research demonstrating that probiotic supplementation, particularly with *Lactobacillus* species, improves the growth performance of various fish species, including olive flounder (*P. olivaceus*) [33], Nile tilapia (*Oreochromis sp*) [35], gilthead sea bream (*Sparus aurata*) [36]. Furthermore, *L. lactis* has been found to enhance the growth performance of olive flounder [37] and grouper (*Epinephelus coioides*) [38]. Additionally, the results obtained in this study showed that fish fed a mixture of *L. mesenteroides* and *L. lactis* exhibited higher final weight, weight gain, and specific growth rate compared to the groups fed the control and single probiotic diets. Similarly, Allameh, Yusoff [22] reported that administering multistrain probiotics, particularly *L. mesenteroides*, enhanced the growth performance of Javanese carp (*Puntius gonionotus*). In the current study, fish fed the diet supplemented with a mixture of *L. mesenteroides* and *L. lactis* showed significant improvements in feed intake and feed utilization parameters. This observation suggests that the probiotic supplement may have influenced the palatability of the feed, consequently resulting in slight increases in the growth rate and feed intake of fish. To the best of our knowledge, this study represents the first dietary incorporation of *L. mesenteroides* and *L. lactis* leading to enhanced growth and feed utilization in Nile tilapia. Xia, Cao [39]

proposed that probiotics primarily influence Nile tilapia by augmenting host nutrition, thereby stimulating digestive enzymes and ultimately leading to enhanced growth and feed efficiency ratios. Moreover, the colonization of probiotic microorganisms in the intestine enhances microbial equilibrium, subsequently improving feed utilization and nutrient absorption [40-42]. Improvements in growth performance and feed utilization may also be attributed to enhanced intestinal digestive functions in fish owing to probiotic supplementation (*L. mesenteroides and L. lactis*), which likely includes increased activities of digestive enzymes. Previous studies have shown that supplementation with *Lactobacillus* species leads to improved nutrient digestibility [9]. It is well-established that the activity of digestive enzymes is positively correlated with the digestive capacity of fish [43], thereby enhancing their ability to extract nutrients from food [44]. It has been reported that digestive organs are highly sensitive to food composition, leading to immediate changes in the activities of digestive enzymes ultimately affecting fish growth and health [45]., Furthermore, bacteria secrete proteases to cleave peptide bonds in proteins, breaking them down into free amino acids and constituent monomers, thereby improving the animal's nutritional status. Bacterial enzymatic hydrolysis has been demonstrated to improve the bioavailability of lipids, protein and dry matter [46], potentially leading to the increased growth and nutrient utilization observed in this study.

The current study revealed a significant enhancement in both total intestinal bacteria and lactic acid bacteria counts, further supporting the improvements observed in growth and feed utilization. Similar to the findings of Mohapatra, Chakraborty [19], a substantial increase in total bacterial counts was noted when fish were fed diets supplemented with probiotics. This increase could potentially lead to improved fish health and immunity, thereby promoting growth. It has been documented that the colonization rate of bacteria in the digestive tract is directly influenced by the level of dietary bacteria intake [47]. In our investigation, the elevated adherence of microorganisms introduced via dietary supplementation could explain the improved nutrient

utilization and growth observed in the fish. Numerous studies have suggested that probiotics function by colonizing the host and secreting various growth-promoting compounds [48] [49]. Previous studies have documented the crucial interplay between intestinal epithelial cells and probiotics in regulating the mucosal immunity of the fish gut. This interaction modulates both the physical and immunological barrier functions of the intestine and stimulates an immune response [50-54].

The results of the current investigation also indicate that the dietary combination of both *L. mesenteroides* and *L. lactis* led to an enhanced immune response. These improvements included increased lysozyme and bactericidal activities in both serum and mucus, as well as enhancements in the alternative complement pathway, superoxide dismutase, and peroxidase activities. Likewise, the beneficial effect of combined probiotics in the diet have been documented to trigger innate immunity in rainbow trout (*Oncorhynchus mykiss*) [55], *Labeo rohita* [11], olive flounder [33]. Others reported that combined utilization of bacterial strains with complementary properties within the gut environment can enhance or extend the favorable health-promoting effects and immune response of the host [56]. Several authors have reported that external factors such as probiotics, vitamins, and nutrients influence the activity of the alternative complement pathway in fish [57, 58]. In the present study, the activity of the alternative complement pathway emerged as one of the immune system parameters most affected, showing a significant increase in all of the experimental groups. These findings are similar to those described by Giri, Sukumaran [32] and Sun, Yang [38].

It was reported that bactericidal activity is a critical component of the host's defence mechanisms against pathogens [9, 59]. The outcomes obtained in our study indicate that the greatest levels of serum and mucus bactericidal activity were observed in fish receiving diets supplemented with *L. mesenteroides* or/and *L. lactis*. The lysozyme activities in fish have been shown to be influenced by various probiotic species. For instance, in brown trout (*Salmo trutta*), supplementation with *L. mesenteroides* has been reported to affect lysozyme activity [53].

Similarly, in grouper (*Epinephelus coioides*), supplementation with *L. lactis* has been found to modulate lysozyme activity [38]. Consistent with previous findings, in our study, the probiotics *L. mesenteroides* and *L.* lactis have altered the serum lysozyme activity of Nile tilapia. The *L. mesenteroides* and *L. lactis* resulted in increased serum peroxidase values, while the peroxidase content remained unaffected by the mixture diet of *L. mesenteroides* and *L. lactis*, which supports earlier observation in sea bream (*Sparus aurata L*) [26]. Superoxide dismutase facilitates the conversion of highly reactive superoxide radicals into less reactive hydrogen peroxide and plays a crucial role in the primary antioxidant defense mechanisms against oxidative stress. In our study, there was a significant enhancement in superoxide dismutase activities in the experimental group. Comparable findings were reported in *L. rohita*, where dietary supplementation with *Bacillus subtilis* in combination with *Pseudomonas aeruginosa* and *Lactobacillus plantarum*, resulted in similar improvements [32]. Fish possess a distinctive physical barrier comprising epidermal mucus and skin [60], which serves as a component of their nonspecific defense mechanisms, protecting the fish body from direct exposure to environmental stressors and pollutants in the surrounding water. The findings of this study demonstrated an increased secretion of mucus in all fish fed diets supplemented with *L. mesenteroides* or/and *L. lactis* compared to those fed the control diet. Likewise, it has been shown that oral administration of Lactobacillus sp. enhance skin mucus secretion in red sea bream (Pagrus major) and gilthead sea bream (Sparus aurata) [26, 61].

Together current findings showed that the diet supplemented with *L. mesenteroides* and *L. lactis* species exerted more pronounced effects on the innate immune system of Nile tilapia. Consequently, formulations containing multiple probiotic strains were observed to elicit distinct responses in the host immune system compared to formulations containing single probiotic strains. The evidence from this study indicates that in higher vertebrates, well-designed formulations of multistrain or multispecies probiotics have been shown to exhibit health-promoting effects that are lacking in diets containing single-species probiotics [62, 63].

The effects of probiotics vary widely and are contingent upon factors such as their sources, dosage, types, and supplementation period. Therefore, when formulating a multi species probiotic mixture, it is essential to thoroughly select candidate strains to optimize their synergistic effects.

The combination of *L. mesenteroides* and *L. lactis* appears to be a suitable mixture in this context. The synergistic effect enabled by the mixture of *L. mesenteroides* and *L. lactis* potentially contributed to the enhanced performance of Nile tilapia. However, further research is needed to confirm this potential.

Author Contributions: Supervision, conceptualization, writing—original draft preparation, writing—review and editing S.B., K.K.; methodology, validation, formal analysis, A.P., A.N., G.N., N.A., A.A., F.Z., G.Z., Zh.K., S.I., M.K., Z.S., Zh.D., Y.K., R.S., B.A., Y.M., A.S., A.A.

All authors have read and agreed to the published version of the manuscript.

Data Availability Statement: The original data presented in the study are openly available in FigShare at 10.6084/m9.figshare.27050416 .

References

1. Tan, H.Y., S.W. Chen, and S.Y. Hu, *Improvements in the growth performance, immunity, disease resistance, and gut microbiota by the probiotic Rummeliibacillus stabekisii in Nile tilapia (Oreochromis niloticus).* Fish Shellfish Immunol, 2019. **92**: p. 265-275.
2. Wang, M. and M. Lu, *Tilapia polyculture: a global review.* Aquaculture Research, 2016. **47**(8): p. 2363-2374.
3. Won, S., et al., *Effects of Bacillus subtilis WB60 and Lactococcus lactis on growth, immune responses, histology and gene expression in Nile tilapia, Oreochromis niloticus.* Microorganisms, 2020. **8**(1).
4. Defoirdt, T., et al., *Alternatives to antibiotics to control bacterial infections: luminescent vibriosis in aquaculture as an example.* Trends in biotechnology, 2007. **25**(10): p. 472-479.
5. Harikrishnan, R. and C. Balasundaram, *Modern Trends in Aeromonas hydrophila Disease Management with Fish.* Reviews in Fisheries Science, 2005. **13**(4): p. 281-320.
6. Dawood, M.A., S. Koshio, and M.Á. Esteban, *Beneficial roles of feed additives as immunostimulants in aquaculture: a review.* Reviews in Aquaculture, 2018. **10**(4): p. 950-974.
7. Nayak, S.K. and S.C. Mukherjee, *Screening of gastrointestinal bacteria of Indian major carps for a candidate probiotic species for aquaculture practices.* Aquaculture Research, 2011. **42**(7): p. 1034-1041.
8. Liu, H., et al., *Isolation of a putative probiotic strain S12 and its effect on growth performance, non-specific immunity and disease-resistance of white shrimp, Litopenaeus vannamei.* Fish & shellfish immunology, 2014. **41**(2): p. 300-307.
9. Dawood, M.A., et al., *Interaction effects of dietary supplementation of heat-killed Lactobacillus plantarum and β-glucan on growth performance, digestibility and immune response of juvenile red sea bream, Pagrus major.* Fish & shellfish immunology, 2015. **45**(1): p. 33-42.
10. Harikrishnan, R., C. Balasundaram, and M.-S. Heo, *Impact of plant products on innate and adaptive immune system of cultured finfish and shellfish.* Aquaculture, 2011. **317**(1): p. 1-15.
11. Devi, G., et al., *Effect of symbiotic supplemented diet on innate-adaptive immune response, cytokine gene regulation and antioxidant property in Labeo rohita against Aeromonas hydrophila.* Fish & Shellfish Immunology, 2019. **89**: p. 687-700.
12. Devi, G., et al., *Comparative immunostimulatory effect of probiotics and prebiotics in Channa punctatus against Aphanomyces invadans.* Fish & Shellfish Immunology, 2019. **86**: p. 965-973.
13. Merrifield, D.L., et al., *Indigenous lactic acid bacteria in fish and crustaceans.* Aquaculture nutrition: gut health, probiotics and prebiotics, 2014: p. 128-168.
14. Šušković, J., et al., *Antimicrobial activity–the most important property of probiotic and starter lactic acid bacteria.* Food Technology and Biotechnology, 2010. **48**(3): p. 296-307.
15. Seal, B.S., et al., *Alternatives to antibiotics: a symposium on the challenges and solutions for animal production.* Animal Health Research Reviews, 2013. **14**(1): p. 78-87.
16. Dalié, D., A. Deschamps, and F. Richard-Forget, *Lactic acid bacteria–Potential for control of mould growth and mycotoxins: A review.* Food control, 2010. **21**(4): p. 370-380.
17. Ringø, E., et al., *Probiotics, lactic acid bacteria and bacilli: interesting supplementation for aquaculture.* Journal of Applied Microbiology, 2020. **129**(1): p. 116-136.
18. Ray, A.K., K. Ghosh, and E. Ringø, *Enzyme-producing bacteria isolated from fish gut: a review.* Aquaculture nutrition, 2012. **18**(5): p. 465-492.
19. Mohapatra, S., et al., *Use of different microbial probiotics in the diet of rohu, Labeo rohita fingerlings: effects on growth, nutrient digestibility and retention, digestive enzyme activities and intestinal microflora.* Aquaculture Nutrition, 2012. **18**(1): p. 1-11.
20. Ausems, E.J., *THE EUROPEAN CONVENTION FOR THE PROTECTION OF VERTEBRATE ANIMALS USED FOR EXPERIMENTAL AND OTHER SCIENTIFIC PURPOSES.* Zeitschrift Fur Versuchstierkunde, 1986. **28**(5): p. 219-219.
21. Ali, Y., et al., *Classification of lytic bacteriophages attacking dairy Leuconostoc starter strains.* Applied and environmental microbiology, 2013. **79**(12): p. 3628-3636.
22. Allameh, S.K., et al., *Effects of dietary mono- and multiprobiotic strains on growth performance, gut bacteria and body composition of Javanese carp (Puntius gonionotus, Bleeker 1850).* Aquaculture Nutrition, 2016. **22**(2): p. 367-373.

23. Ramos, M.A., et al., *Dietary probiotic supplementation improves growth and the intestinal morphology of Nile tilapia.* Animal, 2017. **11**(8): p. 1259-1269.
24. Yano, T., *Assay of hemolytic complement activity.* Techniques in fish immunology, 1992: p. 131-141.
25. Dawood, M.A., et al., *Effects of dietary supplementation of Lactobacillus rhamnosus or/and Lactococcus lactis on the growth, gut microbiota and immune responses of red sea bream, Pagrus major.* Fish Shellfish Immunol, 2016. **49**: p. 275-85.
26. Salinas, I., et al., *Monospecies and multispecies probiotic formulations produce different systemic and local immunostimulatory effects in the gilthead seabream (Sparus aurata L.).* Fish & shellfish immunology, 2008. **25**(1-2): p. 114-123.
27. Sun, Y.-Z., et al., *Probiotic applications of two dominant gut Bacillus strains with antagonistic activity improved the growth performance and immune responses of grouper Epinephelus coioides.* Fish & shellfish immunology, 2010. **29**(5): p. 803-809.
28. Bayantassova, S., et al., *Knowledge, attitude and practice (KAP) of smallholder farmers on foot-and-mouth disease in Cattle in West Kazakhstan.* Vet Med Sci, 2023. **9**(3): p. 1417-1425.
29. Sun, Y., et al., *Effects of dietary administration of Lactococcus lactis HNL12 on growth, innate immune response, and disease resistance of humpback grouper (Cromileptes altivelis).* Fish & shellfish immunology, 2018. **82**: p. 296-303.
30. Hooshyar, Y., et al., *Effects of Lactobacillus rhamnosus ATCC 7469 on Different Parameters Related to Health Status of Rainbow Trout (Oncorhynchus mykiss) and the Protection Against Yersinia ruckeri.* Probiotics Antimicrob Proteins, 2020. **12**(4): p. 1370-1384.
31. Adeshina, I., M.I. Abubakar, and B.E. Ajala, *Dietary supplementation with Lactobacillus acidophilus enhanced the growth, gut morphometry, antioxidant capacity, and the immune response in juveniles of the common carp, Cyprinus carpio.* Fish Physiol Biochem, 2020. **46**(4): p. 1375-1385.
32. Giri, S.S., et al., *Effects of dietary supplementation of potential probiotic Bacillus subtilis VSG1 singularly or in combination with Lactobacillus plantarum VSG3 or/and Pseudomonas aeruginosa VSG2 on the growth, immunity and disease resistance of Labeo rohita.* Aquaculture Nutrition, 2014. **20**(2): p. 163-171.
33. Beck, B.R., et al., *The effects of combined dietary probiotics Lactococcus lactis BFE920 and Lactobacillus plantarum FGL0001 on innate immunity and disease resistance in olive flounder (Paralichthys olivaceus).* Fish Shellfish Immunol, 2015. **42**(1): p. 177-83.
34. Zhang, H., et al., *Effect of Dietary Supplementation of Lactobacillus Casei YYL3 and L. Plantarum YYL5 on Growth, Immune Response and Intestinal Microbiota in Channel Catfish.* Animals (Basel), 2019. **9**(12).
35. Xia, Y., et al., *Effects of dietary Lactobacillus rhamnosus JCM1136 and Lactococcus lactis subsp. lactis JCM5805 on the growth, intestinal microbiota, morphology, immune response and disease resistance of juvenile Nile tilapia, Oreochromis niloticus.* Fish Shellfish Immunol, 2018. **76**: p. 368-379.
36. Suzer, C., et al., *Lactobacillus spp. bacteria as probiotics in gilthead sea bream (Sparus aurata, L.) larvae: Effects on growth performance and digestive enzyme activities.* Aquaculture, 2008. **280**(1): p. 140-145.
37. Heo, W.-S., et al., *Effects of dietary probiotic, Lactococcus lactis subsp. lactis I2, supplementation on the growth and immune response of olive flounder (Paralichthys olivaceus).* Aquaculture, 2013. **376-379**: p. 20-24.
38. Sun, Y.-Z., et al., *Effect of Lactococcus lactis and Enterococcus faecium on growth performance, digestive enzymes and immune response of grouper Epinephelus coioides.* Aquaculture Nutrition, 2012. **18**(3): p. 281-289.
39. Xia, Y., et al., *Effects of Lactococcus lactis subsp. lactis JCM5805 on colonization dynamics of gut microbiota and regulation of immunity in early ontogenetic stages of tilapia.* Fish & Shellfish Immunology, 2019. **86**: p. 53-63.
40. Adeoye, A.A., et al., *Combined effects of exogenous enzymes and probiotic on Nile tilapia (Oreochromis niloticus) growth, intestinal morphology and microbiome.* Aquaculture, 2016. **463**: p. 61-70.

41. Lara-Flores, M. and M.A. Olvera-Novoa, *The use of lactic acid bacteria isolated from intestinal tract of Nile tilapia (Oreochromis niloticus), as growth promoters in fish fed low protein diets.* Latin American Journal of Aquatic Research, 2017. **41**(3): p. 490-497.
42. Lara-Flores, M., et al., *Use of the bacteria Streptococcus faecium and Lactobacillus acidophilus, and the yeast Saccharomyces cerevisiae as growth promoters in Nile tilapia (Oreochromis niloticus).* Aquaculture, 2003. **216**(1): p. 193-201.
43. Hakim, Y., et al., *Relationship between intestinal brush border enzymatic activity and growth rate in tilapias fed diets containing 30% or 48% protein.* Aquaculture, 2006. **257**(1-4): p. 420-428.
44. Furne, M., et al., *Digestive enzyme activities in Adriatic sturgeon Acipenser naccarii and rainbow trout Oncorhynchus mykiss. A comparative study.* Aquaculture, 2005. **250**(1-2): p. 391-398.
45. Shan, X., et al., *Effects of photoperiod on growth, mortality and digestive enzymes in miiuy croaker larvae and juveniles.* Aquaculture, 2008. **281**(1-4): p. 70-76.
46. Ling, W.H. and O. Hänninen, *Shifting from a conventional diet to an uncooked vegan diet reversibly alters fecal hydrolytic activities in humans.* The Journal of nutrition, 1992. **122**(4): p. 924-930.
47. Bagheri, T., et al., *Growth, survival and gut microbial load of rainbow trout (Onchorhynchus mykiss) fry given diet supplemented with probiotic during the two months of first feeding.* Turkish Journal of Fisheries and Aquatic Sciences, 2008(1): p. 43-48.
48. Carnevali, O., F. Maradonna, and G. Gioacchini, *Integrated control of fish metabolism, wellbeing and reproduction: The role of probiotic.* Aquaculture, 2017. **472**: p. 144-155.
49. Iorizzo, M., et al., *Probiotic Potentiality from Versatile Lactiplantibacillus plantarum Strains as Resource to Enhance Freshwater Fish Health.* Microorganisms, 2022. **10**(2).
50. Thomas, C.M. and J. Versalovic, *Probiotics-host communication: modulation of signaling pathways in the intestine.* Gut microbes, 2010. **1**(3): p. 148-163.
51. Wells, J.M., *Immunomodulatory mechanisms of lactobacilli.* Microbial cell factories, 2011. **10**(Suppl 1): p. S17.
52. Liu, W., et al., *Comparison of adhesive gut bacteria composition, immunity, and disease resistance in juvenile hybrid tilapia fed two different Lactobacillus strains.* Fish & shellfish immunology, 2013. **35**(1): p. 54-62.
53. Balcazar, J.L., et al., *Changes in intestinal microbiota and humoral immune response following probiotic administration in brown trout (Salmo trutta).* Br J Nutr, 2007. **97**(3): p. 522-7.
54. Seisenov, B., et al., *In Vitro Fertilization in Kazakh Whiteheaded Cattle: A Comparative Study.* Life (Basel), 2023. **13**(8): p. 1632.
55. Jasim, S.A., et al., *The synergistic effects of the probiotic (Lactobacillus fermentum) and cinnamon, Cinnamomum sp. powder on growth performance, intestinal microbiota, immunity, antioxidant defence and resistance to Yersinia ruckeri infection in the rainbow trout (Oncorhynchus mykiss) under high rearing density.* Aquaculture Research, 2022. **53**(17): p. 5957-5970.
56. Salinas, I., et al., *Dietary administration of Lactobacillus delbrüeckii and Bacillus subtilis, single or combined, on gilthead seabream cellular innate immune responses.* Fish & Shellfish Immunology, 2005. **19**(1): p. 67-77.
57. Cerezuela, R., et al., *Effects of dietary Bacillus subtilis, Tetraselmis chuii, and Phaeodactylum tricornutum, singularly or in combination, on the immune response and disease resistance of sea bream (Sparus aurata L.).* Fish & Shellfish Immunology, 2012. **33**(2): p. 342-349.
58. Mohammadian, T., et al., *Synbiotic effects of β-glucan, mannan oligosaccharide and Lactobacillus casei on growth performance, intestine enzymes activities, immune-hematological parameters and immune-related gene expression in common carp, Cyprinus carpio: An experimental infection with Aeromonas hydrophila.* Aquaculture, 2019. **511**: p. 634197.
59. Issimov, A., et al., *Prevalence of Clostridium perfringens and Detection of Its Toxins in Meat Products in Selected Areas of West Kazakhstan.* Agriculture-Basel, 2022. **12**(9).
60. Hernandez, L.H.H., et al., *Effects of the commercial probiotic Lactobacillus casei on the growth, protein content of skin mucus and stress resistance of juveniles of the Porthole livebearer Poecilopsis gracilis (Poecilidae).* Aquaculture Nutrition, 2010. **16**(4): p. 407-411.

61. Dawood, M.A.O., et al., *Effects of dietary inactivated Pediococcus pentosaceus on growth performance, feed utilization and blood characteristics of red sea bream, Pagrus major juvenile.* Aquaculture Nutrition, 2016. **22**(4): p. 923-932.
62. Timmerman, H.M., et al., *Monostrain, multistrain and multispecies probiotics—A comparison of functionality and efficacy.* International Journal of Food Microbiology, 2004. **96**(3): p. 219-233.
63. Wang, Y., et al., *Probiotic Potential of Leuconostoc pseudomesenteroides and Lactobacillus Strains Isolated From Yaks.* Frontiers in Microbiology, 2018. **9**.

Click here to access/download;Figure;Figure 1.tif

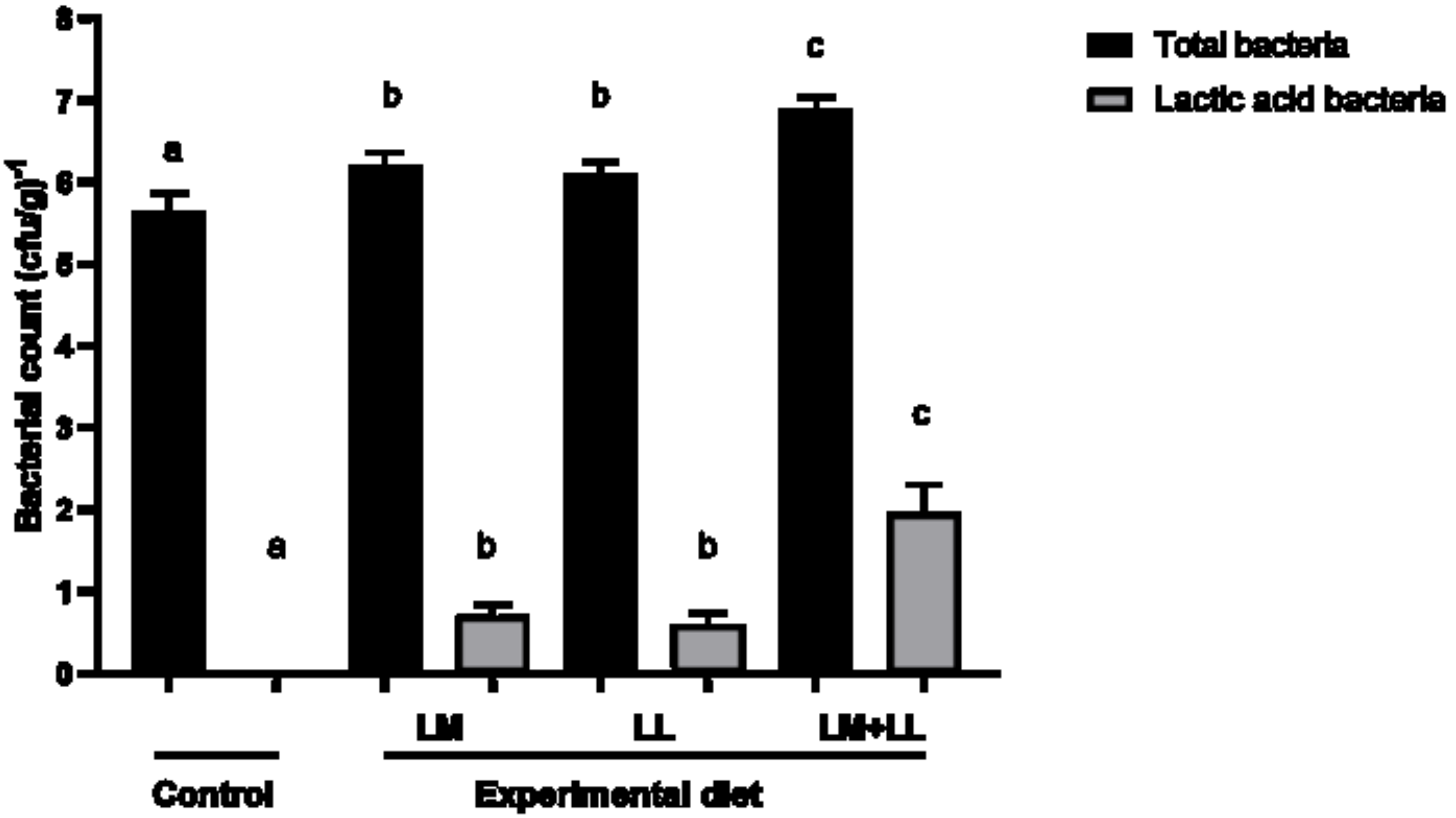

Figure

Click here to access/download;Figure;Figure 2.tif

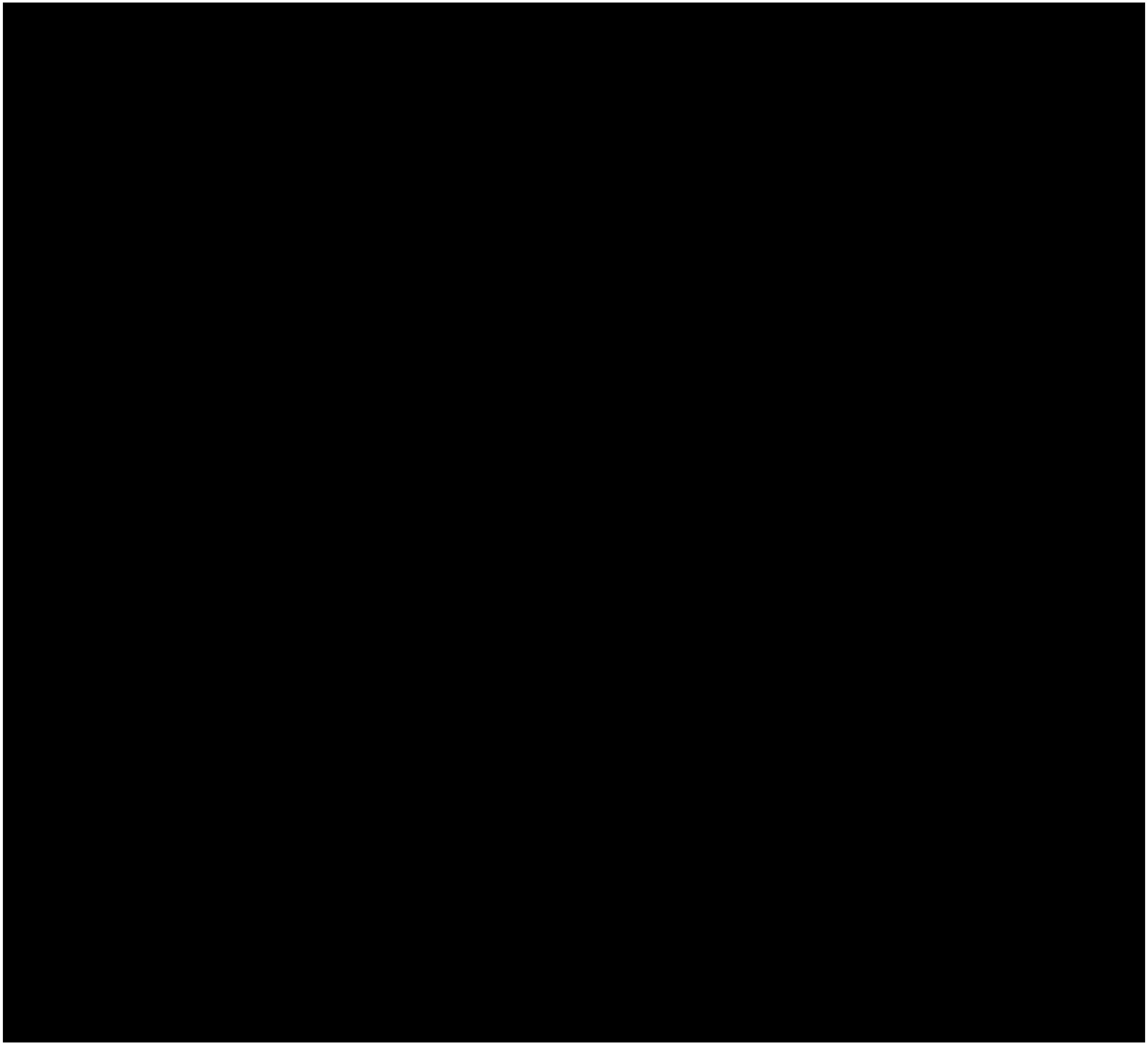

Figure Click here to access/download;Figure;Figure 3.tif

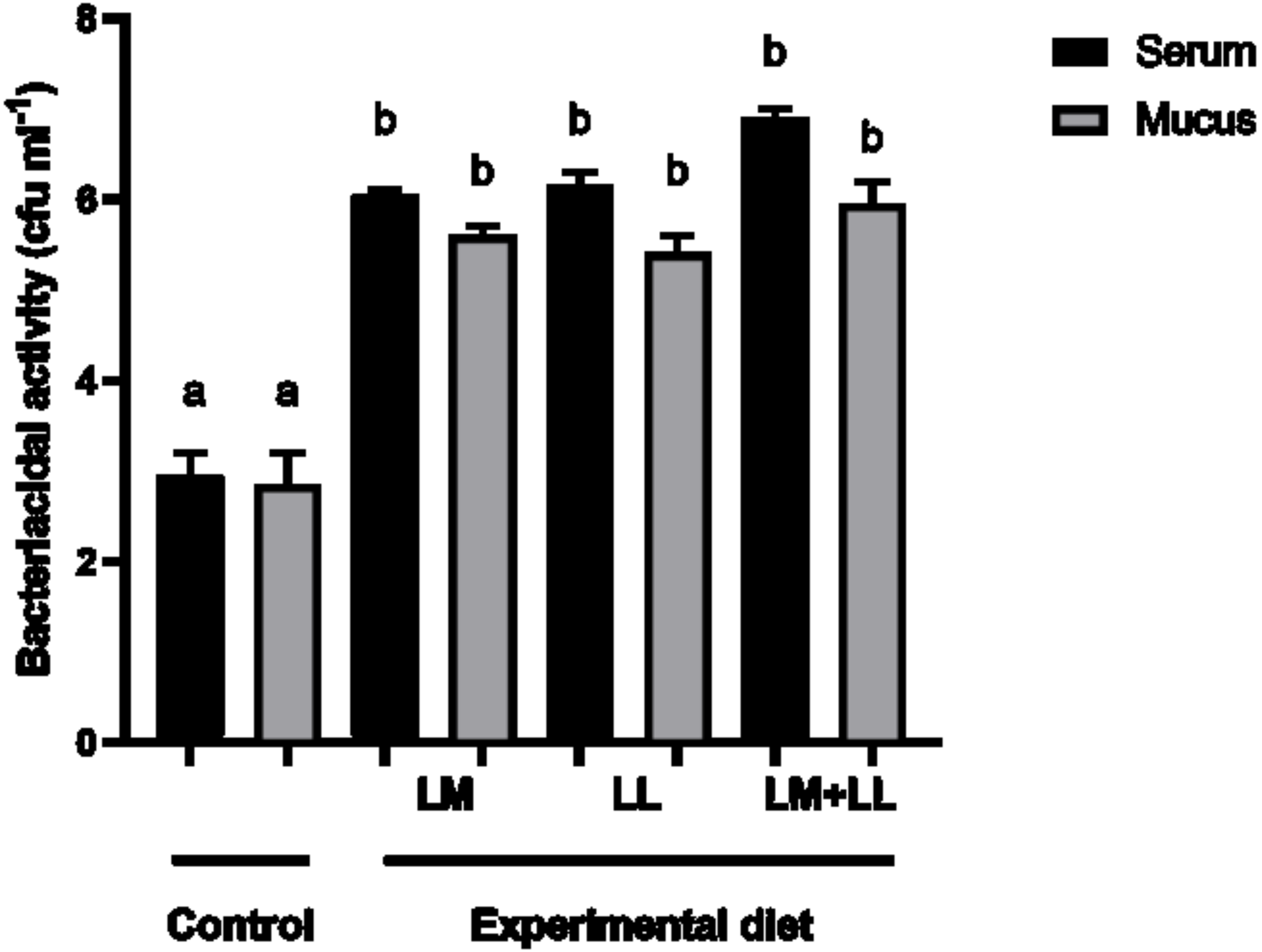

Click here to access/download;Figure;Figure 4.tif

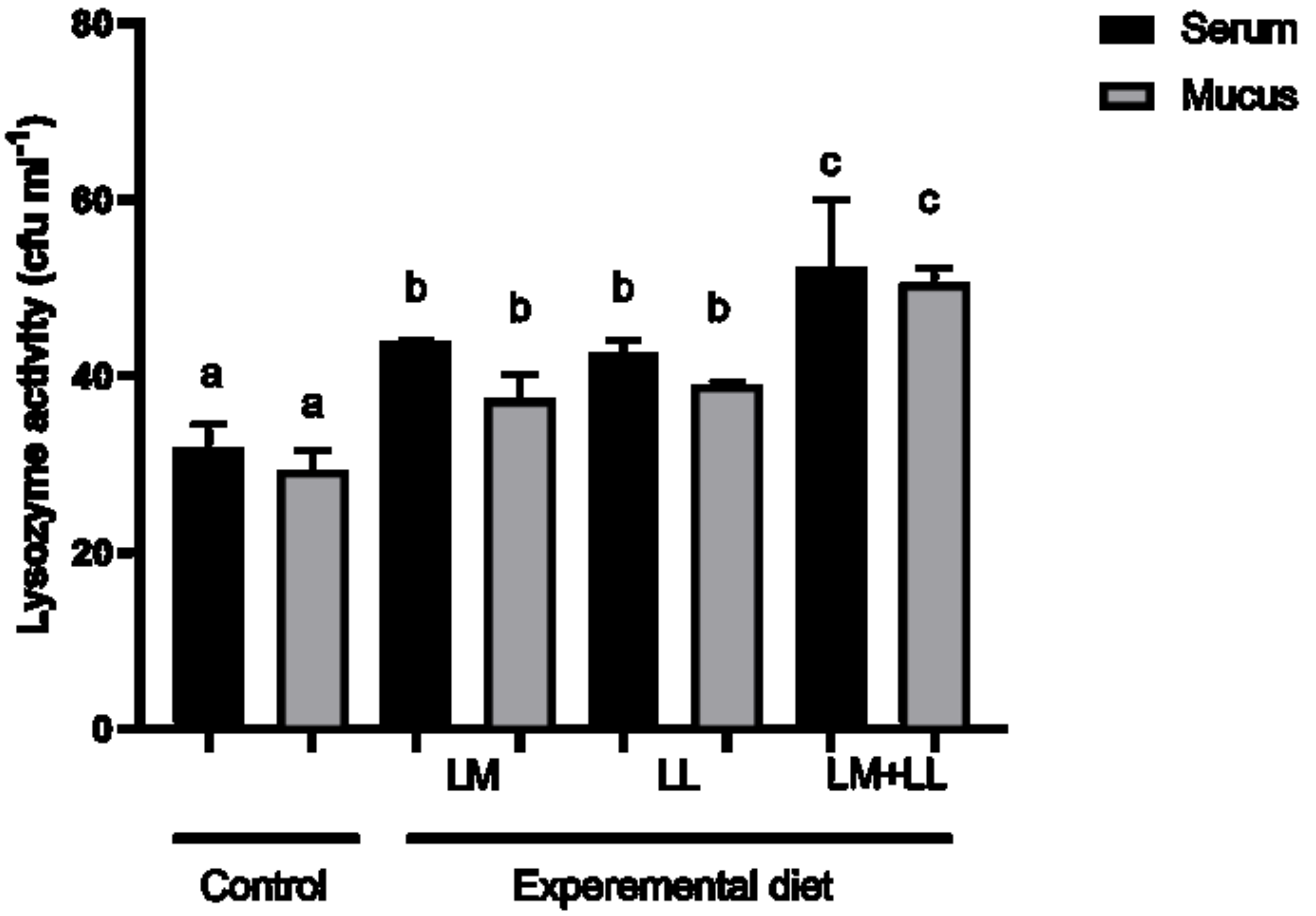

Click here to access/download;Figure;Figure 5.tif

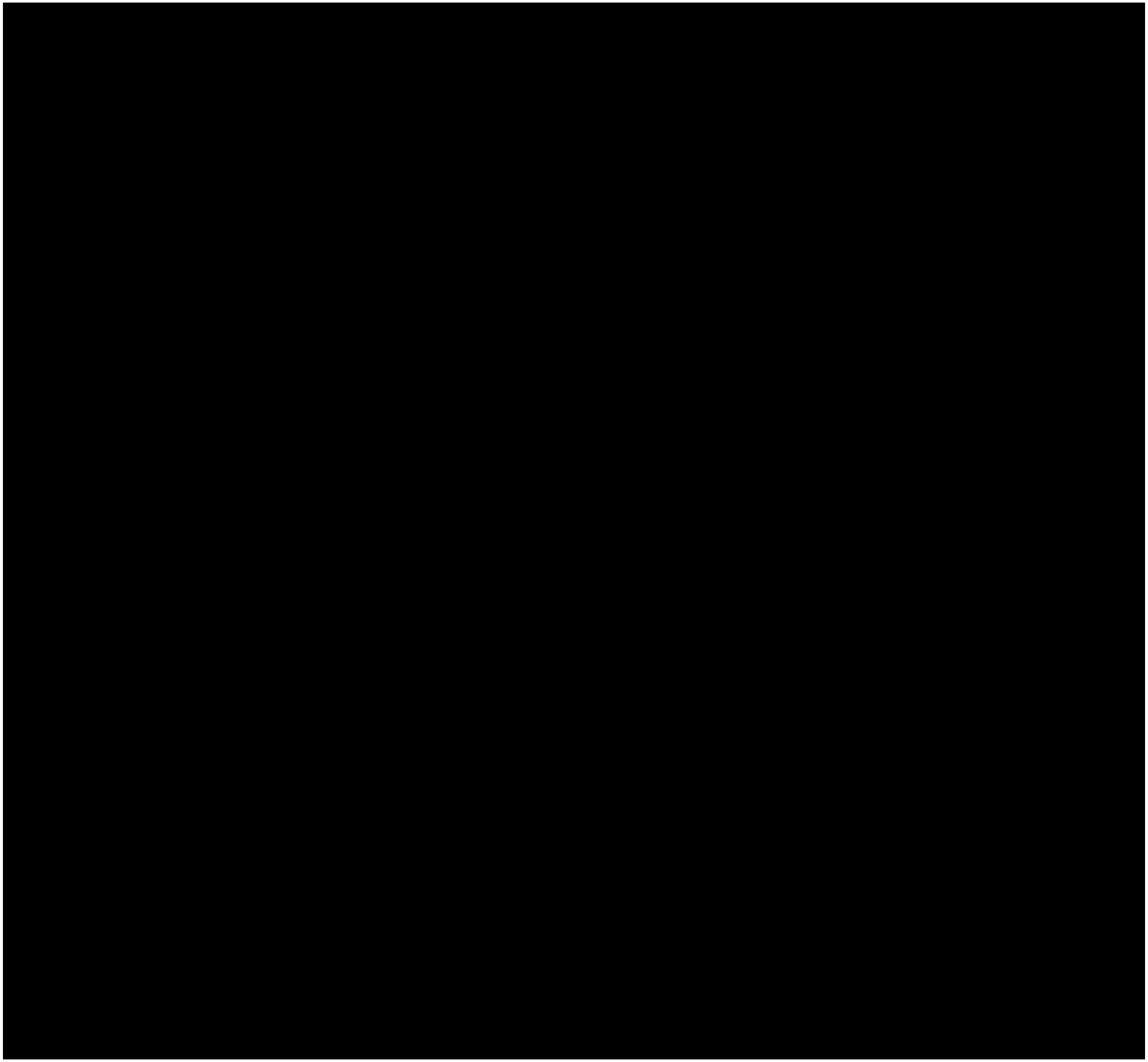

Figure

Click here to access/download;Figure;Figure 6.tif

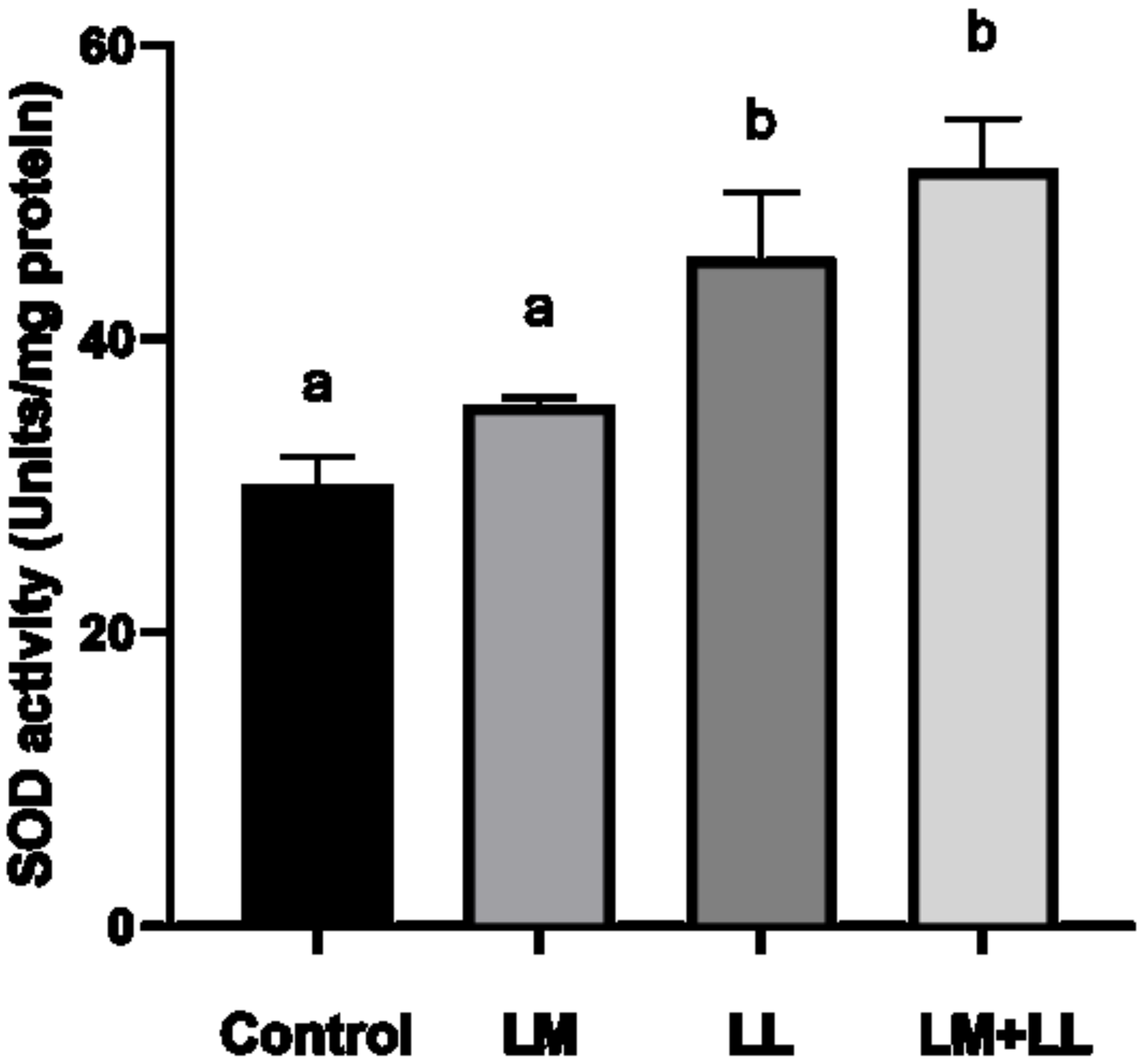

 Click here to access/download;Figure;Figure 7.tif

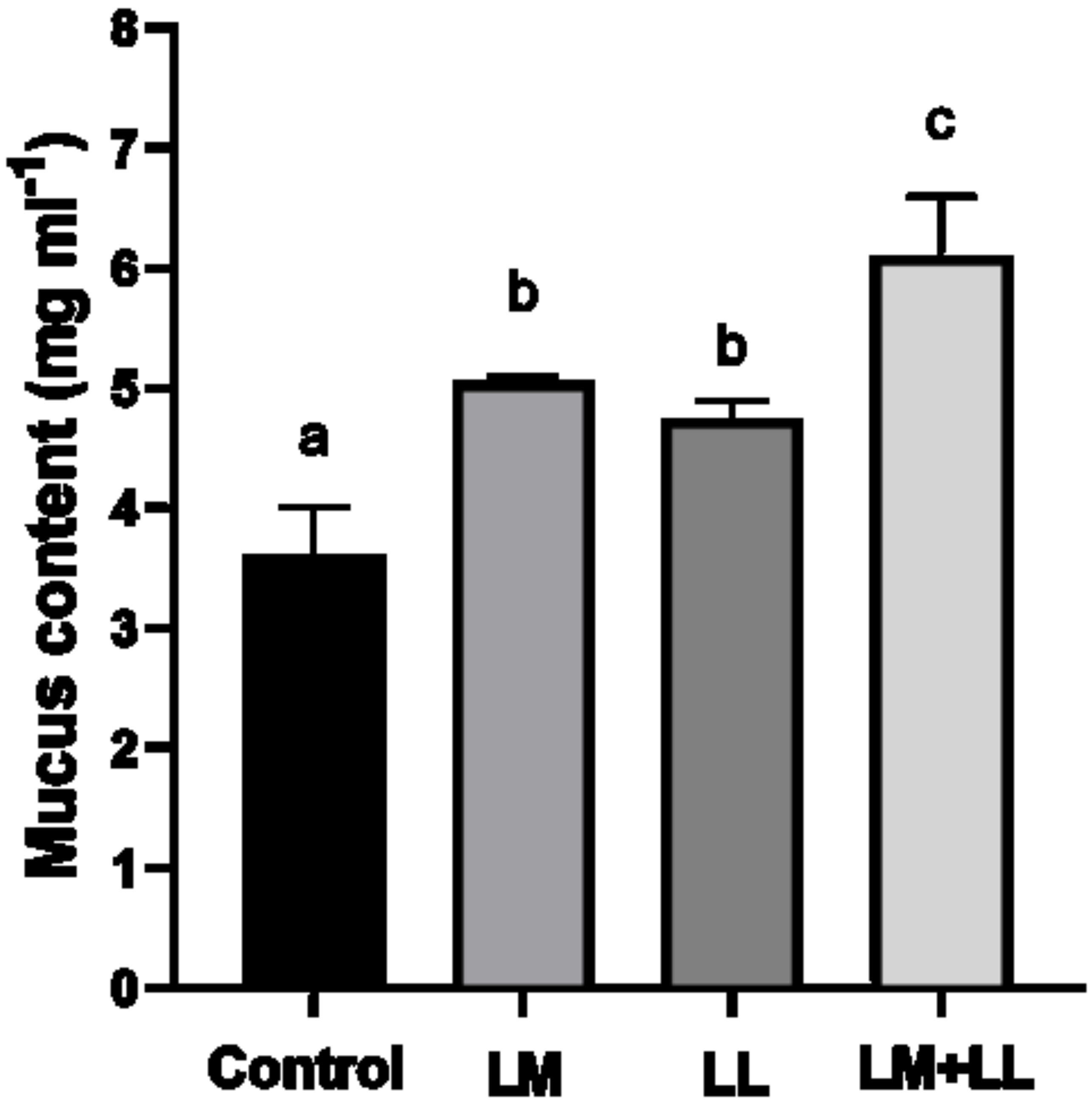

The dietary effects of two strain ~~multistrain~~ probiotics (*Leuconostoc mesenteroides, Lactococcus lactis*) on growth performance, immune response and gut microbiota in Nile tilapia (*Oreochromis niloticus*)

Assel Paritova[1], Akylbek Nurgaliyev[2], Gulbaram Nurgaliyeva[2], Nurzhan Abekeshev[2], Altynay Abuova[3], Faruza Zakirova[2], Grzegorz Zwierzchowski[4], Zhaxygali Kuanchaleyev[1], Saltanat Issabekova[1], Maigul Kizatova[5], Zaure Sayakova[6], Dinara Zhanabayeva[1], Yelena Kukhar[1], Ruslan Stozhkov[1], Botagoz Aitkozhina[1], Yevgeniy Mayer[1], Svetlana Bayantassova[2*], Angsar Satbek[1], Alexandr Andruchshak[1], Kaissar Kushaliyev[2*].

1. Saken Seifullin Kazakh Agrotechnical University, Astana, 010000, Kazakhstan.
2. Zhangir Khan West Kazakhstan Agrarian–Technical University, Uralsk 090000, Kazakhstan.
3. International Engineering and Technological University, Almaty, 050000, Kazakhstan.
4. University of Warmia and Mazury, Olsztyn, 10-719, Poland.
5. S.D. Asfendiyarov Kazakh National Medical University, 050000, Almaty, Kazakhstan.
6. Kazakh Scientific Veterinary Research Institute, 500016, Almaty, Kazakhstan.

*** Corresponding authors:** 1. Svetlana Bayantassova, bayantasova65@mail.ru

2. Kaissar Kushaliyev, kaissark@mail.ru

Field Code Changed

Abstract

The aquaculture industry has been growing rapidly over the past ~~two~~ few decades to meet future animal protein demands. However, intensive aquaculture industry faces challenges such as growth abnormalities, high mortality rates, water quality and intestinal health deterioration. Administering probiotics can serve as a nutritional strategy to enhance the immune system and growth performance of fish influxes of gut microbiota. This study aimed to evaluate the impact of ~~multiple-strain~~two dietary probiotic strains~~s~~ *L. ~~euconostoc~~ mesenteroides* and *L.~~actococcus~~ lactis* on the growth performance, immunity, and gut microbiota of Nile tilapia (*Oreochromis*

niloticus). Fish were fed with basal and experimental diet supplemented by both *L.~~euconostoc~~ mesenteroides* and *L.~~actococcus~~ lactis* bacteria at 10^6 cell/g for 8 weeks. Feeding a combination of *L.~~euconostoc~~ mesenteroides* and *L.~~actococcus~~ lactis* resulted in significant improvements in feed utilization parameters (PER and FER) (*P* < 0.001), alternative complement pathway activity , intestinal lactic acid bacteria count (P < 0.012), mucus secretion (P < 0.002) and peroxidase activity (P < 0.001) compared to the control groups ~~(P < 0.05)~~. Serum lysozyme activity also exhibited a significant increase in the *L.~~euconostoc~~ mesenteroides* and *L.~~actococcus~~ lactis* dietary group (*P* < 0.011) compared to the control and single probiotic supplemented diet groups~~the control group~~. Furthermore, Nile tilapia fed the *L.~~euconostoc~~ mesenteroides* and *L.~~actococcus~~ lactis*- supplemented diet ~~diet~~ showed enhanced growth performance metrics (weight gain, final weight and specific growth rate) compared to those fed ~~the~~ control and single probiotic supplemented diets ~~diet~~ (P < 0.~~05~~022). Additionally, superoxide dismutase activity was significantly elevated in the *L.~~euconostoc~~ mesenteroides* and *L.~~actococcus~~ lactis* supplemented diet groups ~~groups~~ compared to the control and single *L.mesenteroides* supplemented diet groups (P < 0.0175~~5~~). These findings strongly indicate that a dietary combination of *L.~~euconostoc~~ mesenteroides* and *L.~~actococcus~~ lactis* probiotics could function as a beneficial immunostimulant feed supplement in Nile tilapia aquaculture.

Keywords: Nile tilapia, ~~*Leuconostoc mesenteroides, Lactococcus lactis,*~~ probiotic, immunity, growth.

Introduction

Over the past ~~two~~ few decades, there has been considerable growth in the aquaculture industry, positioning it to effectively address the future animal protein demands of the human population [1]. Tilapia ranks among the most extensively farmed fish globally, representing one of the most widely practiced forms of aquaculture.~~[2]~~ They are highly suitable for aquaculture due to their ease of cultivation, rapid growth rates, and relatively high tolerance to environmental stressors

Formatted: Font: Times New Roman, 12 pt, English (United States)

Formatted: Font: Times New Roman, 12 pt, English (United States)

compared to other fish species [2]. Nonetheless, intensive aquaculture encounters challenges that have raised concerns regarding fish health, encompassing growth abnormalities, mortality rates, and disease outbreaks [3]. Consequently, farmers are forced to use prophylactic antibiotics to mitigate these challenges [4, 5].

Antibiotics are commonly employed in aquaculture to manage diseases and enhance growth performance. However, their usage in this context has the potential to disrupt intestinal microbiota and foster the emergence of antibiotic-resistant bacteria, posing risks to aquatic life [5, 6]. Consequently, previous studies aimed to devise alternative feed additives to substitute antibiotics in aquaculture practices [7-10]. The application of probiotics as dietary additives has been recently proposed as a viable alternative to antibiotics [11, 12]. Their application aims to reduce the colonization gastrointestinal mucosa by pathogenic population~~bacteria~~, thereby enhancing the health of mammals and fish [13-15]. Additionally, lactic acid bacteria (LAB) were ability to increase~~have the capability to enhance~~ growth performance and survival rates, prevent intestinal disorders such as diarrhea, neutralize antinutritional factors [16, 17]. Moreover, advantageous intestinal microbiota can produce enzymes that have the potential to enhance the digestibility and uptake of nutritional ~~dietary~~ components [18]. It was documented that incorporating a combination of probiotics into the diet of *Labeo rohita* fingerlings resulted in improved nutrient digestibility, enhanced growth, a higher protein efficiency ratio, and a reduced feed conversion ratio compared to the use of individual strains [19]. In this study, we have used two probiotics, L. mesenteroides and L. lactis, included in the diet of Nile tilapia (*Oreochromis niloticus*). These LAB were isolated from the gastrointestinal tracts of different fish species (e.g. northern pike, zebrafish, carp,). They are important in aquaculture as they help balance the intestinal microbiota, enhance growth performance, improve digestion and nutrient absorption, and boost the immune response ~~In this study, we have used a combination of two probiotics Leuconostoc mesenteroides and Lactococcus lactis included in the diet of Nile tilapia. These probiotics were isolated from gastrointestinal tract of different fish species[13, 20].~~

Formatted: Font: Times New Roman, 12 pt, English (United States)

Formatted: Font: Times New Roman, 12 pt, English (United States)

Formatted: Default Paragraph Font, Font: Times New Roman, 12 pt, Russian

Formatted: Font: Times New Roman, 12 pt, Not Italic

Formatted: Font: Times New Roman, 12 pt, Not Italic, English (United States)

Formatted: Font: Times New Roman, 12 pt, Not Italic

Formatted: Font: Times New Roman, 12 pt, Not Italic, English (United States)

Formatted: Font: Times New Roman, 12 pt, Not Italic

Formatted: Font: Times New Roman, 12 pt, Not Italic, English (United States)

Formatted: Font: Times New Roman, 12 pt, Not Italic, English (United States)

Formatted: Font: Times New Roman, 12 pt, Not Italic

Formatted: Font: (Default) Times New Roman, 12 pt, Not Italic, Font color: Auto, English (United States), Pattern: Clear

Formatted: Font: Times New Roman, 12 pt, Not Italic

Formatted: Font: Times New Roman, 12 pt, Not Italic, English (United States)

Formatted: Font: Times New Roman, 12 pt, Not Italic

Formatted: Font: Times New Roman, 12 pt, Not Italic, English (United States)

Formatted: Font: Not Italic

Formatted: Font: Not Italic

Formatted: Font: Not Bold, Not Italic

This study examined the effect of two probiotic strains ~~multiple-strain dietary probiotics~~ in fresh water fish Nile tilapia ~~freshwater aquaculture~~ on growth performance, cultivable gut bacteria, and innate immune response, as well as body weight gain ~~in Nile tilapia~~.

Materials and Methods

Animal Ethics

The experiment was carried out according to the European Convention for the Protection of Vertebrate Animals used for Experimental and Other Scientific Purposes guidelines [20]. The protocol was approved by the Committee on the Ethics of Animal Experiments of the Saken Seifullin Kazakh Agrotechnical University (permit number: 2).

Experimental design

Overall, ~~192~~ 395 healthy juvenile Nile tilapia with body weight averaging 45±2g were purchased from local commercial fish farm. Fish were then equally divided in ~~two~~ four groups in 500L tank: control group without probiotic supplementation (C) 96 heads and three experimental groups *L. mesenteroides (LM), L. lactis (LL) and a combination of both (mix)* – each consisting of 96 ~~heads respectively~~fish per group. Subsequently, the fish were randomly distributed into ~~six~~ twelve 100-liter tanks, with thirty-two fish per tank and two replicate tanks per treatment. Each tank was fitted with both an inlet and an outlet and maintained in a flow-through freshwater system. Prior experiment onset, fish allowed to acclimatize in 500L tank with 5mg/L O_2 aerated water for 14 days. Water quality parameters were assessed by employing a thermometer to measure temperature and an oxygen meter (Model FEBO 330, EPF Instruments, Fisher Scientific, USA) to measure dissolved oxygen levels. Throughout the experiment, water temperature was maintained at 27℃. The pH level was monitored with a portable pH meter (Fisherbrand AP 115). During the acclimatization period fish were fed a control diet without probiotic supplements *ad libitum* twice a day at 9:00 a.m. and 17:00 p.m. After completing the feeding trial (8 weeks), all experimental fish subjected a 24-h~~our~~ fasting. Following this, survival

Formatted: Font: Times New Roman, 12 pt, Not Italic, English (United States)

Formatted: Font: Not Italic

rate, individual body weight~~ and length~~, immune parameters, and gut microbiota composition of fish from each tank were determined.

Isolation of Probiotics

L. ~~*euconostoc*~~ *mesenteroides* and *L.*~~*actococcus*~~ *lactis* LAB starins were isolated from intestines of common carp (*Cyprinus carpio*) fingerlings following protocol described by [3]. In brief, a 50 µl sample of intestinal microflora from fish was dissolved in 500 µl distilled water (1:10 ratio). Subsequently, using a disposable pipette, 100 µl of the suspension was introduced to the MRS agar medium and incubated at 37°C for 48~~-~~h. Glass beads were utilized to ensure uniform growth of isolated colonies. Eight to twelve glass beads (pre-sterilized) were placed into the Petri dish containing the inoculum. The probiotics obtained were washed three times in distilled water and plates were sent for genomic confirmation.

PCR

For DNA extraction, GeneJET PCR Purification Kit (Thermo Fisher Scientific, USA) was used according to the manufacturer's instructions. Following reference strains were utilized as a positive controls: National Collection of Type Culture (NCTC) AB023246 for *L.*~~*euconostoc*~~ *mesenteroides* and (NCTC) M58837 for *L.*~~*actococcus*~~ *lactis*. For genotyping of isolates, multiplex PCR (mPCR) was run according to the protocol described by Ali, Kot [21]. The amplification of 16S mDNA fragments in a Thermal Cycler (Eppendorf Mastercycler) were as follows: 95~~-~~◦C for 2 min 30 s, 95~~-~~◦C for 1 min, 55~~-~~◦C for 1 min, 72~~-~~◦C for 1 min 20 s (35 cycles), and 72~~-~~◦C for 2 min. The PCR products obtained were administered in 1% agarose gel electrophoresis in the presence of Ethidium bromide, and the results were visualized using Bio-Imaging Systems (MiniBIS Pro, Israel).

Formatted: Font: Not Italic

Preparation of Experimental Diets

Commercial feed (F.lli Fragola, S.P.A, Italy) without probiotic supplements was used as a basal control diets. Basal diet composed of fishmeal, soybean meal, milled wheat, extruded peas, potato starch, vegetable oil, tricalcium phosphate, premix, yeast (Table 1).

Formatted: Font: Times New Roman, 12 pt, Not Bold, Not Italic, Font color: Auto, English (United States), Not All caps, Not Expanded by / Condensed by , Pattern: Clear

Formatted: Font: Times New Roman, 12 pt, Not Bold, Not Italic, Font color: Auto, English (United States), Not All caps, Not Expanded by / Condensed by , Pattern: Clear

The culture medium was prepared using a water-based solution with the addition of up to 1.5% glucose. Activated strains of *L. lactis* and *L. mesenteroides* were used as inoculants, added at a concentration of 0.05%. Briefly, in distilled water, the following components were added: 1.5% glucose, 0.1% buffer salts, 0.1% ascorbic acid, 5% peptone, 1.5% microbiological agar, and 0.5% yeast. The pH of the medium was adjusted to (7.0±0.1). The prepared medium was sterilized at 121°C for 30 minutes, then cooled to (37±1)°C. Then, an inoculum containing the activated strains *L. lactis* and *L. mesenteroides* at a concentration of 0.05% was introduced. Biomass cultivation was carried out under batch fermentation for (24±2) hours, with pH neutralization using Na_2CO_3 at the 12-hour mark. After cultivation, the upper layer of the culture broth was separated, and the bacterial suspension was cooled to (4±2)°C, dispensed into aseptic vials (10-12 ml), sealed, and labeled. The experimental diet was prepared by introducing 1×10^7 CFU/g of ~~each~~ a single *L.~~euconostoc~~ mesenteroides* or/and *L.~~actococcus~~ lactis* and mixed probiotic ~~-~~in the basal diet. To do so, these LAB were incorporated into the basal diet using the spraying method while stirring. Then, 165g of gelatin was dissolved in 500 ml of distilled water to create a waterproof membrane for the feed. Next, the dietary components underwent grinding into a fine powder, followed by thorough agitation and subsequent cold extrusion into pellets 0.5 mm in diameter. Finally, the diluted gelatin was sprayed onto LAB incorporated basal diet pellets under thorough stirring. The complete diet were then air-dried overnight at room temperature and stored in plastic containers at −4°C until required.

Table 1. Composition and nutrient levels of the basal diet (g kg-1) ~~Formulation of the basal diet for Nile tilapia, *Oreochromis niloticus*.~~

**~~Feed i~~Ingredients**	**~~g kg^{-1}~~Diet**
Fishmeal	30
Soybean meal	42
Milled wheat	7
Extruded peas	8
Potato starch	6.7

Formatted: Font: Times New Roman, 12 pt, Not Italic
Formatted: Font: Times New Roman, 12 pt
Formatted: Font: Times New Roman, 12 pt, Not Italic
Formatted: Font: Times New Roman, 12 pt
Formatted: Font: Times New Roman, 12 pt, Not Italic
Formatted: Font: Times New Roman, 12 pt, Not Italic
Formatted: Font: Times New Roman, 12 pt, Not Italic
Formatted: Font: Times New Roman, 12 pt, Not Italic
Formatted: Font: Times New Roman, 12 pt
Formatted: Font: Times New Roman, 12 pt, Not Italic
Formatted: Font: Times New Roman, 12 pt
Formatted: Font: Times New Roman, 12 pt, Not Italic
Formatted: Font: Times New Roman, 12 pt, Not Italic
Formatted: Font: Not Italic
Formatted: Font: Times New Roman, 12 pt, Not Italic, English (United States)
Formatted: Font: Times New Roman, 12 pt, Not Italic
Formatted: Font: Times New Roman, 12 pt, Not Italic, Not Superscript/ Subscript
Formatted: Font: Bold, Not Italic
Formatted Table
Formatted: Font: Bold
Formatted: Font: Bold, Not Italic
Formatted: Font: Bold

Vegetable oil	4
Vitamin/Mineral mixture	1.1
Premix	0.1
Yeast	0.05
Gelatin	1
**Proximate composition ~~%~~**	
Moisture	8.79
Crude protein	35.8
Crude lipid	7.39
Crude ash	6.44

Formatted: Font: Bold, Not Italic
Formatted: Font: Bold
Formatted: Font: (Default) Times New Roman, Not Italic
Formatted: Font: (Default) Times New Roman, Not Italic
Formatted: Font: (Default) Times New Roman, Not Italic
Formatted: Font: (Default) Times New Roman, Not Italic

Growth performance

The survival rate was calculated according to following equation: Survival (%) = 100 × (final number of fish/initial number of fish). The calculation of weight gain (WG), feed conversion ratio (FCR), protein efficiency ratio (PER) and specific growth rate (SGR) were performed according to protocol described by Allameh, Yusoff [22]. In brief, WG in grams is calculated by subtracting the initial weight from the final weight. SGR is determined by the formula: SGR = 100 (ln W2 - ln W1) / T, where W1 and W2 represent the initial and final weights, respectively, and T signify the number of days during the feeding period. SGR indicates the percentage of growth per day for each fish.FCR is computed as the ratio of feed intake in grams to weight gain in grams, while PER is determined by the ratio of weight gain in grams to protein intake in grams.

Determination of Intestinal bacteria activities

To determine the total intestinal microbiota and LAB count, eleven fish were sampled from each study group following 24-h feeding cessation. Prior experiment onset, fish were anesthetized using tricaine methanesulphonate (MS 222) and then transferred on to sterile stainless desk. Before opening, fish carcasses were rinsed with 70% ethanol to eliminate surface contamination. The whole intestine was extracted, and 1 cm sections from both the anterior and posterior ends were discarded. Then the whole intestinal tract was dissected, and rinsed three times in PBS.

Subsequently, tenfold serial dilution was run in PBS. 100 ml aliquots of the diluted solution were then plated onto duplicate Trypto-Soya agar (TSA) media to determine total bacterial counts. To isolate viable LAB species, 100 ml of each dilution was introduced in MRS (DeMan, Rogosa, and Sharpe) agar and allowed to incubate at 25°C for 3 to 5 days. Colony-forming units of viable bacterial populations were enumerated according to protocol reported by Ramos, Batista [23].

Immune responses

Alternative complement pathway activity (ACH50) was determined according to protocol of Yano [24]. In brief, 0.1ml aliquots of diluted serum were transferred in to plastic tubes and adjusted to a total volume of 0.25 ml using barbitone buffer. Rabbit red blood cells (RaRBC) were then added to each tube in a volume of 0.1 ml and allowed to incubate for 1-h at 20°C. Next, after incubation, 3.15 ml of 0.9% NaCl solution was introduced and samples were centrifugated at 1600 x g for 10 minutes at 4°C to remove RaRBC derbies. Finally, obtained supernatant was measured for optical density at 414 nm, and a lysis curve was generated. The volume resulting in 50% hemolysis was identified and utilized to assess the complement activity of the sample.

The bactericidal activity of serum and mucus was assessed following the method described by Dawood, Koshio [25]. To do so, samples were diluted in 1:1 ratio with Tris buffer adjusted to pH 7.5. Then, diluted mixture was agitated with pre-prepared 0.001 mg/ml *Escherichia coli*, IAM1239 bacterial suspension (RIBSP, Kazakhstan) and allowed to incubate overnight at room temperature. The standard plate counting method was employed to calculate colony-forming units (CFU).

To measure lysozyme activity in serum and mucus samples a turbidimetric assay was utilized. Briefly, 10 µl of samples were mixed with 190 µl of *Micrococcus lysodeikticus* bacterium (0.2 mg/PBS adjusted to pH 7.4) in 96 flat-bottomed well plates, and the mixture was allowed to incubate for 5 min at room temperature. Absorbance was measured at 450 nm using Biotec 800

TS (Agilent Technologies, USA). Lysosome activity was determined based on the amount of sample resulting in a decrease in optical density of 0.001 min^{-1}.

The total peroxidase content in serum was assessed according to method described by Salinas, Abelli [26]. To do this, 15 µl of serum and 35 µl of Hank's Buffered Salt Solution were added to 96 flat-bottomed well plates. Subsequently, 50 µl of 3,3′,5,5′-Tetramethylbenzidine (TMB, Thermo Scientific, USA) was added to each well. The diluted serum was then incubated for 20 min~~utes~~, and the color-developing reaction was halted by introducing 50 µl of 2 M sulfuric acid. The optical density was measured at a wavelength of 450 nm using a plate reader.

Serum superoxide dismutase (SOD) activity was quantified by the percentage inhibition rate of the enzyme's reaction using Ransod kit (Randox, Crumlin, UK) following the protocol described by Sun, Yang [27]. The specific SOD activity units were adjusted by milligrams of protein (mg $protein)^{-1}$. One unit of SOD is determined as the quantity of the enzyme present in 20 ml of the sample solution, which inhibits the reduction reaction of nitroblue tetrazolium (NBT) by 50% at wavelength of 550 nm.

Statistical analysis

Graphs were compiled and statistical analyses were conducted to protocol described by Bayantassova, Kushaliyev [28] using Prism 9 software (GraphPad Software, Inc., San Diego, CA). The data were subjected to Kolmogorov–Smirnov and Levene's tests to assess the normality of the distribution and verify the homogeneity of variances. D~~The d~~ata were analysed ~~underwent evaluation through a variety of statistical tests, including Student's t-test,~~by one-way ANOVA with Tukey's post hoc ~~analysis~~test. Statistical significance between groups was denoted when P < 0.05 using asterisks, as detailed in the respective figures.

Results

Demonstration of growth performance, nutrient utilization and survival.

The growth parameters, feed uptake and survival of control and fish fed *L.~~euconostoc~~ mesenteroides* or/and *L.~~actococcus~~ lactis* diet are presented in Table 2. Fish fed the mixture of

Formatted: Font: Times New Roman, 12 pt, English (United States)

Formatted: Font: Times New Roman, 12 pt

Formatted: Font: Not Italic

L.~~euconostoc~~ mesenteroides and *L.~~actococcus~~ lactis* diet demonstrated a substantial increase in ~~weight gain (~~WG~~)~~, ~~final body weight (~~FBW~~)~~, and ~~specific growth rate (~~SGR~~)~~ (*P* < ~~≤~~ 0.~~05~~033) compared to the groups fed single *L. mesenteroides* or the control diet ~~the control groups~~. Additionally, the ~~protein efficiency ratio (~~PER~~)~~ and ~~feed efficiency ratio (~~FER~~)~~ profiles were significantly higher in fish fed on mixture of *L.~~euconostoc~~ mesenteroides* and *L.~~actococcus~~ lactis* diet (*P* <~~≤~~ 0.~~05~~001), whereas these profiles did not differ between single probiotic supplemented groups (P > 0.991) ~~.~~ Moreover, FER profile did not differ significantly between fish fed single probiotic supplemented diets and those fed control diet. ~~Moreover~~Furthermore, a significant increase in the protein gain (PG) profile was observed in fish fed *L.~~euconostoc~~ mesenteroides* or/and *L.~~actococcus~~ lactis*-containing diet compared to the control group (*P* <~~≤~~ 0.~~05~~021). Feed intake profile was enhanced by both *L. mesenteroides* and *L. lactis* dietary treatments, with fish receiving only *L. lactis* exhibiting significantly greater feed intake compared to the control group (P < 0.041). However, no significant difference was observed between fish fed single probiotic supplemented diets (P > 0.087). ~~Fish fed on experimental diet showed higher feed intake (FI) profile, however no significant difference was observed between study groups (*P* ≥ 0.05).~~

Survival rates were high across all treatment groups, and no mortality was observed.

Table 2. Growth performance of Nile tilapia ~~(Oreochromis niloticus)~~ fed with ~~*Leuconostoc*~~ *L. mesenteroides* or/and *L.~~actococcus~~ lactis* supplemented diets for 8 weeks.

	Diets			
	Control	*L. mesenteroides* ~~Mixture of *Leuconostoc mesenteroides* and *Lactococcus lactis* probiotics~~	*L. lactis* probiotics	Mixture of *L. mesenteroides* and *L. lactis* probiotics
[1]IBW (g)	46.3±1.32	45.4±3.44~~45.8±2.11~~	45.9±1.02	45.8±2.11
[2]FBW (g)	68.4±3.26[a]	79.2±1.16[b]~~92.4±4.54*~~	78.5±2.15[b]	92.4±4.54[c]
[3]WG (%)	22.4±3.01[a]	31.7±1.52[b]~~47.3±2.~~	31.2±1.74[b]	47.3±2.02[c]

Formatted: Font: (Default) Times New Roman, 12 pt, Not Italic, Font color: Auto, Pattern: Clear

Formatted: Font: Times New Roman, 12 pt

Formatted: Font: Times New Roman, 12 pt, English (United States)

Formatted: Font: Not Italic

Formatted: Font: Times New Roman, 12 pt, English (United States)

Formatted: Font: Times New Roman, 12 pt, English (United States)

Formatted: Font: Times New Roman, 12 pt

Formatted: Font: Times New Roman, 12 pt, English (United States)

Formatted: Font: Times New Roman, 12 pt, English (United States)

Formatted: Font: Times New Roman, 12 pt, English (United States)

Formatted: Font: Times New Roman, 12 pt, English (United States)

Formatted: Font: Times New Roman, 12 pt, English (United States)

Formatted Table

Formatted: Centered

Formatted: Superscript

Formatted: Centered

Formatted: Not Superscript/ Subscript

Formatted: Superscript

Formatted: Centered

		~~02*~~		
[4]SGR (%)	1.96±0.11^{a}	2.32±0.5^{b}~~2.82±0.2*~~	2.30±0.3^{b}	2.82±0.2^{c}
[5]FI (g)	12.42±0.47^{a}	12.88±1.05^{ab}~~13.24±0.8~~	13.01±0.6^{b}	13.24±0.8^{b}
[6]FER	2.33±0.2^{a}	2.64±0.8^{ab}~~2.97±0.5*~~	2.60±1.12^{ab}	2.97±0.5^{c}
[7]PER	3.05±0.4^{a}	3.44±0.18^{b}~~3.65±0.03*~~	3.52±0.7^{b}	3.65±0.03^{c}
[8]PG	141.23±7.2^{a}	158.42±2.13^{b}~~179.31±4.05*~~	162.32±3.04^{b}	179.31±4.05^{c}

[1]IBW - initial body weight; [2]FBW - final body weight; [3]WG - weight gain; [4]SGR - specific growth rate; [5]FI – feed intake; [6]FER - feed efficiency ratio; [7]PER - protein efficiency ratio; [8]PG - protein gain. ~~* *p* - value ≤ 0.05.~~ Values represent means ± SEM of triplicate groups. Different superscripts in the line represent significant difference $p \leq 0.05$.

Microbiological analyses of gut microbiota

Both the control and experimental groups showed no detectable LAB bacteria in the intestines before the experiment. ~~However,~~ ~~f~~Fish fed with single and the mixture of LAB-supplemented diets demonstrated significantly greater levels of ~~*Leuconostoc mesenteroides and Lactococcus lactis* in~~ the total intestinal microbiota compared to the control group. ~~-~~(P < 0.01~~2~~5) (Figure 1). Moreover, fish fed with the mixture of LAB-supplemented diets exhibited significantly higher cultivable LAB compared to the fish groups fed with single diets (P < 0.027) (Figure 1).~~Moreover, no LAB bacteria were detectable in the intestine of fish fed control diet.~~

Formatted: Superscript
Formatted: Centered
Formatted: Superscript
Formatted: Centered
Formatted: Superscript
Formatted: Superscript
Formatted: Superscript
Formatted: Centered
Formatted: Superscript
Formatted: Centered
Formatted: Superscript
Formatted: Centered
Formatted: Kazakh

Figure 1. Comparison of total bacteria and LAB content of Nile tilapia intestines fed with control and probiotics supplemented diets ~~experimental diets~~ for 8 weeks. Data presented as the mean ±SEM from two combined independent experiments (n=10 fish/group). Bars with different alphabet are statistically different ($P \leq 0.05$). Bars with the same alphabet are statistically insignificant different ($P \geq 0.05$). * LM; LL ~~–~~ _ *L*.~~*euconostoc*~~ *mesenteroides*; *L*.~~*actococcus*~~ *lactis*.

Demonstration of Immune responses

The rate of ~~alternative complement pathway (~~ACP~~)~~ were significantly elevated in fish fed *L*.~~*euconostoc*~~ *mesenteroides* or/and *L*.~~*actococcus*~~ *lactis* supplemented diets than that of control group (Figure 2). Likewise, serum and mucus bactericidal activity (Figure 3), mucus lysozyme activity (Figure 4), peroxidase activity (Figure 5)~~, superoxide dismutase (Figure 6)~~ demonstrated a considerable elevation in fish fed with single or mixture of probiotics supplemented diets ~~experimental diets~~ compared to control group. Similarly, superoxide dismutase inhibition rate was exhibited significantly greater in fish groups fed with single *L. lactis* and mixture diets compared to other groups (Figure 6). Moreover, peroxidase activity (Figure 5) and ACP levels (Figure 2) in fish fed the combined L. mesenteroides and L. lactis diet were significantly elevated compared to those in fish fed single probiotic supplemented diets.

Additionally, when compared with the control and single probiotic supplemented diet groups, lysozyme activities of serum was significantly higher in fish fed mixture of *L*.~~*euconostoc*~~ *mesenteroides* and *L*.~~*actococcus*~~ *lactis* diets (*P* < ~~≤~~ 0.~~05~~011; Figure 4). Furthermore, the amounts of secreted mucus was significantly higher in fish groups supplemented with *L*.~~*euconostoc*~~ *mesenteroides* or/and *L*.~~*actococcus*~~ *lactis* diets compared to control group (*P* < ~~≤~~ 0.~~05~~002; Figure 7).

Formatted: Space After: 0 pt

Formatted: Font: Times New Roman, 12 pt, Not Italic

Formatted: Font: Times New Roman, 12 pt, Not Italic

Formatted: Font: Times New Roman, 12 pt, Not Italic, English (United States)

Formatted: Font: Times New Roman, 12 pt, Not Italic, English (United States)

Formatted: Font: Times New Roman, 12 pt, English (United States)

Formatted: Font: Times New Roman, 12 pt, Not Italic, English (United States)

Formatted: Font: Not Italic, Font color: Auto, Pattern: Clear

Figure 2. Alternative complement pathway of Nile tilapia fed with control and probiotics supplemented dietsexperimental diets. Data presented as the mean ±SEM from two combined independent experiments (n=10 fish/group). Bars with different alphabet are statistically different ($P \leq 0.05$). Bars with the same alphabet are statistically insignificant different ($P \geq 0.05$). * LM; LL – *L. euconostoc mesenteroides*; *L. actococcus lactis*.

Figure 3. Serum and mucus bactericidal activity of Nile tilapia fed with control and probiotics supplemented diets~~experimental diets~~. Data presented as the mean ±SEM from two combined independent experiments (n=10 fish/group). Bars with different alphabet are statistically different ($P \leq 0.05$). Bars with the same alphabet are statistically insignificant different ($P \geq 0.05$).

* LM; LL - ~~*Leuconostoc*~~ *L. mesenteroides*; *L*~~*actococcus*~~*. lactis*.

Figure 4. Serum and mucus lysozyme activity of Nile tilapia fed with control and probiotics supplemented diets~~experimental diets~~. Data presented as the mean ±SEM from two combined independent experiments (n=10 fish/group). Bars with different alphabet are statistically different ($P \leq 0.05$). Bars with the same alphabet are statistically insignificant different ($P \geq 0.05$). * LM; LL - *L~~euconostoc~~. mesenteroides*; *~~Lactococcus~~ L. lactis*.

Figure 5. Serum peroxidase activity of Nile tilapia fed with control and probiotics supplemented diets~~experimental diets~~. Data presented as the mean ±SEM from two combined independent experiments (n=10 fish/group). Bars with different alphabet are statistically different ($P \leq 0.05$). Bars with the same alphabet are statistically insignificant different ($P \geq 0.05$). * LM; LL - *~~Leuconostoc~~ L. mesenteroides*; *~~Lactococcus~~ L. lactis*.

Figure 6. Superoxide dismutase activity of Nile tilapia fed with control and probiotics supplemented dietsexperimental diets. Data presented as the mean ±SEM from two combined independent experiments (n=10 fish/group). Bars with different alphabet are statistically different ($P \leq 0.05$). Bars with the same alphabet are statistically insignificant different ($P \geq 0.05$). * LM; LL - - *Leuconostoc L. mesenteroides*; *Lactococcus L. lactis*.

Figure 7. The effect of probiotics supplemented diets ~~experimental diet~~ on mucus secretion. Data presented as the mean ±SEM from two combined independent experiments (n=10 fish/group). Bars with different alphabet are statistically different ($P \leq 0.05$). Bars with the same alphabet are statistically insignificant different ($P \geq 0.05$). * LM; LL - - ~~*Leuconostoc*~~ *L. mesenteroides*; *L~~actococcus~~. lactis*.

Discussion

An increasing body of research has illustrated the application of single lactic acid bacteria species as a probiotic treatment to enhance growth performance, immune response, feed utilization and gut microbiota modulation in fish [29-31]. However, there has been limited focus on exploring the potential ~~synergistic~~ effects that may arise from the concurrent oral administration of multiple LAB species to fish [3, 32-34]. Hence, this study was carried out to assess the dietary effect of ~~*Leuconostoc*~~ *L. mesenteroides* and ~~*Lactococcus*~~ *L. lactis* on the growth performance, immunity and gut microbiota of Nile tilapia ~~(*Oreochromis niloticus*)~~. The findings of this study suggest that administering both ~~*Leuconostoc*~~ *L. mesenteroides* and ~~*Lactococcus*~~ *L. lactis* into supplementation diet significantly enhances the growth performance of Nile tilapia compared to the control group over a 8 weeks period. This outcome is consistent with previous research demonstrating that probiotic supplementation, particularly with

Lactobacillus species, improves the growth performance of various fish species, including olive flounder (*P. olivaceus*) [33], Nile tilapia (*Oreochromis sp*) [35], gilthead sea bream (*Sparus aurata*) [36]. Furthermore, *L. lactis* has been found to enhance the growth performance of olive flounder ~~(*Paralichthys olivaceus*)~~ [37] and grouper (*Epinephelus coioides*) [38]. Additionally, the results obtained in this study showed that fish fed a mixture of ~~*Leuconostoc*~~ *L. mesenteroides* and ~~*Lactococcus*~~ *L. lactis* exhibited higher final weight, weight gain, and specific growth rate compared to the groups fed the control ~~diet~~and single probiotic diets. Similarly, Allameh, Yusoff [22] reported that administering multistrain probiotics, particularly *L*~~*euconostoc*~~*. mesenteroides*, enhanced the growth performance of Javanese carp (*Puntius gonionotus*). In the current study, fish fed the diet supplemented with a mixture of *L*~~*euconostoc*~~*. mesenteroides* and ~~*Lactococcus*~~ *L. lactis* showed significant improvements in feed intake and feed utilization parameters. This observation suggests that the probiotic supplement may have influenced the palatability of the feed, consequently resulting in slight increases in the growth rate and feed intake of fish. To the best of our knowledge, this study represents the first dietary incorporation of *L*~~*euconostoc*~~*. mesenteroides* and ~~*Lactococcus*~~ *L. lactis* leading to enhanced growth and feed utilization in Nile tilapia. Xia, Cao [39] proposed that probiotics primarily influence Nile tilapia by augmenting host nutrition, thereby stimulating digestive enzymes and ultimately leading to enhanced growth and feed efficiency ratios. Moreover, the colonization of probiotic microorganisms in the intestine enhances microbial equilibrium, subsequently improving feed utilization and nutrient absorption [40-42].

Improvements in growth performance and feed utilization may also be attributed to enhanced intestinal digestive functions in fish owing to probiotic supplementation (~~*Leuconostoc*~~ *L. mesenteroides and* ~~*Lactococcus*~~ *L. lactis*), which likely includes increased activities of digestive enzymes. Previous studies have shown that supplementation with *Lactobacillus* species leads to improved nutrient digestibility [9]. It is well-established that the activity of digestive enzymes is positively correlated with the digestive capacity of fish [43], thereby enhancing their ability to

extract nutrients from food [44]. It has been reported that digestive organs are highly sensitive to food composition, leading to immediate changes in the activities of digestive enzymes ultimately affecting fish growth and health [45]., Furthermore, bacteria secrete proteases to cleave peptide bonds in proteins, breaking them down into free amino acids and constituent monomers, thereby improving the animal's nutritional status. Bacterial enzymatic hydrolysis has been demonstrated to improve the bioavailability of lipids, protein and dry matter [46], potentially leading to the increased growth and nutrient utilization observed in this study.

The current study revealed a significant enhancement in both total intestinal bacteria and lactic acid bacteria counts, further supporting the improvements observed in growth and feed utilization. Similar to the findings of Mohapatra, Chakraborty [19], a substantial increase in total bacterial counts was noted when fish were fed diets supplemented with probiotics. This increase could potentially lead to improved fish health and immunity, thereby promoting growth. It has been documented that the colonization rate of bacteria in the digestive tract is directly influenced by the level of dietary bacteria intake [47]. In our investigation, the elevated adherence of microorganisms introduced via dietary supplementation could explain the improved nutrient utilization and growth observed in the fish. Numerous studies have suggested that probiotics function by colonizing the host and secreting various growth-promoting compounds ~~nutrients~~ [48] [49].

Previous studies have documented the crucial interplay between intestinal epithelial cells and probiotics in regulating the mucosal immunity of the fish gut. This interaction modulates both the physical and immunological barrier functions of the intestine and stimulates an immune response [50-54].

The results of the current investigation also indicate that the dietary combination of both *L~~euconostoc~~. mesenteroides* and *L~~actococcus~~. lactis* led to an enhanced immune response. These improvements included increased lysozyme and bactericidal activities in both serum and mucus, as well as enhancements in the alternative complement pathway, superoxide dismutase, and

Field Code Changed

Formatted: Font: 12 pt, Not Italic

Formatted: Font: 12 pt, Not Italic

peroxidase activities. Likewise, the beneficial effect of combined probiotics in the diet have been documented to trigger innate immunity in rainbow trout (*Oncorhynchus mykiss*) [55], *Labeo rohita* [11], olive flounder ~~(*P. olivaceus*)~~ [33]. Others reported that combined utilization of bacterial strains with complementary properties within the gut environment can enhance or extend the favorable health-promoting effects and immune response of the host [56]. Several authors have reported that external factors such as probiotics, vitamins, and nutrients influence the activity of the alternative complement pathway in fish [57, 58]. In the present study, the activity of the alternative complement pathway emerged as one of the immune system parameters most affected, showing a significant increase in all of the ~~treatment~~ experimental groups. These findings are similar to those described by Giri, Sukumaran [32] and Sun, Yang [38].

It was reported that bactericidal activity is a critical component of the host's defence mechanisms against pathogens [9, 59]. The outcomes obtained in our study indicate that the greatest levels of serum and mucus bactericidal activity were observed in fish receiving diets supplemented with ~~*Leuconostoc*~~ *L. mesenteroides* or/and *L~~actococcus~~. lactis*. The lysozyme activities in fish have been shown to be influenced by various probiotic species. For instance, in brown trout (*Salmo trutta*), supplementation with ~~*Leuconostoc*~~ *L. mesenteroides* has been reported to affect lysozyme activity [53]. Similarly, in grouper (*Epinephelus coioides*), supplementation with *L~~actococcus~~. lactis* has been found to modulate lysozyme activity [38]. Consistent with previous findings, in our study, the probiotics *L.~~euconostoc~~ mesenteroides* and ~~*Lactococcus*~~ *L.* lactis have altered the serum lysozyme activity of Nile tilapia ~~(*Oreochromis niloticus*)~~. The ~~*Leuconostoc*~~ *L. mesenteroides* and *L~~actococcus~~. lactis* resulted in increased serum peroxidase values, while the peroxidase content remained unaffected by the mixture diet of ~~*Leuconostoc*~~ *L. mesenteroides* and ~~*Lactococcus*~~ *L. lactis*, which supports earlier observation in sea bream (*Sparus aurata L*) [26].

Superoxide dismutase facilitates the conversion of highly reactive superoxide radicals into less reactive hydrogen peroxide and plays a crucial role in the primary antioxidant defense

mechanisms against oxidative stress. In our study, there was a significant enhancement in superoxide dismutase activities in the experimental group. Comparable findings were reported in *L.abeo rohita*, where dietary supplementation with *Bacillus subtilis* in combination with *Pseudomonas aeruginosa* and *Lactobacillus plantarum*, resulted in similar improvements [32]. Fish possess a distinctive physical barrier comprising epidermal mucus and skin [60], which serves as a component of their nonspecific defense mechanisms, protecting the fish body from direct exposure to environmental stressors and pollutants in the surrounding water. The findings of this study demonstrated an increased secretion of mucus in all fish fed diets supplemented with *Leuconostoc L. mesenteroides* or/and *Lactococcus L. lactis* compared to those fed the control diet. Likewise, it has been shown that oral administration of Lactobacillus sp., enhance skin mucus secretion in red sea bream (Pagrus major) and gilthead sea bream (Sparus aurata) [26, 61].

Together current findings showed that the diet supplemented with *Leuconostoc L. mesenteroides* and *Lactococcus L. lactis* species exerted more pronounced effects on the innate immune system of Nile tilapia. Consequently, formulations containing multiple probiotic strains were observed to elicit distinct responses in the host immune system compared to formulations containing single probiotic strains. The evidence from this study indicates that in higher vertebrates, well-designed formulations of multistrain or multispecies probiotics have been shown to exhibit health-promoting effects that are lacking in diets containing single-species probiotics [62, 63]. The effects of probiotics vary widely and are contingent upon factors such as their sources, dosage, types, and supplementation period. Therefore, when formulating a multi species probiotic mixture, it is essential to thoroughly select candidate strains to optimize their synergistic effects.

The combination of *Leuconostoc L. mesenteroides* and *Lactococcus L. lactis* appears to be a suitable mixture in this context. The synergistic effect enabled by the mixture of *Leuconostoc L.*

Formatted: Font: (Default) Times New Roman, 12 pt, Not Italic, English (United States)

Formatted: Font: (Default) Times New Roman, 12 pt, Not Italic

Formatted: Font: (Default) Times New Roman, 12 pt, Not Italic, English (United States)

Formatted: Font: (Default) Times New Roman, 12 pt, Not Italic

Formatted: Font: (Default) Times New Roman, 12 pt, Not Italic

Formatted: Font: (Default) Times New Roman, 12 pt, Not Italic, English (United States)

Formatted: Font: (Default) Times New Roman, 12 pt, Not Italic

Formatted: Font: (Default) Times New Roman, 12 pt, Not Italic

mesenteroides and ~~*Lactococcus*~~ *L. lactis* potentially contributed to the enhanced performance of Nile tilapia. However, further research is needed to confirm this potential.

Author Contributions: Supervision, conceptualization, writing—original draft preparation, writing—review and editing S.B., K.K.; methodology, validation, formal analysis, A.P., A.N., G.N., N.A., A.A., F.Z., G.Z., Zh.K., S.I., M.K., Z.S., Zh.D., Y.K., R.S., B.A., Y.M., A.S., A.A.

All authors have read and agreed to the published version of the manuscript.

~~**Funding:** This research was funded by the Science Committee of the Ministry of Science and Higher Education of the Republic of Kazakhstan (grant no. AP19576848)~~

Data Availability Statement: The original data presented in the study are openly available in FigShare at 10.6084/m9.figshare.27050416 ~~The datasets containing all data analyzed, supporting the results of this study will be available upon request from corresponding author~~.

Formatted: Font: Not Bold

Formatted: English (United States)

Formatted: English (United States)

References

1. Tan, H.Y., S.W. Chen, and S.Y. Hu, *Improvements in the growth performance, immunity, disease resistance, and gut microbiota by the probiotic Rummeliibacillus stabekisii in Nile tilapia (Oreochromis niloticus).* Fish Shellfish Immunol, 2019. **92**: p. 265-275.
2. Wang, M. and M. Lu, *Tilapia polyculture: a global review.* Aquaculture Research, 2016. **47**(8): p. 2363-2374.
3. Won, S., et al., *Effects of Bacillus subtilis WB60 and Lactococcus lactis on growth, immune responses, histology and gene expression in Nile tilapia, Oreochromis niloticus.* Microorganisms, 2020. **8**(1).
4. Defoirdt, T., et al., *Alternatives to antibiotics to control bacterial infections: luminescent vibriosis in aquaculture as an example.* Trends in biotechnology, 2007. **25**(10): p. 472-479.
5. Harikrishnan, R. and C. Balasundaram, *Modern Trends in Aeromonas hydrophila Disease Management with Fish.* Reviews in Fisheries Science, 2005. **13**(4): p. 281-320.
6. Dawood, M.A., S. Koshio, and M.Á. Esteban, *Beneficial roles of feed additives as immunostimulants in aquaculture: a review.* Reviews in Aquaculture, 2018. **10**(4): p. 950-974.
7. Nayak, S.K. and S.C. Mukherjee, *Screening of gastrointestinal bacteria of Indian major carps for a candidate probiotic species for aquaculture practices.* Aquaculture Research, 2011. **42**(7): p. 1034-1041.
8. Liu, H., et al., *Isolation of a putative probiotic strain S12 and its effect on growth performance, non-specific immunity and disease-resistance of white shrimp, Litopenaeus vannamei.* Fish & shellfish immunology, 2014. **41**(2): p. 300-307.
9. Dawood, M.A., et al., *Interaction effects of dietary supplementation of heat-killed Lactobacillus plantarum and β-glucan on growth performance, digestibility and immune response of juvenile red sea bream, Pagrus major.* Fish & shellfish immunology, 2015. **45**(1): p. 33-42.
10. Harikrishnan, R., C. Balasundaram, and M.-S. Heo, *Impact of plant products on innate and adaptive immune system of cultured finfish and shellfish.* Aquaculture, 2011. **317**(1): p. 1-15.
11. Devi, G., et al., *Effect of symbiotic supplemented diet on innate-adaptive immune response, cytokine gene regulation and antioxidant property in Labeo rohita against Aeromonas hydrophila.* Fish & Shellfish Immunology, 2019. **89**: p. 687-700.
12. Devi, G., et al., *Comparative immunostimulatory effect of probiotics and prebiotics in Channa punctatus against Aphanomyces invadans.* Fish & Shellfish Immunology, 2019. **86**: p. 965-973.
13. Merrifield, D.L., et al., *Indigenous lactic acid bacteria in fish and crustaceans.* Aquaculture nutrition: gut health, probiotics and prebiotics, 2014: p. 128-168.
14. Šušković, J., et al., *Antimicrobial activity–the most important property of probiotic and starter lactic acid bacteria.* Food Technology and Biotechnology, 2010. **48**(3): p. 296-307.
15. Seal, B.S., et al., *Alternatives to antibiotics: a symposium on the challenges and solutions for animal production.* Animal Health Research Reviews, 2013. **14**(1): p. 78-87.
16. Dalié, D., A. Deschamps, and F. Richard-Forget, *Lactic acid bacteria–Potential for control of mould growth and mycotoxins: A review.* Food control, 2010. **21**(4): p. 370-380.
17. Ringø, E., et al., *Probiotics, lactic acid bacteria and bacilli: interesting supplementation for aquaculture.* Journal of Applied Microbiology, 2020. **129**(1): p. 116-136.
18. Ray, A.K., K. Ghosh, and E. Ringø, *Enzyme-producing bacteria isolated from fish gut: a review.* Aquaculture nutrition, 2012. **18**(5): p. 465-492.
19. Mohapatra, S., et al., *Use of different microbial probiotics in the diet of rohu, Labeo rohita fingerlings: effects on growth, nutrient digestibility and retention, digestive enzyme activities and intestinal microflora.* Aquaculture Nutrition, 2012. **18**(1): p. 1-11.
20. Ausems, E.J., *THE EUROPEAN CONVENTION FOR THE PROTECTION OF VERTEBRATE ANIMALS USED FOR EXPERIMENTAL AND OTHER SCIENTIFIC PURPOSES.* Zeitschrift Fur Versuchstierkunde, 1986. **28**(5): p. 219-219.
21. Ali, Y., et al., *Classification of lytic bacteriophages attacking dairy Leuconostoc starter strains.* Applied and environmental microbiology, 2013. **79**(12): p. 3628-3636.

Formatted: Font: (Default) Times New Roman, 12 pt, Not Italic, Do not check spelling or grammar

22. Allameh, S.K., et al., *Effects of dietary mono- and multiprobiotic strains on growth performance, gut bacteria and body composition of Javanese carp (Puntius gonionotus, Bleeker 1850).* Aquaculture Nutrition, 2016. **22**(2): p. 367-373.
23. Ramos, M.A., et al., *Dietary probiotic supplementation improves growth and the intestinal morphology of Nile tilapia.* Animal, 2017. **11**(8): p. 1259-1269.
24. Yano, T., *Assay of hemolytic complement activity.* Techniques in fish immunology, 1992: p. 131-141.
25. Dawood, M.A., et al., *Effects of dietary supplementation of Lactobacillus rhamnosus or/and Lactococcus lactis on the growth, gut microbiota and immune responses of red sea bream, Pagrus major.* Fish Shellfish Immunol, 2016. **49**: p. 275-85.
26. Salinas, I., et al., *Monospecies and multispecies probiotic formulations produce different systemic and local immunostimulatory effects in the gilthead seabream (Sparus aurata L.).* Fish & shellfish immunology, 2008. **25**(1-2): p. 114-123.
27. Sun, Y.-Z., et al., *Probiotic applications of two dominant gut Bacillus strains with antagonistic activity improved the growth performance and immune responses of grouper Epinephelus coioides.* Fish & shellfish immunology, 2010. **29**(5): p. 803-809.
28. Bayantassova, S., et al., *Knowledge, attitude and practice (KAP) of smallholder farmers on foot-and-mouth disease in Cattle in West Kazakhstan.* Vet Med Sci, 2023. **9**(3): p. 1417-1425.
29. Sun, Y., et al., *Effects of dietary administration of Lactococcus lactis HNL12 on growth, innate immune response, and disease resistance of humpback grouper (Cromileptes altivelis).* Fish & shellfish immunology, 2018. **82**: p. 296-303.
30. Hooshyar, Y., et al., *Effects of Lactobacillus rhamnosus ATCC 7469 on Different Parameters Related to Health Status of Rainbow Trout (Oncorhynchus mykiss) and the Protection Against Yersinia ruckeri.* Probiotics Antimicrob Proteins, 2020. **12**(4): p. 1370-1384.
31. Adeshina, I., M.I. Abubakar, and B.E. Ajala, *Dietary supplementation with Lactobacillus acidophilus enhanced the growth, gut morphometry, antioxidant capacity, and the immune response in juveniles of the common carp, Cyprinus carpio.* Fish Physiol Biochem, 2020. **46**(4): p. 1375-1385.
32. Giri, S.S., et al., *Effects of dietary supplementation of potential probiotic Bacillus subtilis VSG1 singularly or in combination with Lactobacillus plantarum VSG3 or/and Pseudomonas aeruginosa VSG2 on the growth, immunity and disease resistance of Labeo rohita.* Aquaculture Nutrition, 2014. **20**(2): p. 163-171.
33. Beck, B.R., et al., *The effects of combined dietary probiotics Lactococcus lactis BFE920 and Lactobacillus plantarum FGL0001 on innate immunity and disease resistance in olive flounder (Paralichthys olivaceus).* Fish Shellfish Immunol, 2015. **42**(1): p. 177-83.
34. Zhang, H., et al., *Effect of Dietary Supplementation of Lactobacillus Casei YYL3 and L. Plantarum YYL5 on Growth, Immune Response and Intestinal Microbiota in Channel Catfish.* Animals (Basel), 2019. **9**(12).
35. Xia, Y., et al., *Effects of dietary Lactobacillus rhamnosus JCM1136 and Lactococcus lactis subsp. lactis JCM5805 on the growth, intestinal microbiota, morphology, immune response and disease resistance of juvenile Nile tilapia, Oreochromis niloticus.* Fish Shellfish Immunol, 2018. **76**: p. 368-379.
36. Suzer, C., et al., *Lactobacillus spp. bacteria as probiotics in gilthead sea bream (Sparus aurata, L.) larvae: Effects on growth performance and digestive enzyme activities.* Aquaculture, 2008. **280**(1): p. 140-145.
37. Heo, W.-S., et al., *Effects of dietary probiotic, Lactococcus lactis subsp. lactis I2, supplementation on the growth and immune response of olive flounder (Paralichthys olivaceus).* Aquaculture, 2013. **376-379**: p. 20-24.
38. Sun, Y.-Z., et al., *Effect of Lactococcus lactis and Enterococcus faecium on growth performance, digestive enzymes and immune response of grouper Epinephelus coioides.* Aquaculture Nutrition, 2012. **18**(3): p. 281-289.
39. Xia, Y., et al., *Effects of Lactococcus lactis subsp. lactis JCM5805 on colonization dynamics of gut microbiota and regulation of immunity in early ontogenetic stages of tilapia.* Fish & Shellfish Immunology, 2019. **86**: p. 53-63.

40. Adeoye, A.A., et al., *Combined effects of exogenous enzymes and probiotic on Nile tilapia (Oreochromis niloticus) growth, intestinal morphology and microbiome.* Aquaculture, 2016. **463**: p. 61-70.
41. Lara-Flores, M. and M.A. Olvera-Novoa, *The use of lactic acid bacteria isolated from intestinal tract of Nile tilapia (Oreochromis niloticus), as growth promoters in fish fed low protein diets.* Latin American Journal of Aquatic Research, 2017. **41**(3): p. 490-497.
42. Lara-Flores, M., et al., *Use of the bacteria Streptococcus faecium and Lactobacillus acidophilus, and the yeast Saccharomyces cerevisiae as growth promoters in Nile tilapia (Oreochromis niloticus).* Aquaculture, 2003. **216**(1): p. 193-201.
43. Hakim, Y., et al., *Relationship between intestinal brush border enzymatic activity and growth rate in tilapias fed diets containing 30% or 48% protein.* Aquaculture, 2006. **257**(1-4): p. 420-428.
44. Furne, M., et al., *Digestive enzyme activities in Adriatic sturgeon Acipenser naccarii and rainbow trout Oncorhynchus mykiss. A comparative study.* Aquaculture, 2005. **250**(1-2): p. 391-398.
45. Shan, X., et al., *Effects of photoperiod on growth, mortality and digestive enzymes in miiuy croaker larvae and juveniles.* Aquaculture, 2008. **281**(1-4): p. 70-76.
46. Ling, W.H. and O. Hänninen, *Shifting from a conventional diet to an uncooked vegan diet reversibly alters fecal hydrolytic activities in humans.* The Journal of nutrition, 1992. **122**(4): p. 924-930.
47. Bagheri, T., et al., *Growth, survival and gut microbial load of rainbow trout (Onchorhynchus mykiss) fry given diet supplemented with probiotic during the two months of first feeding.* Turkish Journal of Fisheries and Aquatic Sciences, 2008(1): p. 43-48.
48. Carnevali, O., F. Maradonna, and G. Gioacchini, *Integrated control of fish metabolism, wellbeing and reproduction: The role of probiotic.* Aquaculture, 2017. **472**: p. 144-155.
49. Iorizzo, M., et al., *Probiotic Potentiality from Versatile Lactiplantibacillus plantarum Strains as Resource to Enhance Freshwater Fish Health.* Microorganisms, 2022. **10**(2).
50. Thomas, C.M. and J. Versalovic, *Probiotics-host communication: modulation of signaling pathways in the intestine.* Gut microbes, 2010. **1**(3): p. 148-163.
51. Wells, J.M., *Immunomodulatory mechanisms of lactobacilli.* Microbial cell factories, 2011. **10**(Suppl 1): p. S17.
52. Liu, W., et al., *Comparison of adhesive gut bacteria composition, immunity, and disease resistance in juvenile hybrid tilapia fed two different Lactobacillus strains.* Fish & shellfish immunology, 2013. **35**(1): p. 54-62.
53. Balcazar, J.L., et al., *Changes in intestinal microbiota and humoral immune response following probiotic administration in brown trout (Salmo trutta).* Br J Nutr, 2007. **97**(3): p. 522-7.
54. Seisenov, B., et al., *In Vitro Fertilization in Kazakh Whiteheaded Cattle: A Comparative Study.* Life (Basel), 2023. **13**(8): p. 1632.
55. Jasim, S.A., et al., *The synergistic effects of the probiotic (Lactobacillus fermentum) and cinnamon, Cinnamomum sp. powder on growth performance, intestinal microbiota, immunity, antioxidant defence and resistance to Yersinia ruckeri infection in the rainbow trout (Oncorhynchus mykiss) under high rearing density.* Aquaculture Research, 2022. **53**(17): p. 5957-5970.
56. Salinas, I., et al., *Dietary administration of Lactobacillus delbrüeckii and Bacillus subtilis, single or combined, on gilthead seabream cellular innate immune responses.* Fish & Shellfish Immunology, 2005. **19**(1): p. 67-77.
57. Cerezuela, R., et al., *Effects of dietary Bacillus subtilis, Tetraselmis chuii, and Phaeodactylum tricornutum, singularly or in combination, on the immune response and disease resistance of sea bream (Sparus aurata L.).* Fish & Shellfish Immunology, 2012. **33**(2): p. 342-349.
58. Mohammadian, T., et al., *Synbiotic effects of β-glucan, mannan oligosaccharide and Lactobacillus casei on growth performance, intestine enzymes activities, immune-hematological parameters and immune-related gene expression in common carp, Cyprinus carpio: An experimental infection with Aeromonas hydrophila.* Aquaculture, 2019. **511**: p. 634197.
59. Issimov, A., et al., *Prevalence of Clostridium perfringens and Detection of Its Toxins in Meat Products in Selected Areas of West Kazakhstan.* Agriculture-Basel, 2022. **12**(9).

60. Hernandez, L.H.H., et al., *Effects of the commercial probiotic Lactobacillus casei on the growth, protein content of skin mucus and stress resistance of juveniles of the Porthole livebearer Poecilopsis gracilis (Poecilidae).* Aquaculture Nutrition, 2010. **16**(4): p. 407-411.
61. Dawood, M.A.O., et al., *Effects of dietary inactivated Pediococcus pentosaceus on growth performance, feed utilization and blood characteristics of red sea bream, Pagrus major juvenile.* Aquaculture Nutrition, 2016. **22**(4): p. 923-932.
62. Timmerman, H.M., et al., *Monostrain, multistrain and multispecies probiotics—A comparison of functionality and efficacy.* International Journal of Food Microbiology, 2004. **96**(3): p. 219-233.
63. Wang, Y., et al., *Probiotic Potential of Leuconostoc pseudomesenteroides and Lactobacillus Strains Isolated From Yaks.* Frontiers in Microbiology, 2018. **9**.

Formatted: Font: (Default) Times New Roman, 12 pt

Response to Reviewers comments

Reviewer #1:

Dear authors,
The study is interesting using two LAB as feed additive in nile tilapia farming. However, the major concerns about the paper are as below:

1. the paper has too many minor errors such scientific name and table format
AU response: Revised as requested throughout the text.

2. Comments from PDF file, line 33: provide actual p value, check in whole manuscript
AU response: Revised as requested throughout the text.

3. Comments from PDF file, line 36: provide the value of the mentioned parameters
AU response: Revised as requested throughout the text.

4. Comments from PDF file: may provide importance of nile tilapia in a paragraph?
AU response: It is now revised as requested in lines 55-58.

5. Comments from PDF file: please elaborate the importance of LAB in a paragraph
AU response: It is now revised as requested in lines 100-104.

4. Comments from PDF file, line 81: any ref to support total number of replicate?
AU response: We applied two replicates per treatment to maintain experimental feasibility and ensure consistency in the controlled environment. The study's sample size was chosen based on established practices in similar aquaculture research, allowing for sufficient statistical power while minimizing variability. Additionally, our preliminary study demonstrated that two replicates can provide reliable and meaningful data in aquaculture experiments of this scale.

5. Comments from PDF file, line 87: how to maintain?
AU response: Tanks with fish were kept in facilities equipped with climate control system adjusted to 27 degree. Built-in thermostats were also used to ensure precise temperature control.

6. Comments from PDF file, line 91: how to measure?
AU response: Thank you for your comment. The methods for measuring survival rate, individual body weight and length, immune parameters, and gut microbiota composition are described in detail in the subsequent sections of the manuscript. We kindly refer you to those sections for a thorough explanation of the procedures used.

- survival rate – line 212-213.
- individual body weight – line 215.
- gut microbiota – lines 228-238.
- immune parameters – line 241-275.

7. Comments from PDF file, line 98: any reason using this temperature?
AU response: Thank you for your comment. The temperature of 37°C was chosen because it is a widely validated and standard incubation temperature for culturing lactic acid bacteria, as it closely mimics the optimal growth conditions for these microorganisms.
Here are some reliable references that support the use of 37°C for culturing lactic acid bacteria:
1. Hammes, W. P., & Hertel, C. (2006). The genera lactobacillus and carnobacterium. The prokaryotes, 4, 320-403.

2. Savadogo, A., Ouattara, A. C., Bassole, H. I., & Traore, S. A. (2006). Bacteriocins and lactic acid bacteria-a minireview. African journal of biotechnology, 5(9).

8. Comments from PDF file, line 117: Check
AU response: Revised as requested in line 190.

9. Comments from PDF file, Table 1: please use a standard table format
AU response: Revised as requested

10. Comments from PDF file, Table 1: Other?
AU response: Thank you for your comment. The diet pattern used, including its ingredients and proximate composition, follows widely accepted formulations from relevant publications and industry practices. The current study focused on the effects of the diet as formulated, and no other compositions were included or referenced.

11. Comments from PDF file, line 177: How about CAT and GPx?
AU response: We appreciate the reviewer's suggestion regarding the inclusion of oxidative stress indicators such as catalase and glutathione peroxidase. However, the evaluation of these specific oxidative stress markers was not part of the original study design and was therefore not planned or assessed. Our study focused on other key immune and physiological parameters, and while CAT and GPx are valuable indicators, they were outside the scope of the current research objectives. Future studies may incorporate these markers to provide a more comprehensive analysis.

11. Statistical analysis should conduct HOV and normality tests before anova test
AU response: Thank you for your comment. In our study, the use of Student's t-test and one-way ANOVA with Tukey's post hoc analysis already accounts for assumptions regarding normality and homogeneity of variances. However to revise reviewers' request we run an additional Levene's and Kolmogorov-Smirnov tests to check whether this assumption holds. By conducting these tests, we can ensure that the variances did not differ significantly between groups. Please, see lines 280-281.

12.Should provide actual p value
AU response: Revised as requested

13. Should compare to single vs plural LAB analysis before can confirm plural LAB is better
AU response: We understand the concerns raised during the revision process, and we would like to clarify why we did not include an individual study results for *Leuconostoc mesenteroides* and *Lactococcus lactis* in the initial manuscript. Unfortunately, we faced a scarcity of reagents during the study, which limited our ability to perform separate experiments for each probiotic strain.The research was conducted in Kazakhstan, and the ongoing political situation in neighboring countries, specifically the Russia-Ukraine conflict, disrupted our supply chains. Lab supply distributors frequently relied on these regions as transit hubs, which led to significant delays and shortages of essential reagents.
You may have noticed a change in the total number of fish in the revised version of the manuscript. We would like to explain the adjustments made. In our original study, we used a total of 384 fish, which were divided into four groups: a control group, a group fed *Leuconostoc mesenteroides*, a group fed *Lactococcus lactis*, and a group fed a mixture of *L. mesenteroides* and *L. lactis*. However, due to significant delays in receiving lab supplies, particularly reagents necessary for analyzing the samples, we were unable to present a full comparison of the groups in

the initial submission. This created gaps in the data, preventing us from providing a comprehensive analysis of each individual probiotic's effects, as well as their combined impact. As expected, reviewers raised concerns about these missing comparisons—concerns we were already aware of and intended to address.

Since then, we have been able to complete the analyses for all groups now that the supply chain issues have been resolved. The revised version includes these additional data, allowing for a complete comparison between the control, *L. mesenteroides*, *L. lactis*, and the combined diet groups.

We ensured that all samples from the individual study were properly stored during the reagent shortage. These additional results provide a more comprehensive and robust understanding of the study.

Response to Reviewers comments

Reviewer #2:
This Ms was interesting to isolated new probiotics candidate species isolated from fish and its potential role on growth, antioxidant activity, and immune potential tested in this study in Nile tilapia. But I am not sure of these species done the ability of the probiotics characterization and its in vitro activity is very essential before testing in vivo of aquatic animals. I found some of major mistake of this Ms need to rectify before considering of the article in this journal as seen in below:

Line 22, 46: aquaculture industry growing and developed rapidly not just “two decades” you can change ‘few decades’
AU response: It is now revised as requested in lines 22, 53.

Line 22, 47: add ‘animal’ after future
AU response: It is now revised as requested in lines 23, 54.

Line 26: add ‘influxes of gut microbiota’ after fish
AU response: Revised as requested in line 26.

Line 29,30,34,35,38,40,117,192,193,186,198,211,227,232,234,391,393,401,404,406,410,418,445,461,463,484,466,467,469,480,482,492,493: ‘Leuconostoc mesenteroides and Lactococcus lactis’ change to ‘L. mesenteroides and L. lactis’
AU response: Revised as requested throughout the text.

Line 36: ‘lactis diet’ change to ‘lactis supplemented diet’
AU response: Revised as requested in line 44.

Line 39: ‘lactis groups’ change to ‘lactis supplemented diet groups’
AU response: Revised as requested in line 47.

Line 42: keywaord limit to 4 words
AU response: Revised as requested.

Line 54: Include ‘https://doi.org/10.1080/10641260500320845’
AU response: Reference has been added, see line 89.

Line 55: Include ‘https://doi.org/10.1016/j.aquaculture.2011.03.039’
AU response: Reference has been added, see line 90.

Line 56: Include ‘https://doi.org/10.1016/j.fsi.2019.04.036; https://doi.org/10.1016/j.fsi.2018.12.051’
AU response: Reference has been added, see line 91.

Line 57: ‘bacteria’ change to ‘population’
AU response: Revised as requested in line 92.

Line 58: ‘have the capability to enhance’ change to ‘were ability to increase’
AU response: Revised as requested in line 93.

Line 60: include ‘https://doi.org/10.1111/jam.14628’

AU response: Reference has been added, see line 95.

Line 61: ‘dietary’ change to ‘nutritional’
AU response: Revised as requested in line 96.

Line 66: Include ‘(Oreochromis niloticus)’ after Nile tilapia
AU response: Revised as requested in line 101.

Line 67: different fish species, specify?
AU response: Specified in line 97.

Line 68: ‘freshwater aquaculture’ change to ‘freshwater fish Nile tilapia’
AU response: Revised as requested in line 102.

Line 70: Delete ‘in Nile tilapia’
AU response: Revised as requested in line 104.

Line 82: Can check any health or clinical test before acclimatization?
AU response: We understand reviewer's concern regarding to health or clinical tests before acclimatization. However, the fish used in this study were purchased from a reputable commercial farm that provided comprehensive health certification documents, confirming the fish were disease-free and fit for research. Given the provision of these official health certificates, we deemed additional clinical testing unnecessary, as the fish had already undergone rigorous health screening.

Line 89: control diet means commercial diet or formulated diet?
AU response: Commercial diet.

Line 90: ‘24-hour’ change to ‘24-h’
AU response: Revised as requested in line 143.

Line 98: ‘48 h’ change to ’48-h’
AU response: Revised as requested in line 151.

Line 109: ’95 ◦C, 95 ◦C, 55 ◦C 72 ◦C 72 ◦C’ change to ‘95◦C, 95◦C, 55◦C 72◦C 72◦C’
AU response: Revised as requested in line 171.

Line 114: Commercial feed, trade name, company name, where the diet purchase?
AU response: Revised as requested in line 176.

Line 117-125: The subculture of probiotics and how to quantify, not clearly describe..
AU response: It is now revised as requested in lines 179-189.

Line 139: ‘24 h’ change to ’24-h’
AU response: Revised as requested in line 229.

Line 154: ‘1h’ change to ‘1-h’
AU response: Revised as requested in line 244.

Line 156: ‘10 minutes’ change to ‘10 min’

AU response: Revised as requested in line 246.

Line 161: Escherichia coli to be italics
AU response: Revised as requested in line 254.

Line 166: space between 10 and μl; Micrococcus lysodeikticus to be italics
AU response: Revised as requested in line 259.

Line 175: ‘minutes’ change to ‘min’
AU response: Revised as requested in line 267.

Line 193: ‘weight gain (WG), final body weight (FBW), and specific growth rate (SGR)’ change to ‘WG, FBW, and SGR’
AU response: Revised as requested in line 288.

Line 195: ‘protein efficiency ratio (PER) and feed efficiency ratio (FER)’ change to ‘PER and FER’
AU response: Revised as requested in line 289.

Line 202: in table 2: ‘Nile tilapia (Oreochromis niloticus) fed with Leuconostoc mesenteroides and Lactococcus lactis’ change to ‘Nile tilapia fed with L. mesenteroides and L. lactis’
AU response: Revised as requested.

Line 220,230,245,272,293,315,337,359: ‘experimental diets’ change to ‘probiotics supplemented diets’
AU response: Revised as requested throughout the text.

Line 226: ‘alternative complement pathway (ACP)’ change to ‘ACP’
AU response: Revised as requested in line 389.

Line 392,467: Remove ‘(Oreochromis niloticus)’
AU response: Revised as requested in lines 602, 699.

Line 399: L. lactis to be italics
AU response: Revised as requested in line 608.

Line 400: Remove ‘(Paralichthys olivaceus)’
AU response: Revised as requested in line 609.

Line 420: Lactobacillus to be italics
AU response: Revised as requested in line 643.

Line 439: ‘nutrients [42] [43]’ change to ‘compounds [42,43]’
AU response: Revised as requested in line 664.

Line 450: Remove ‘(P. olivaceus)’
AU response: Revised as requested in line 675.

Line 470: include the scientific name of the sea bream

AU response: Revised as requested in line 702.

Line 474: ‘Labeo rohita’ change to ‘L. rohita’
AU response: Revised as requested in line 706.

Line 490: ‘include ‘multi species’ before probiotic mixture
AU response: Revised as requested in line 737.

All figure are modify simple and clear for printing.
AU response: Thank you for your positive feedback regarding the figures.

Response to Reviewers comments

Reviewer #3:
Based on scientific considerations, this manuscript lacks interesting findings that contribute to the field of fish nutrition/immunology. In general, the manuscript is poorly written and contains major concerns and unclear points that the authors need to address in order to improve the quality of the manuscript and meet acceptable standards for a scientific article. In the present study, the authors fail to provide crucial information. Overall, the writing of the manuscript needs improvement, and it would be beneficial for the authors to seek guidance from expert researchers on how to write a good manuscript.

- This manuscript contains only immune parameters, and lack of data about the parameters related to growth and gut microbiota as mention in the objective.
AU response: More data on growth performance are detailed in Table 2. Additional information has been added on gut microbiota in result section.

- The experimental design is poor, I propose that authors should compare each of probiotics and see its combination.
AU response: We understand the concerns raised during the revision process, and we would like to clarify why we did not include an individual study results for *Leuconostoc mesenteroides* and *Lactococcus lactis* in the initial manuscript. Unfortunately, we faced a scarcity of reagents during the study, which limited our ability to perform separate experiments for each probiotic strain.The research was conducted in Kazakhstan, and the ongoing political situation in neighboring countries, specifically the Russia-Ukraine conflict, disrupted our supply chains. Lab supply distributors frequently relied on these regions as transit hubs, which led to significant delays and shortages of essential reagents.
You may have noticed a change in the total number of fish in the revised version of the manuscript. We would like to explain the adjustments made. In our original study, we used a total of 384 fish, which were divided into four groups: a control group, a group fed *Leuconostoc mesenteroides*, a group fed *Lactococcus lactis*, and a group fed a mixture of *L. mesenteroides* and *L. lactis*. However, due to significant delays in receiving lab supplies, particularly reagents necessary for analyzing the samples, we were unable to present a full comparison of the groups in the initial submission. This created gaps in the data, preventing us from providing a comprehensive analysis of each individual probiotic's effects, as well as their combined impact. As expected, reviewers raised concerns about these missing comparisons—concerns we were already aware of and intended to address.
Since then, we have been able to complete the analyses for all groups now that the supply chain issues have been resolved. The revised version includes these additional data, allowing for a complete comparison between the control, *L. mesenteroides*, *L. lactis*, and the combined diet groups.
We ensured that all samples from the individual study were properly stored during the reagent shortage. These additional results provide a more comprehensive and robust understanding of the study.

- The data are less, not enough for publication in this high standard journal.
AU response: Thank you for your constructive feedback. In the revised version of the manuscript, we have expanded the data by providing a detailed comparison of each probiotic (*Leuconostoc mesenteroides* and *Lactococcus lactis*) as well as their combination.

Response to Reviewers comments

Reviewer #4:
Interesting research. However,
1. please make it clearer about "multistrain" definitions, In this study, you applied ONLY two genus. maybe two strain?
AU response: "multistrain" changed to “two strain”

2. What about water quality?
AU response: Revised as requested in line 24.

3. Comments from PDF file
AU response: The reviewer's comment in line 26 has been addressed and revised as requested in line 27.

4. Comments from PDF file
AU response: The reviewer's comment in line 68 has been addressed and revised as requested in line 105.

5. Why did you apply only two replicates?
AU response: We applied two replicates per treatment to maintain experimental feasibility and ensure consistency in the controlled environment. The study's sample size was chosen based on established practices in similar aquaculture research, allowing for sufficient statistical power while minimizing variability. Additionally, our preliminary study demonstrated that two replicates can provide reliable and meaningful data in aquaculture experiments of this scale.

6. Please check format of scientific names.
AU response: Revised as requested throughout the text.

7. Bactericidal activity, in the future. Please select the ones that closer affected on the fish studies, tilapia pathogens.
AU response: Thank you for the suggestion. In future studies, we will focus on selecting bactericidal activity assays more closely related to pathogens specific to Nile tilapia. This will allow for a more targeted evaluation of the probiotics' effectiveness against species-relevant bacterial threats.

8. In this study, you cannot state "synergistic" effects because NO individual treatment applied.
AU response: We understand the concerns raised during the revision process, and we would like to clarify why we did not include an individual study results for *Leuconostoc mesenteroides* and *Lactococcus lactis* in the initial manuscript. Unfortunately, we faced a scarcity of reagents during the study, which limited our ability to perform separate experiments for each probiotic strain.The research was conducted in Kazakhstan, and the ongoing political situation in neighboring countries, specifically the Russia-Ukraine conflict, disrupted our supply chains. Lab supply distributors frequently relied on these regions as transit hubs, which led to significant delays and shortages of essential reagents.
You may have noticed a change in the total number of fish in the revised version of the manuscript. We would like to explain the adjustments made. In our original study, we used a total of 384 fish, which were divided into four groups: a control group, a group fed *Leuconostoc mesenteroides*, a group fed *Lactococcus lactis*, and a group fed a mixture of *L. mesenteroides* and *L. lactis*. However, due to significant delays in receiving lab supplies, particularly reagents

necessary for analyzing the samples, we were unable to present a full comparison of the groups in the initial submission. This created gaps in the data, preventing us from providing a comprehensive analysis of each individual probiotic's effects, as well as their combined impact. As expected, reviewers raised concerns about these missing comparisons—concerns we were already aware of and intended to address.

Since then, we have been able to complete the analyses for all groups now that the supply chain issues have been resolved. The revised version includes these additional data, allowing for a complete comparison between the control, *L. mesenteroides*, *L. lactis*, and the combined diet groups.

We ensured that all samples from the individual study were properly stored during the reagent shortage. These additional results provide a more comprehensive and robust understanding of the study.

6. Please check format of references.

AU response: Revised as requested. EndNote 20 version, Style “numbered” was used in this manuscript, moreover the font has been changed to “Times New Roman” as in the main text.
