# Supplementary material for: The dietary effects of two strain probiotics (Leuconostoc mesenteroides, Lactococcus lactis) on growth performance, immune response and gut microbiota in Nile tilapia (Oreochromis niloticus)
Source: PLoS One. 2024 Oct 24;19(10):e0312580. doi: 10.1371/journal.pone.0312580 (PMC11500904; doi:10.1371/journal.pone.0312580)
Supplement: Attachment — Submitted filename: Responce to Reviewer 4.DOCX [file pone.0312580.s003.DOCX]

Response to Reviewers comments

Reviewer #4:

Interesting research. However,
1. please make it clearer about "multistrain" definitions, In this study, you applied ONLY two genus. maybe two strain?

**AU response:** "multistrain" changed to “two strain”

2. What about water quality?

**AU response:** Revised as requested in line 24.

3. Comments from PDF file

**AU response:** The reviewer's comment in line 26 has been addressed and revised as requested in line 27.

4. Comments from PDF file

**AU response:** The reviewer's comment in line 68 has been addressed and revised as requested in line 105.

5. Why did you apply only two replicates?

**AU response:** We applied two replicates per treatment to maintain experimental feasibility and ensure consistency in the controlled environment. The study's sample size was chosen based on established practices in similar aquaculture research, allowing for sufficient statistical power while minimizing variability. Additionally, our preliminary study demonstrated that two replicates can provide reliable and meaningful data in aquaculture experiments of this scale.

6. Please check format of scientific names.

**AU response:** Revised as requested throughout the text.

7. Bactericidal activity, in the future. Please select the ones that closer affected on the fish studies, tilapia pathogens.

**AU response:** Thank you for the suggestion. In future studies, we will focus on selecting bactericidal activity assays more closely related to pathogens specific to Nile tilapia. This will allow for a more targeted evaluation of the probiotics' effectiveness against species-relevant bacterial threats.

8. In this study, you cannot state "synergistic" effects because NO individual treatment applied.

**AU response:** We understand the concerns raised during the revision process, and we would like to clarify why we did not include an individual study results for *Leuconostoc mesenteroides* and *Lactococcus lactis* in the initial manuscript. Unfortunately, we faced a scarcity of reagents during the study, which limited our ability to perform separate experiments for each probiotic strain.The research was conducted in Kazakhstan, and the ongoing political situation in neighboring countries, specifically the Russia-Ukraine conflict, disrupted our supply chains. Lab supply distributors frequently relied on these regions as transit hubs, which led to significant delays and shortages of essential reagents.

You may have noticed a change in the total number of fish in the revised version of the manuscript. We would like to explain the adjustments made. In our original study, we used a total of 384 fish, which were divided into four groups: a control group, a group fed *Leuconostoc mesenteroides*, a group fed *Lactococcus lactis*, and a group fed a mixture of *L. mesenteroides* and *L. lactis*. However, due to significant delays in receiving lab supplies, particularly reagents necessary for analyzing the samples, we were unable to present a full comparison of the groups in the initial submission. This created gaps in the data, preventing us from providing a comprehensive analysis of each individual probiotic's effects, as well as their combined impact. As expected, reviewers raised concerns about these missing comparisons—concerns we were already aware of and intended to address.

Since then, we have been able to complete the analyses for all groups now that the supply chain issues have been resolved. The revised version includes these additional data, allowing for a complete comparison between the control, *L. mesenteroides*, *L. lactis*, and the combined diet groups.

We ensured that all samples from the individual study were properly stored during the reagent shortage. These additional results provide a more comprehensive and robust understanding of the study.

6. Please check format of references.

**AU response:** Revised as requested. EndNote 20 version, Style “numbered” was used in this manuscript, moreover the font has been changed to “Times New Roman” as in the main text.
