# Supplementary material for: The dietary effects of two strain probiotics (Leuconostoc mesenteroides, Lactococcus lactis) on growth performance, immune response and gut microbiota in Nile tilapia (Oreochromis niloticus)
Source: PLoS One. 2024 Oct 24;19(10):e0312580. doi: 10.1371/journal.pone.0312580 (PMC11500904; doi:10.1371/journal.pone.0312580)
Supplement: Attachment — Submitted filename: Responce to Reviewer 1.DOCX [file pone.0312580.s005.DOCX]

Response to Reviewers comments

Reviewer #1:

Dear author,

Thank you for your work. The manuscript is look better now. However, there are few concerns as follow:

1. Incubation temperature see line 103

**AU response:** Revised as requested in line 103.

2. format for scientific name and table

**AU response:** Revised as requested throughout the text.
